# Supplementary figures and images for: Progressive cardiomyopathy with intercalated disc disorganization in a rat model of Becker dystrophy (part 1 of 2)
Source: EMBO Rep. 2024 Oct 2;25(11):4898–920. doi: 10.1038/s44319-024-00249-9 (PMC11549483; doi:10.1038/s44319-024-00249-9)

*mir34c*

*mir708*

Relative fold change expression

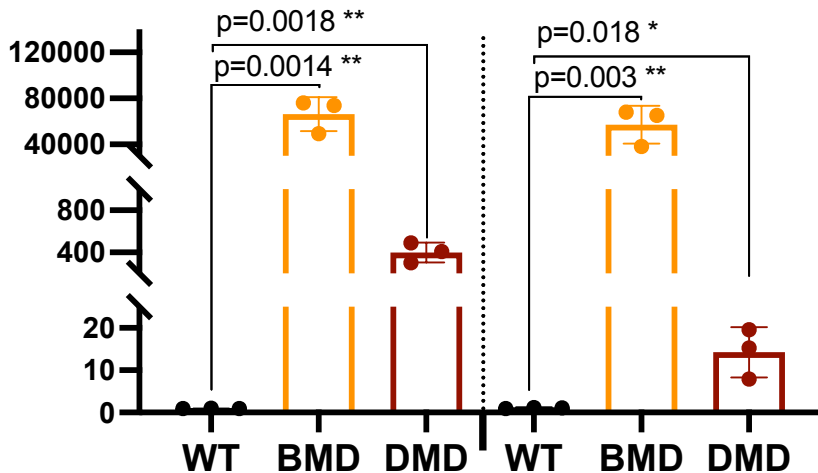

Supplement: Supplementary file 2 — Source data Fig. 1 [file 44319_2024_249_MOESM2_ESM.zip › FIG1/mir.pdf]

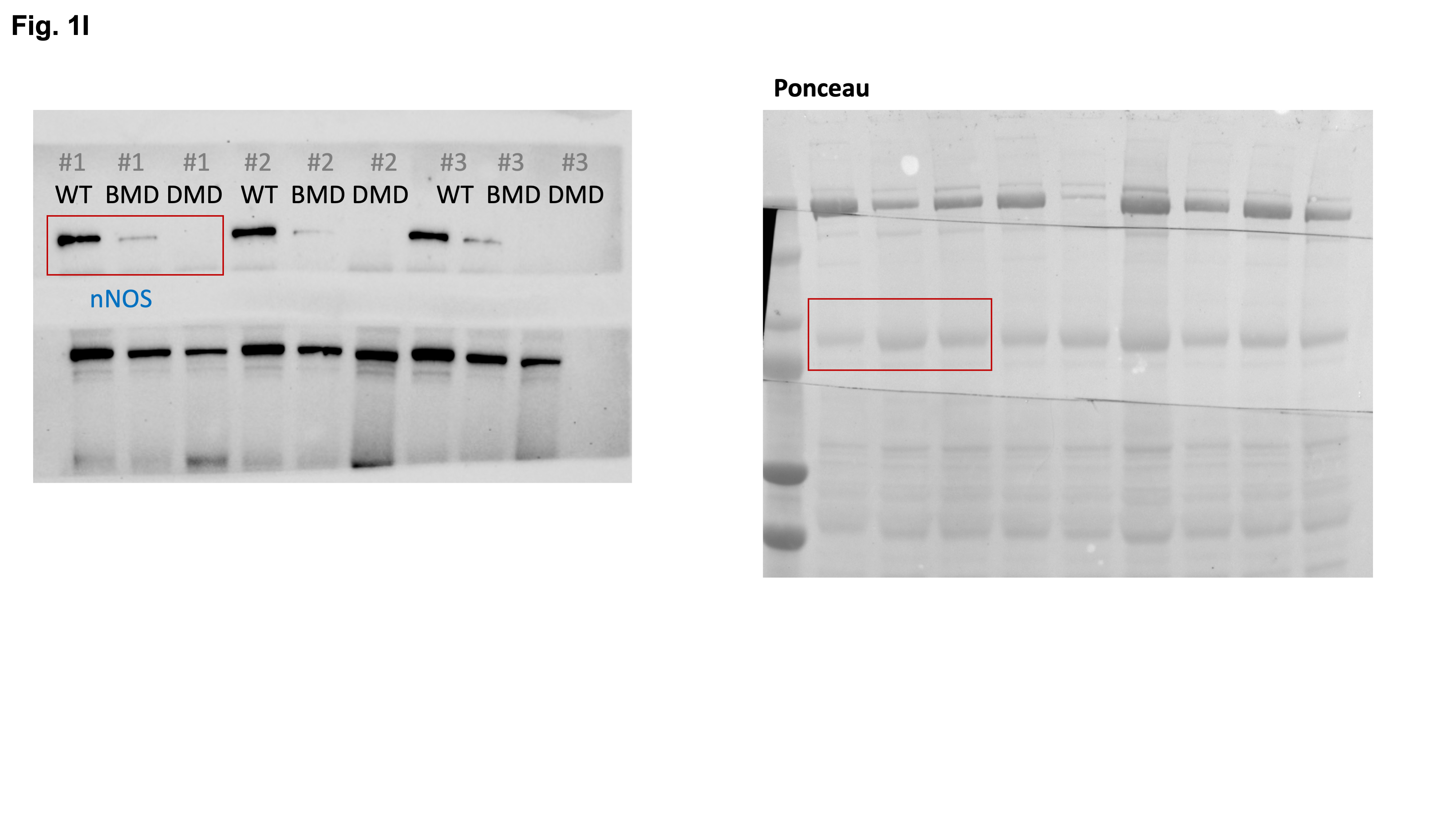

Supplement: Supplementary file 2 — Source data Fig. 1 [file 44319_2024_249_MOESM2_ESM.zip › FIG1/FIG.1I.png]

Dystrophin intensity

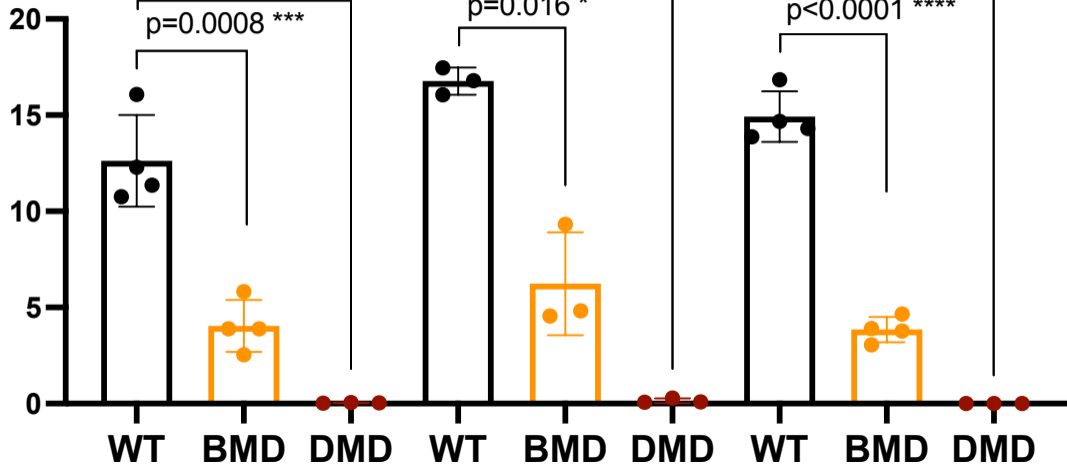

Supplement: Supplementary file 2 — Source data Fig. 1 [file 44319_2024_249_MOESM2_ESM.zip › FIG1/dys intensity.pdf]

**Fig. 1B Tibialis anterior**

SHOWN IN THE FIGURE

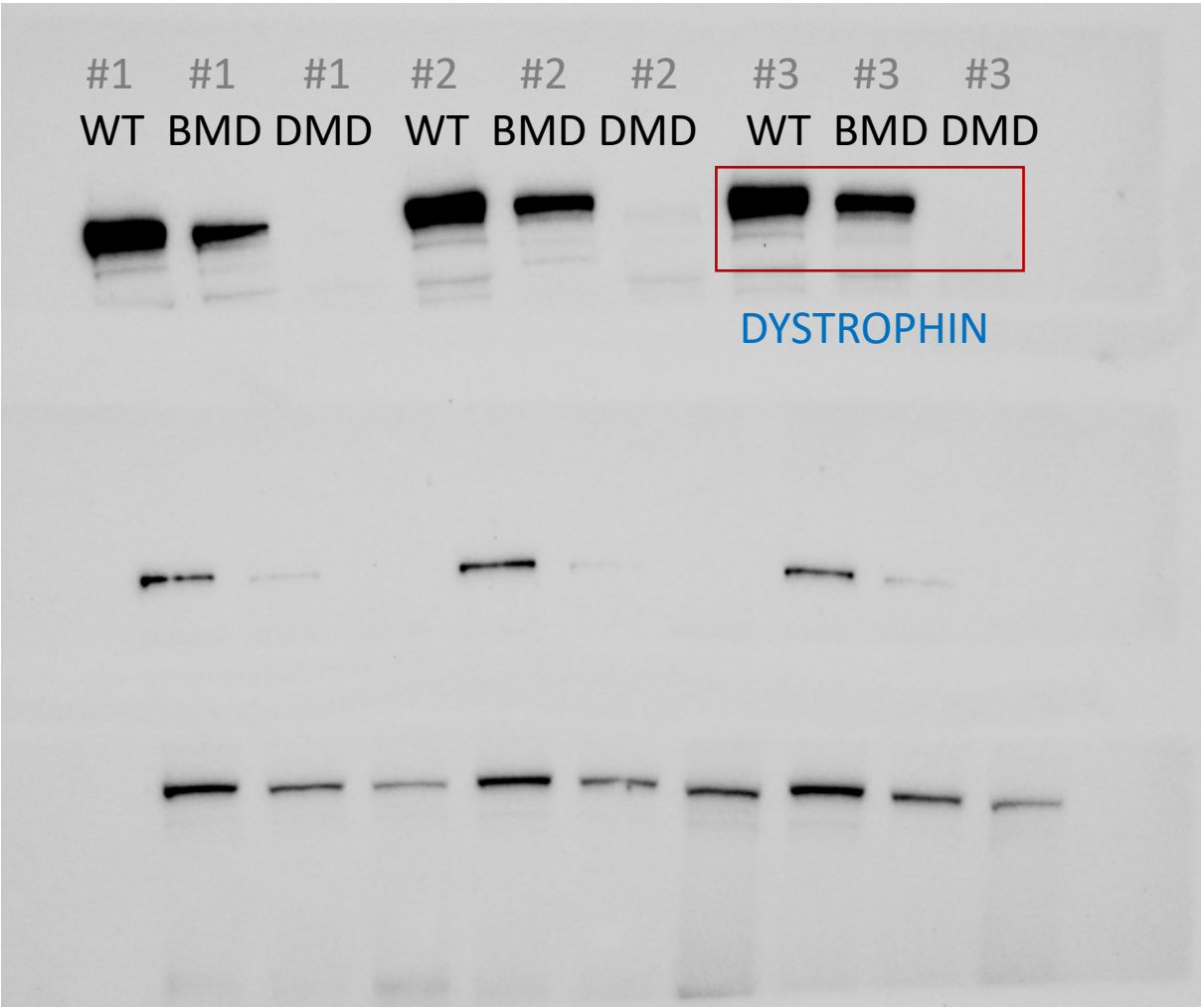

Ponceau

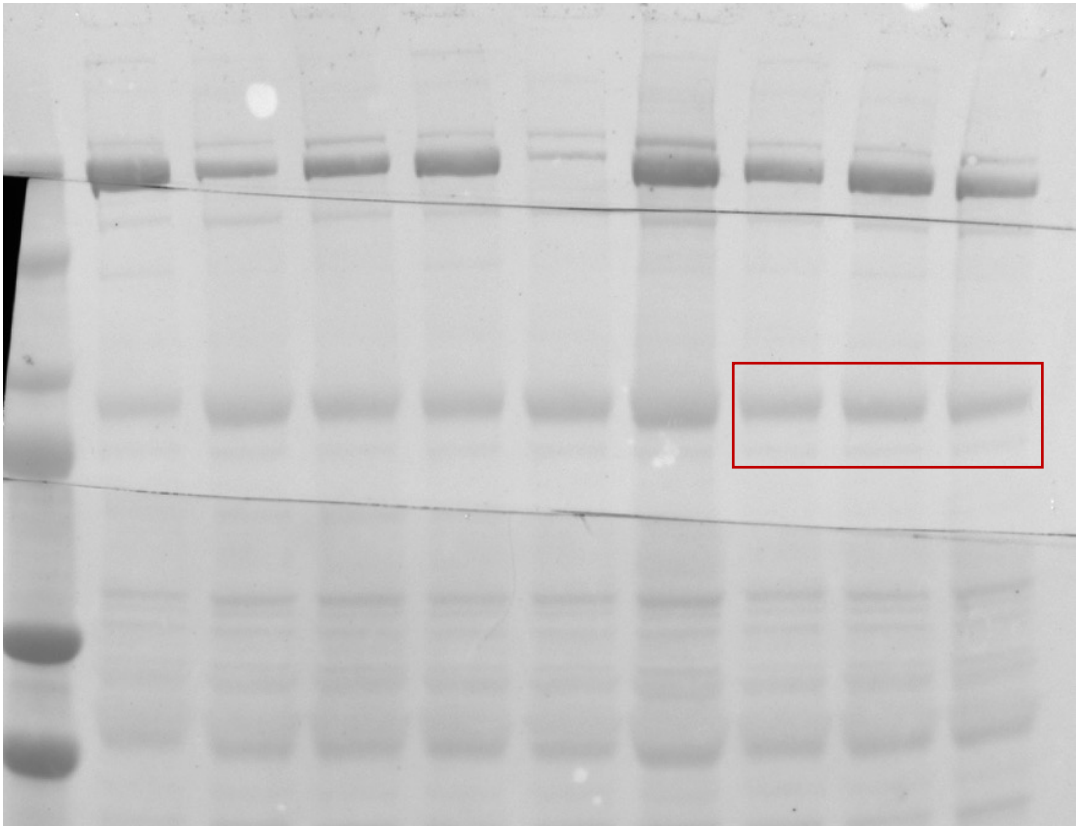

Fig. 1B Heart

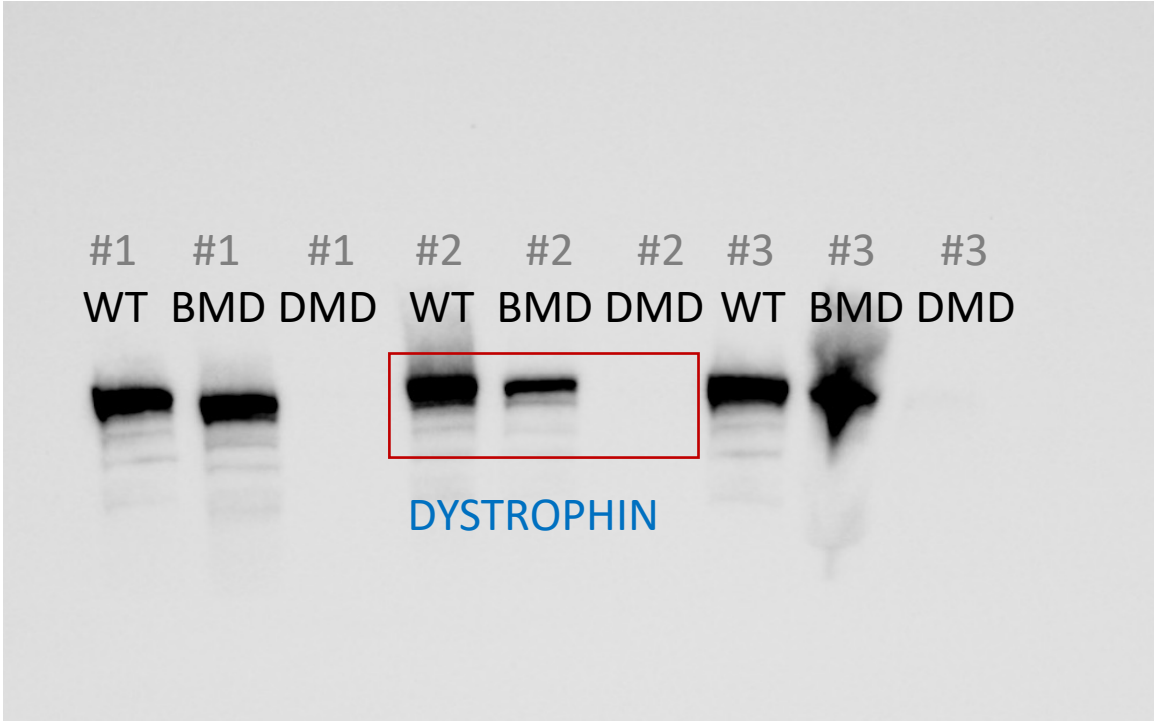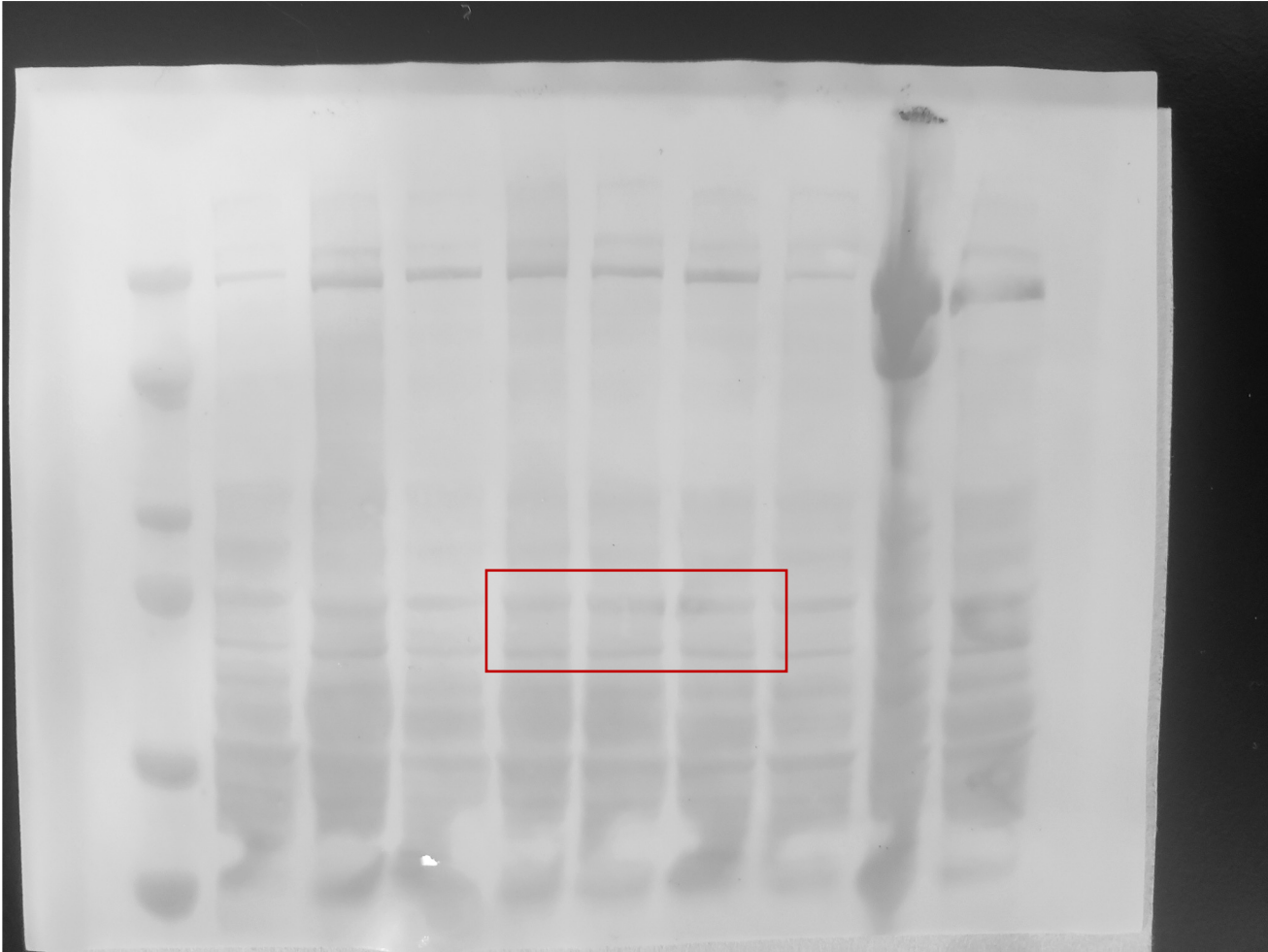

**Fig. 1I**

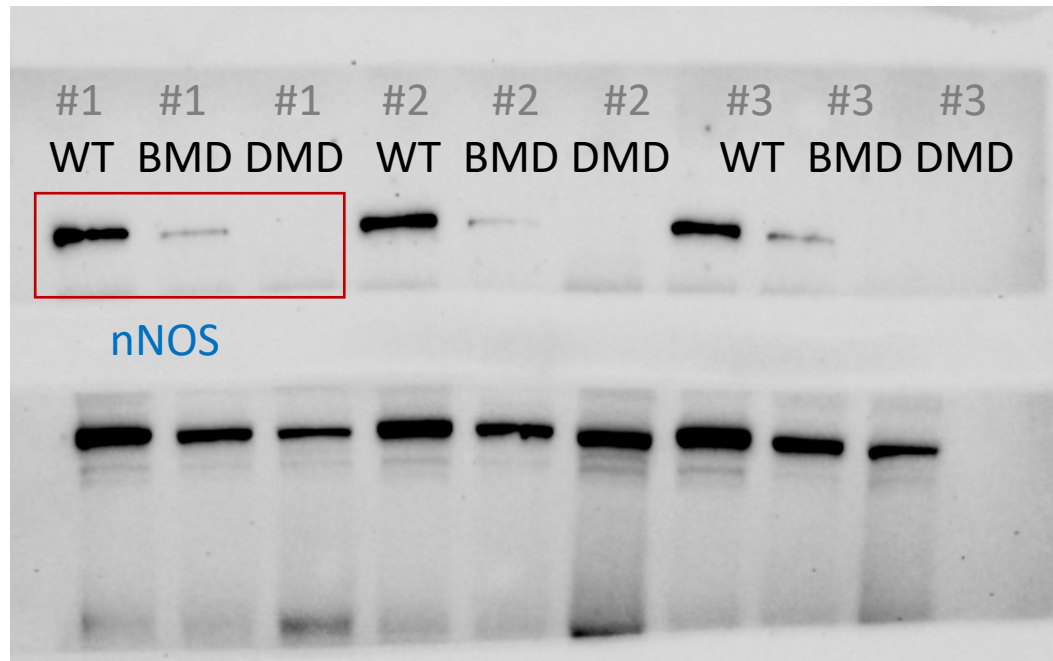

**Ponceau**

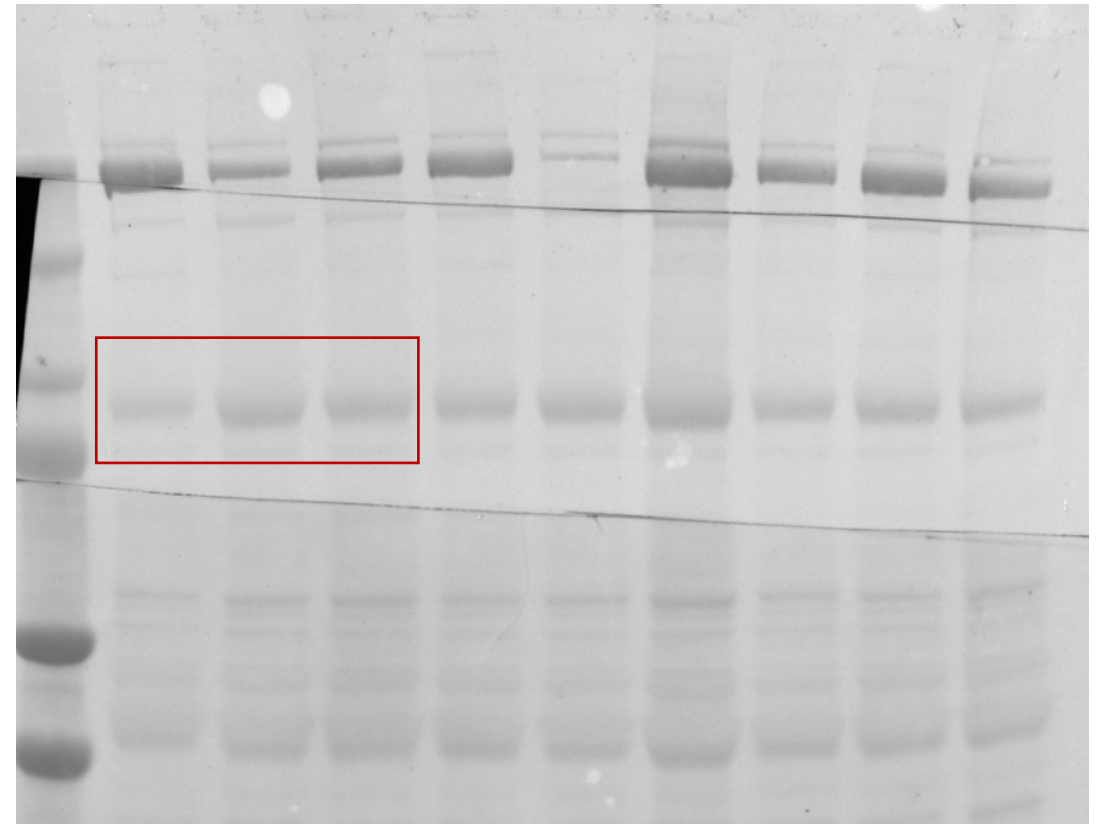

Supplement: Supplementary file 2 — Source data Fig. 1 [file 44319_2024_249_MOESM2_ESM.zip › FIG1/FIG.1B HEART.pdf]

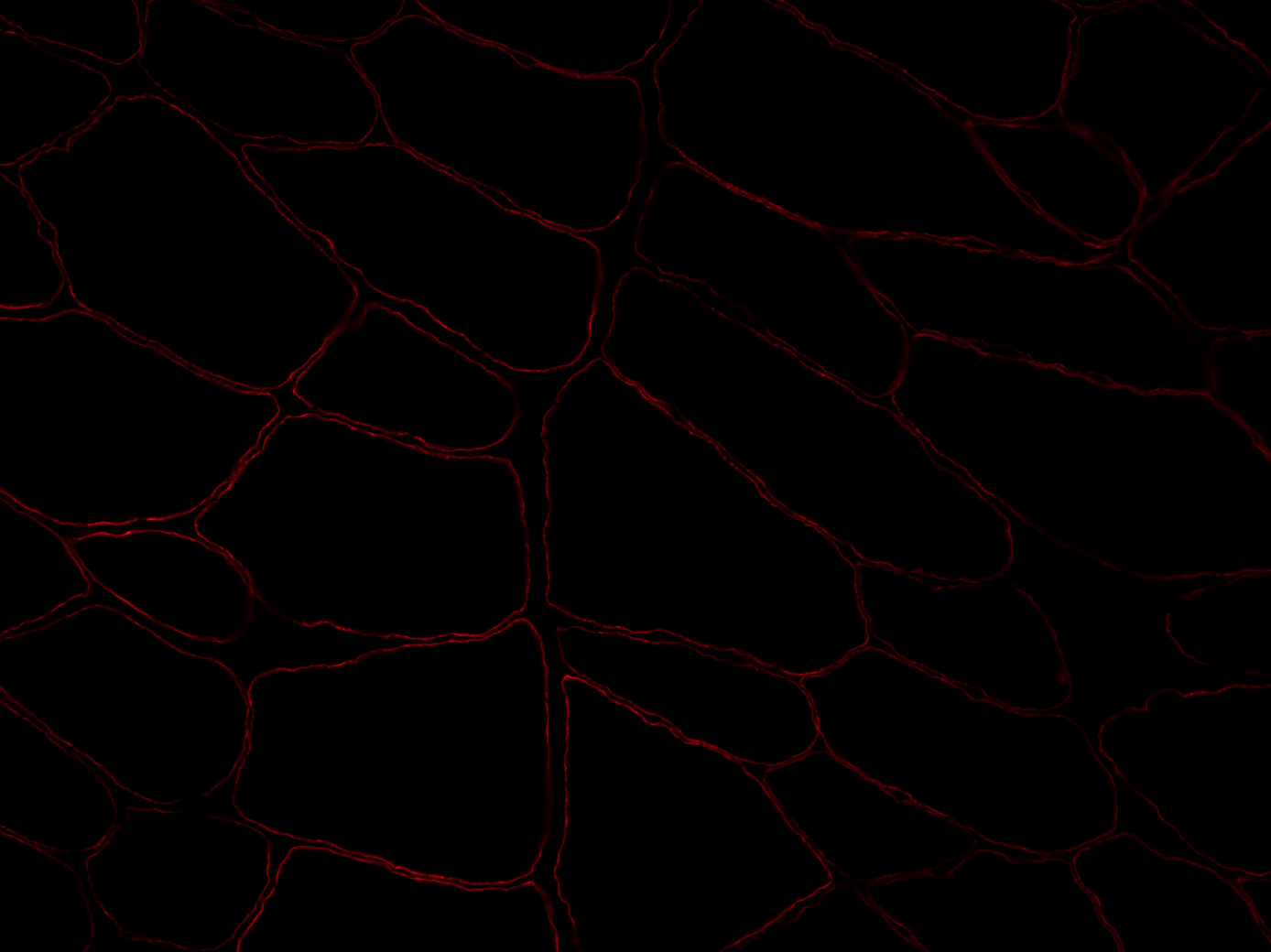

Supplement: Supplementary file 2 — Source data Fig. 1 [file 44319_2024_249_MOESM2_ESM.zip › FIG1/FIG.1E/TA BMD 745 3_c2.tif]

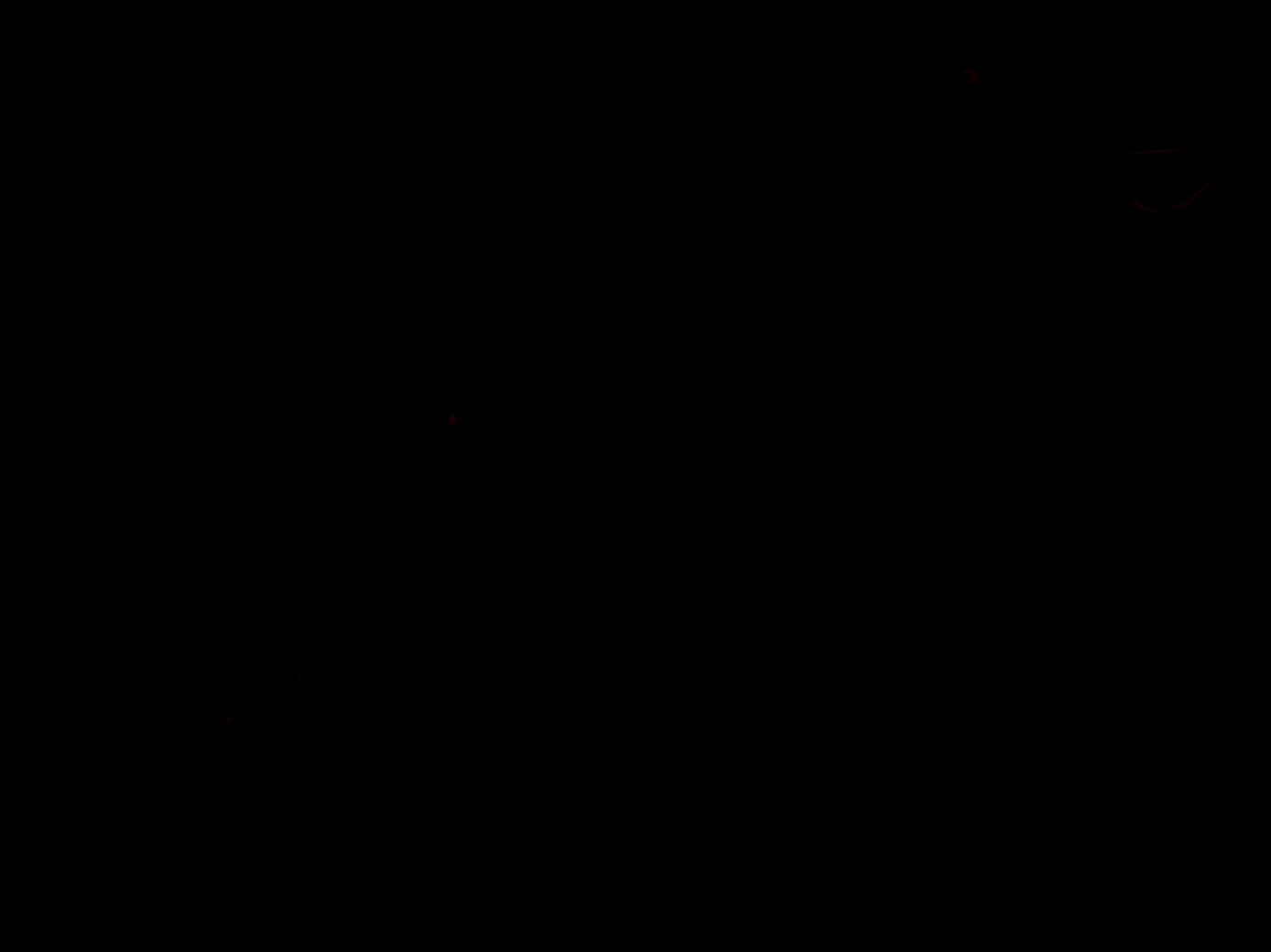

Supplement: Supplementary file 2 — Source data Fig. 1 [file 44319_2024_249_MOESM2_ESM.zip › FIG1/FIG.1E/TA DMD 239 5_c2.tif]

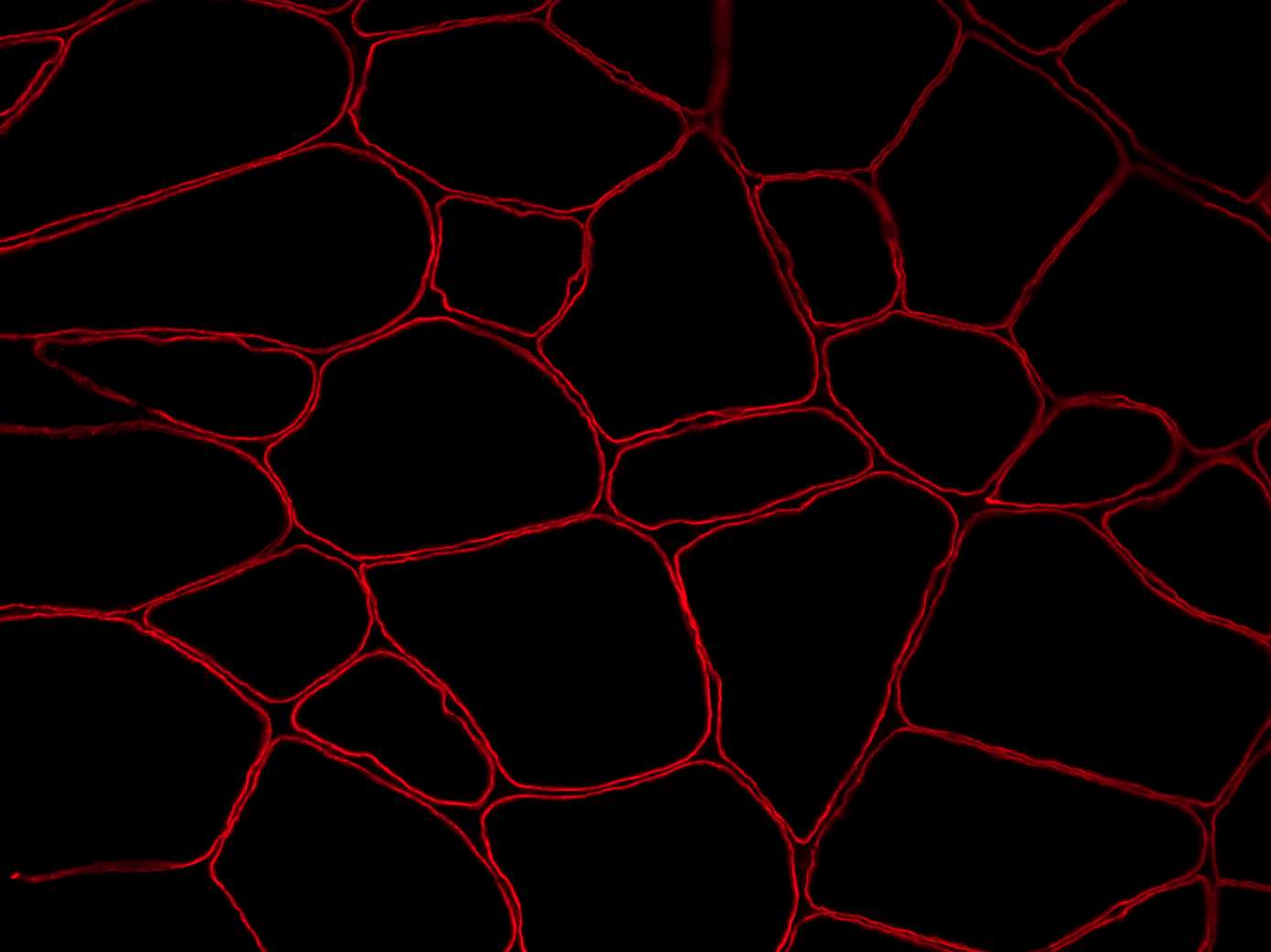

Supplement: Supplementary file 2 — Source data Fig. 1 [file 44319_2024_249_MOESM2_ESM.zip › FIG1/FIG.1E/TA WT 563 3_c2.tif]

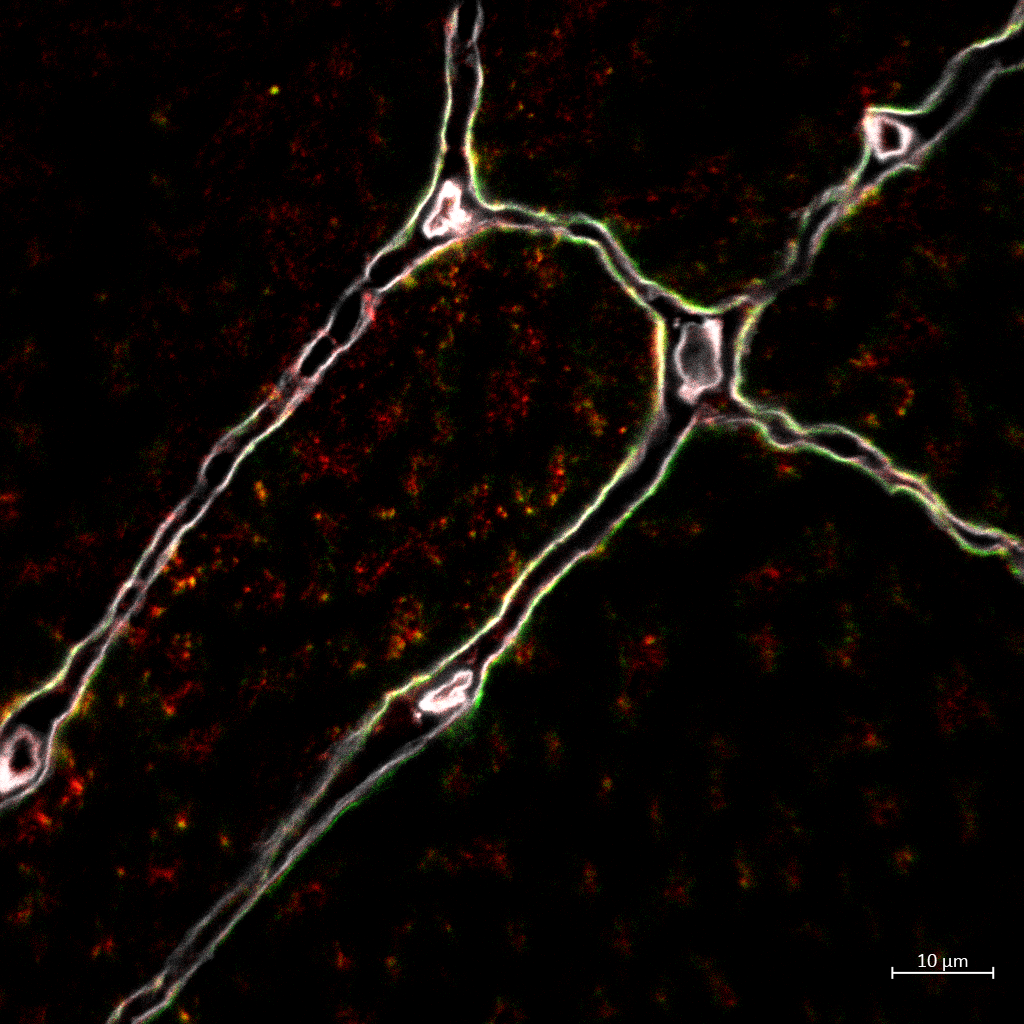

Supplement: Supplementary file 2 — Source data Fig. 1 [file 44319_2024_249_MOESM2_ESM.zip › FIG1/FIG.1G/BMD /BMD 2.tif]

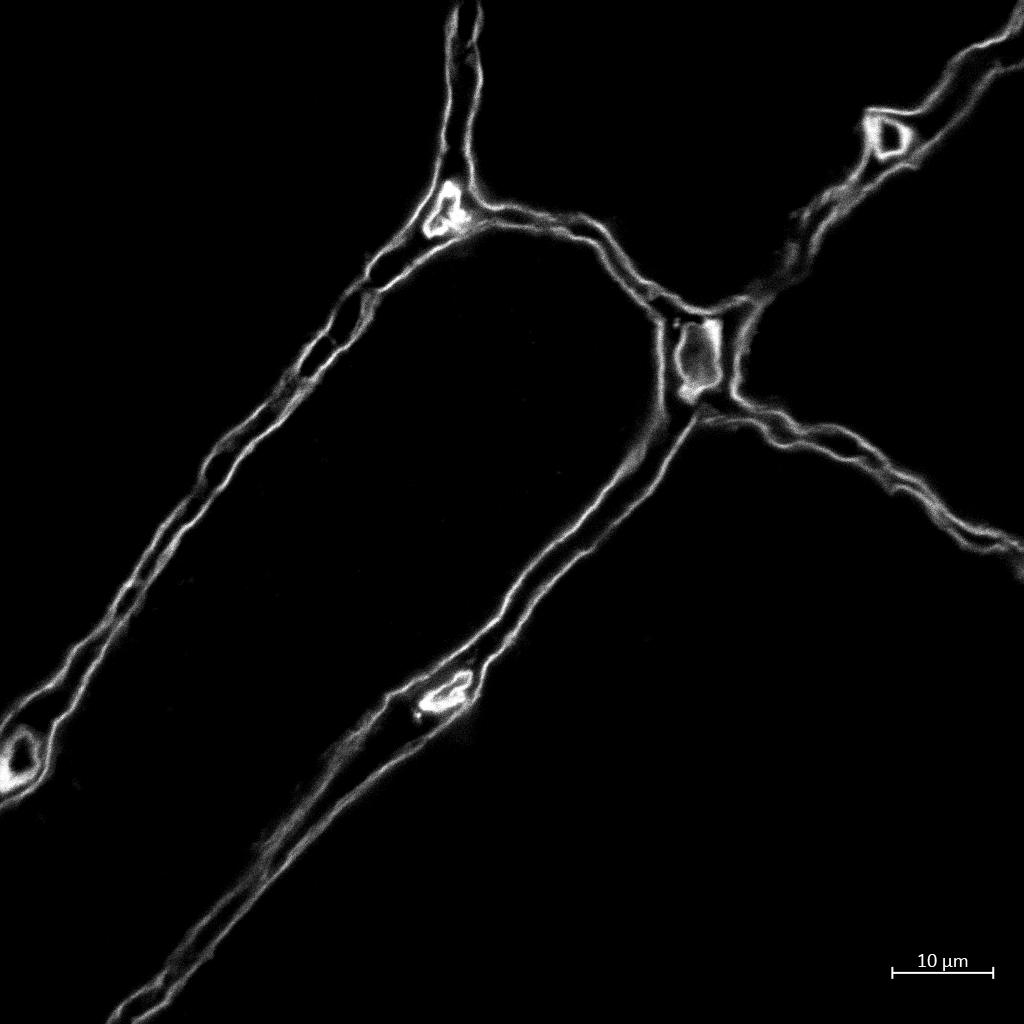

Supplement: Supplementary file 2 — Source data Fig. 1 [file 44319_2024_249_MOESM2_ESM.zip › FIG1/FIG.1G/BMD /BMD 2_Cy5-T1.tif]

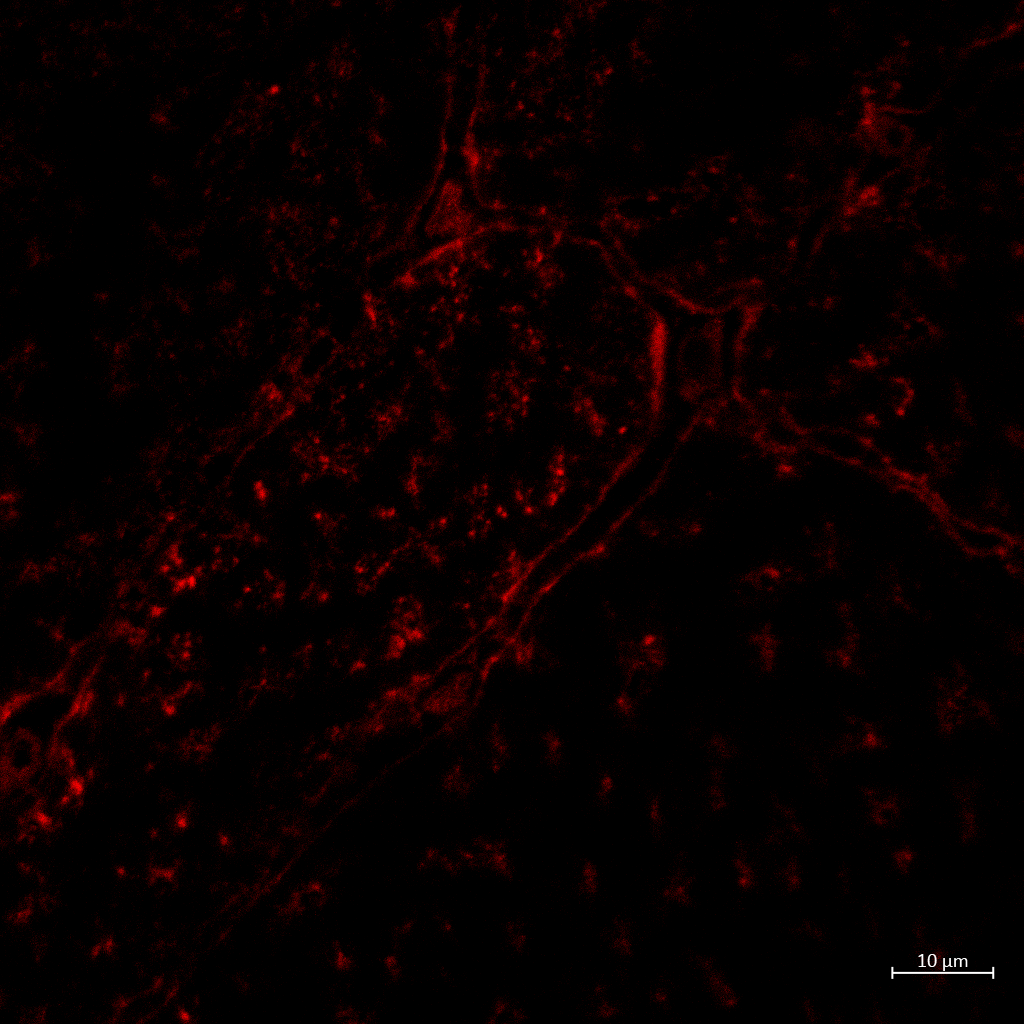

Supplement: Supplementary file 2 — Source data Fig. 1 [file 44319_2024_249_MOESM2_ESM.zip › FIG1/FIG.1G/BMD /BMD 2_DsRed-T2.tif]

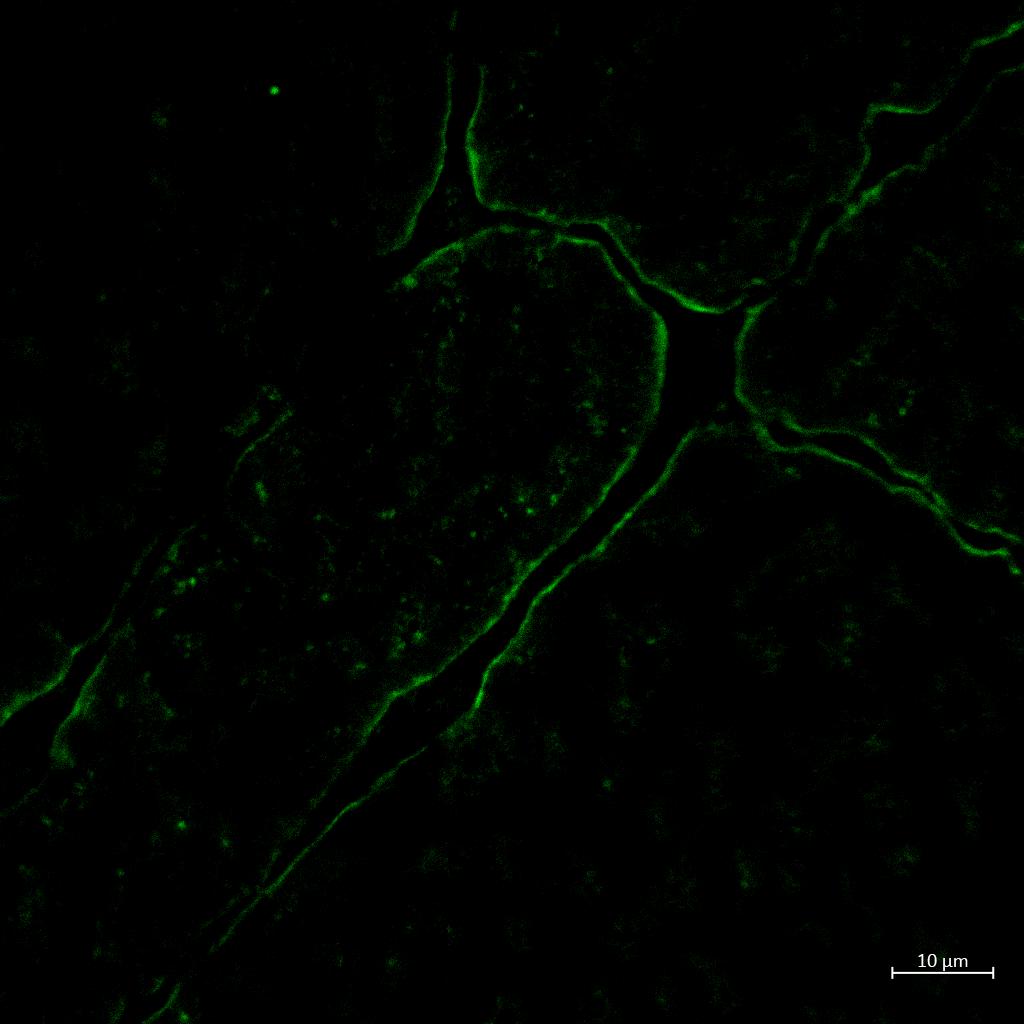

Supplement: Supplementary file 2 — Source data Fig. 1 [file 44319_2024_249_MOESM2_ESM.zip › FIG1/FIG.1G/BMD /BMD 2_EGFP-T3.tif]

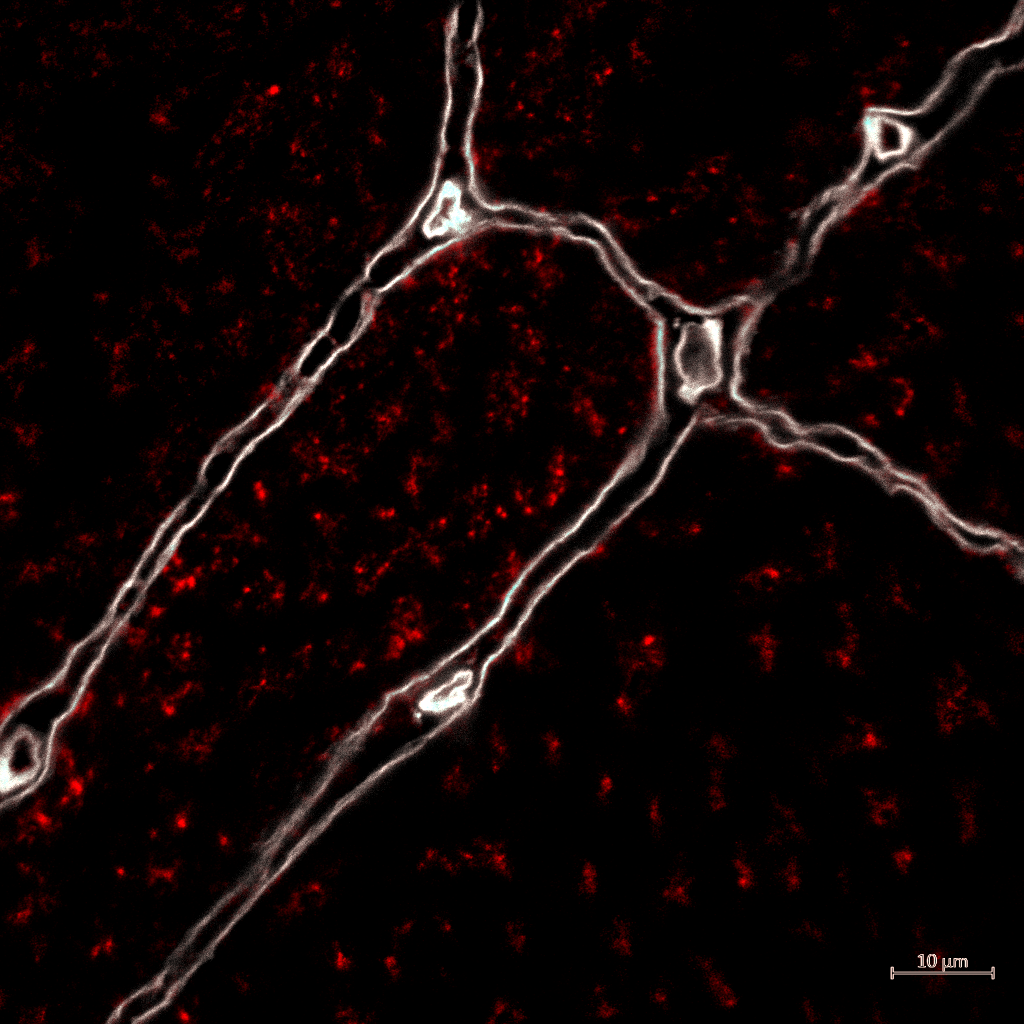

Supplement: Supplementary file 2 — Source data Fig. 1 [file 44319_2024_249_MOESM2_ESM.zip › FIG1/FIG.1G/BMD /OVERLAY.tiff]

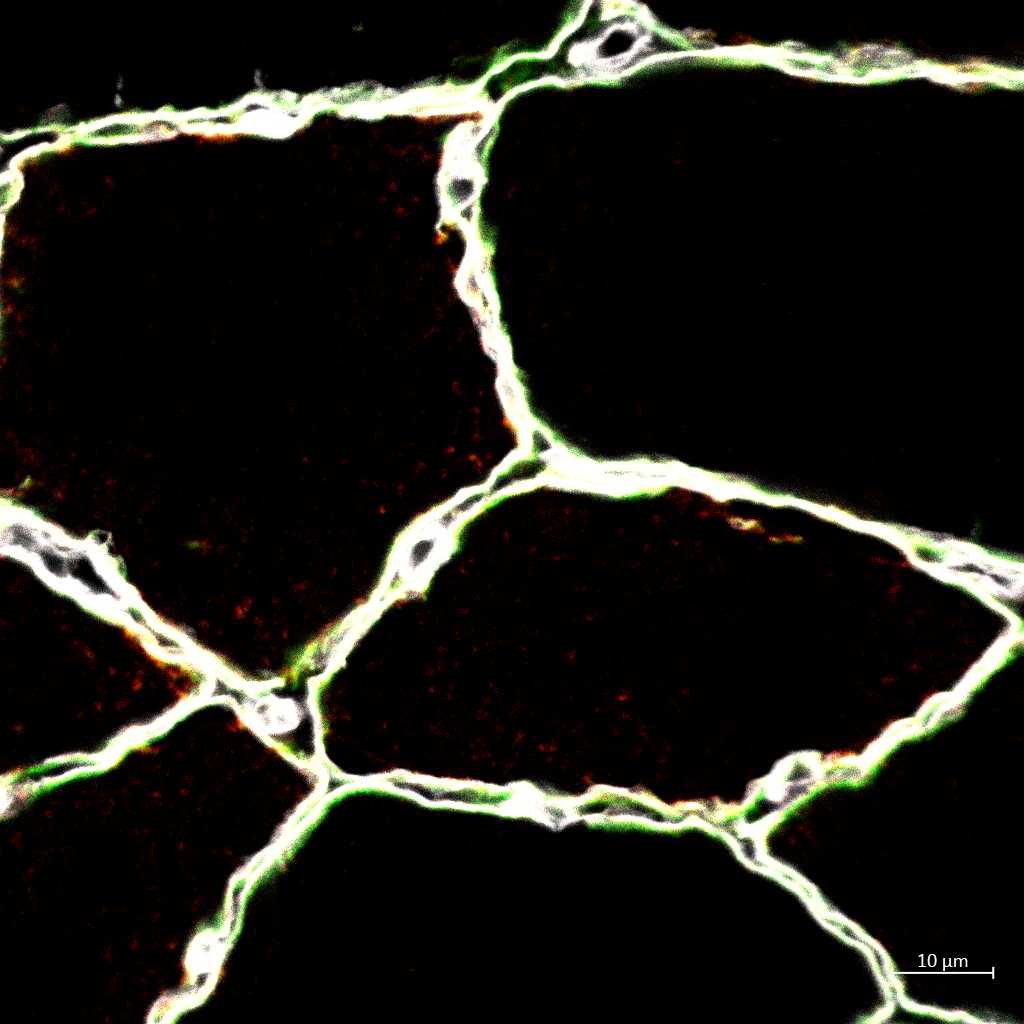

Supplement: Supplementary file 2 — Source data Fig. 1 [file 44319_2024_249_MOESM2_ESM.zip › FIG1/FIG.1G/WT/WT.tif]

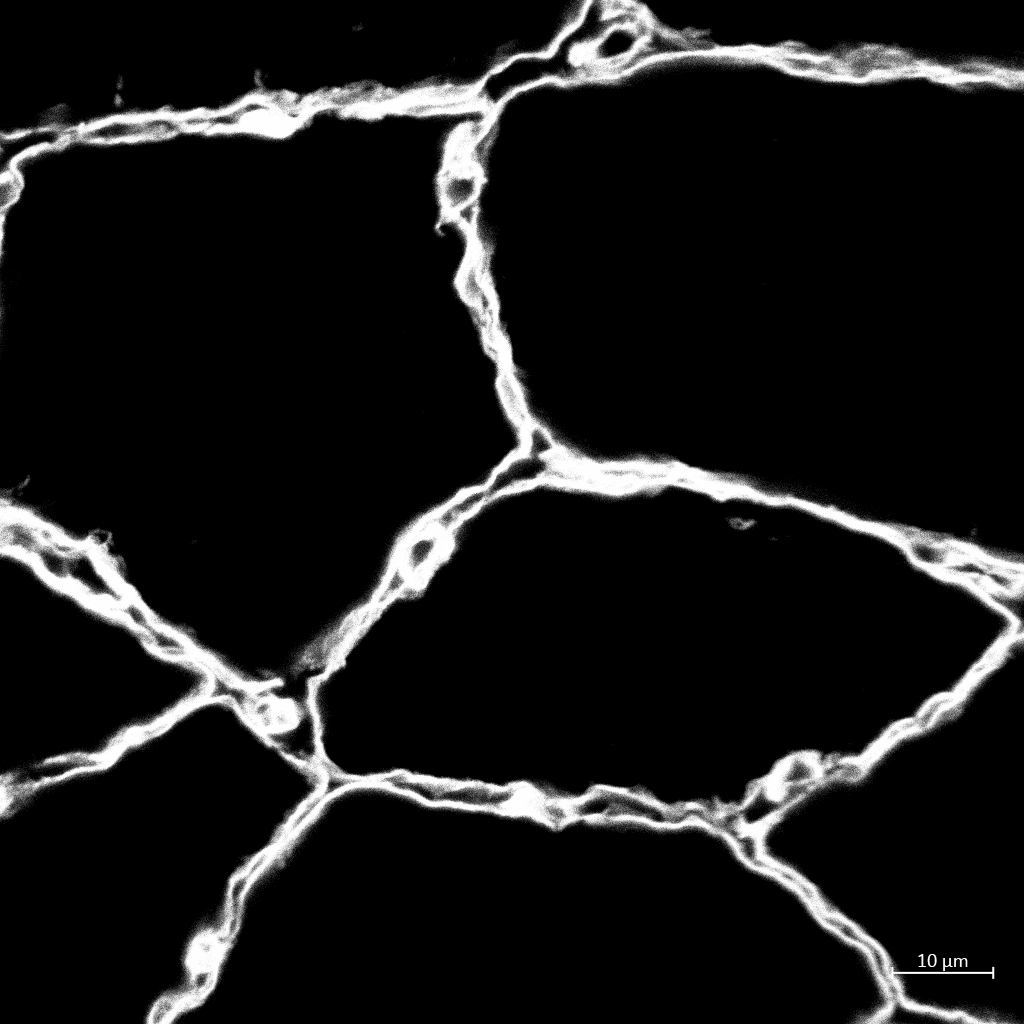

Supplement: Supplementary file 2 — Source data Fig. 1 [file 44319_2024_249_MOESM2_ESM.zip › FIG1/FIG.1G/WT/WT_Cy5-T1.tif]

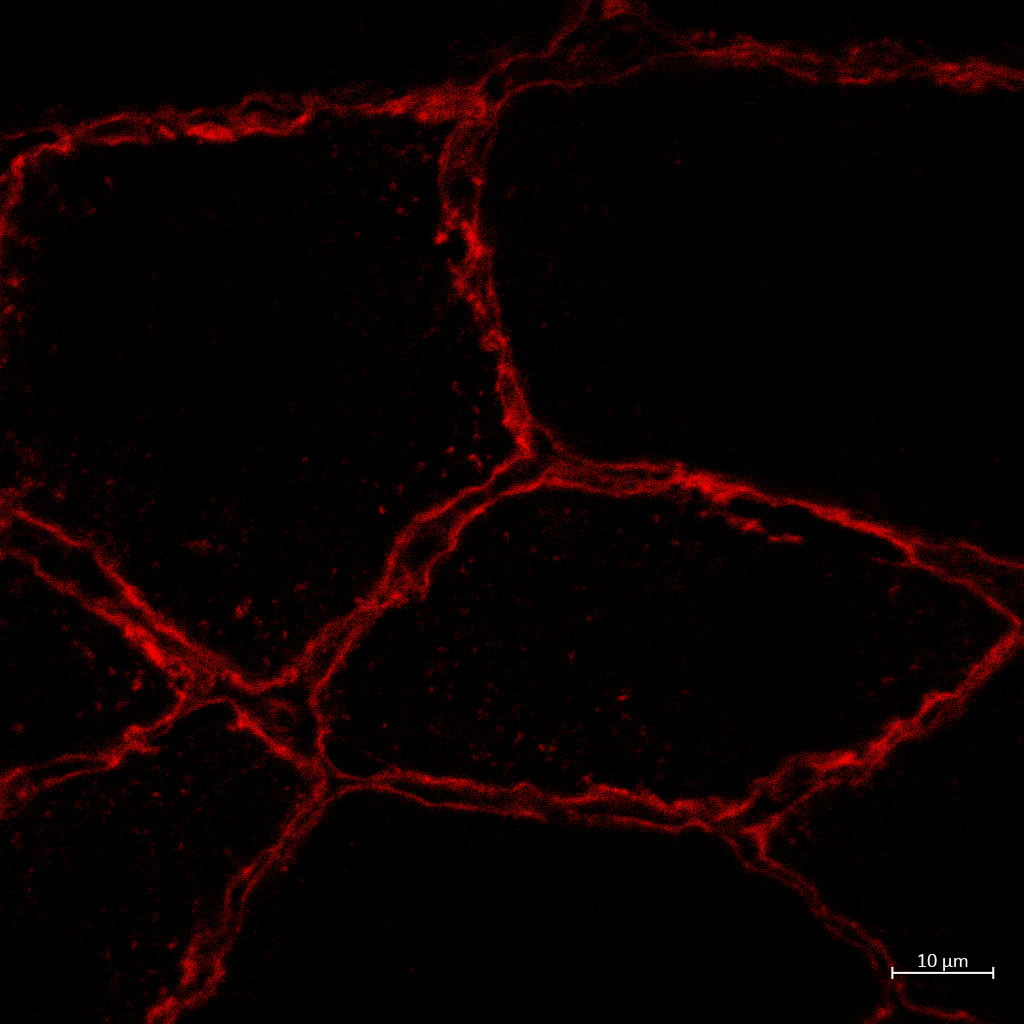

Supplement: Supplementary file 2 — Source data Fig. 1 [file 44319_2024_249_MOESM2_ESM.zip › FIG1/FIG.1G/WT/WT_DsRed-T2.tif]

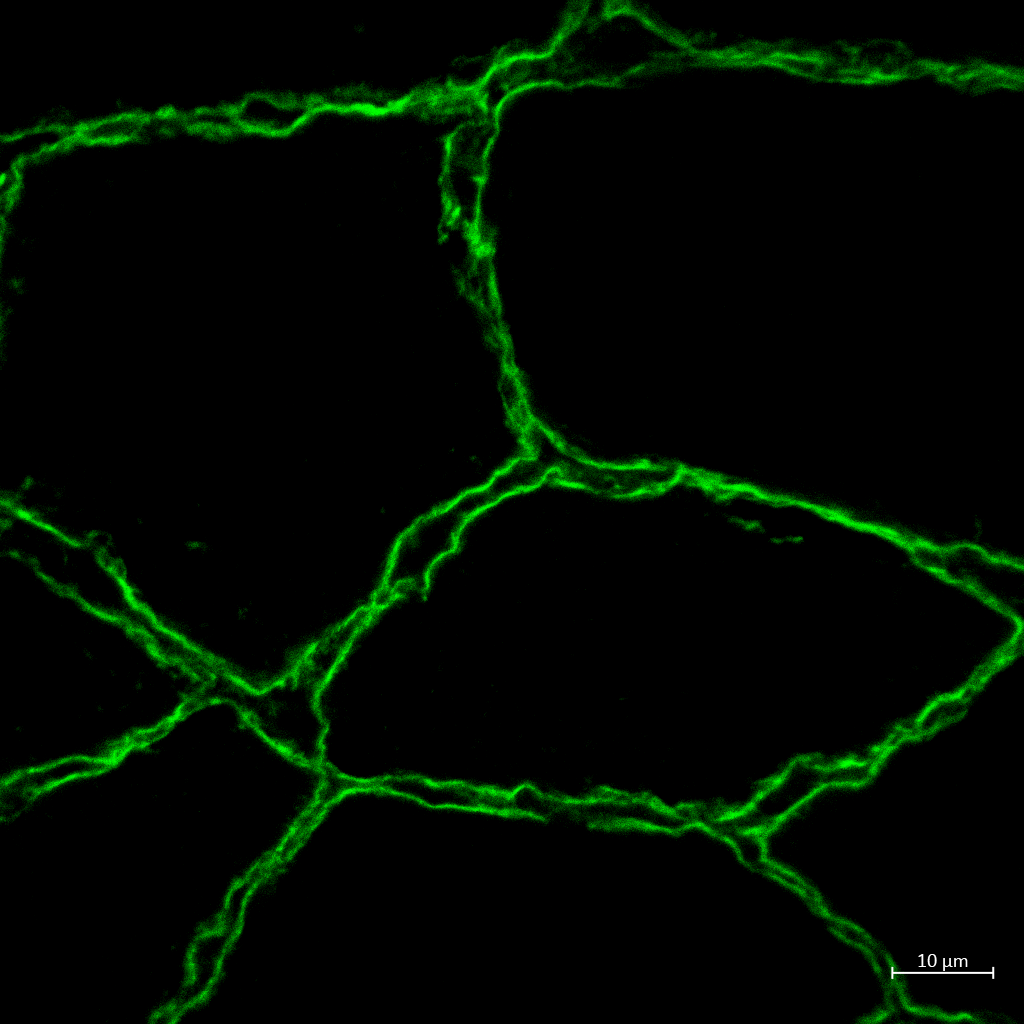

Supplement: Supplementary file 2 — Source data Fig. 1 [file 44319_2024_249_MOESM2_ESM.zip › FIG1/FIG.1G/WT/WT_EGFP-T3.tif]

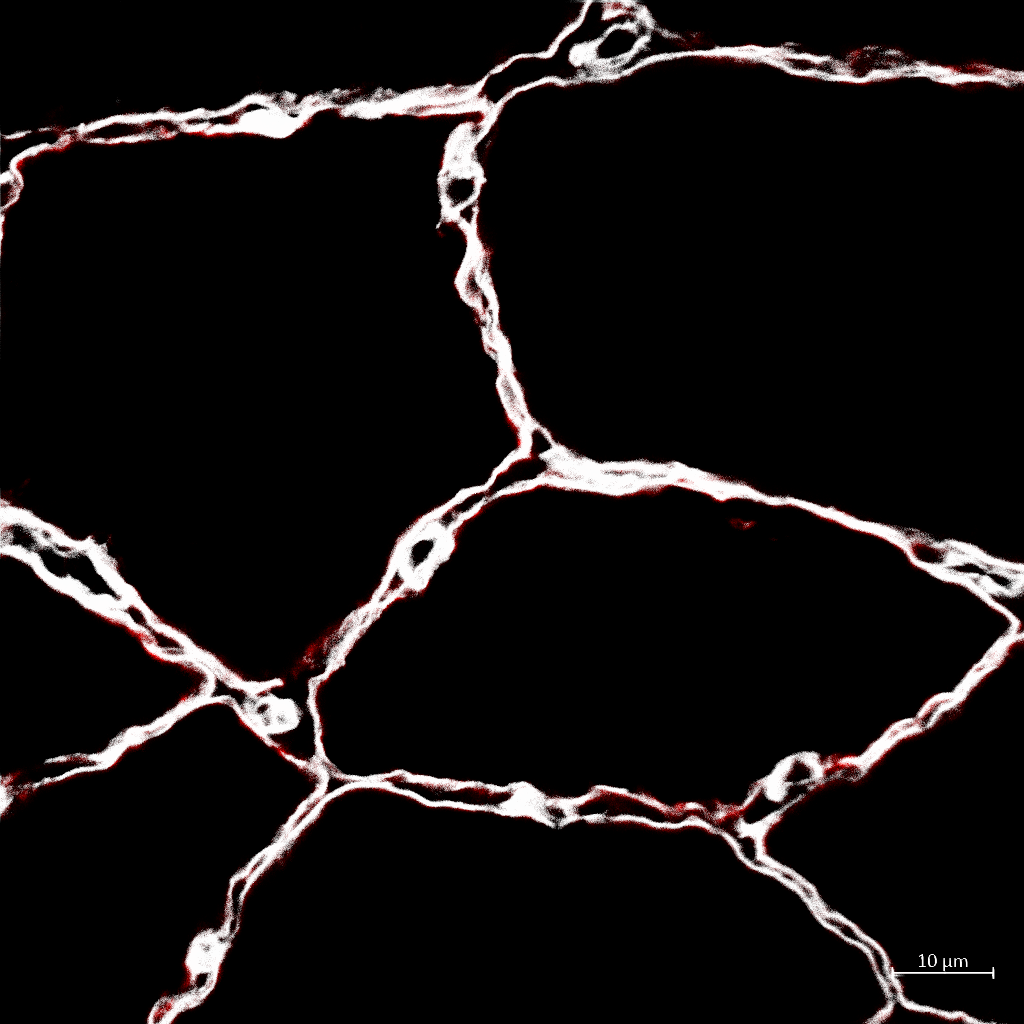

Supplement: Supplementary file 2 — Source data Fig. 1 [file 44319_2024_249_MOESM2_ESM.zip › FIG1/FIG.1G/WT/OVERLAY.tiff]

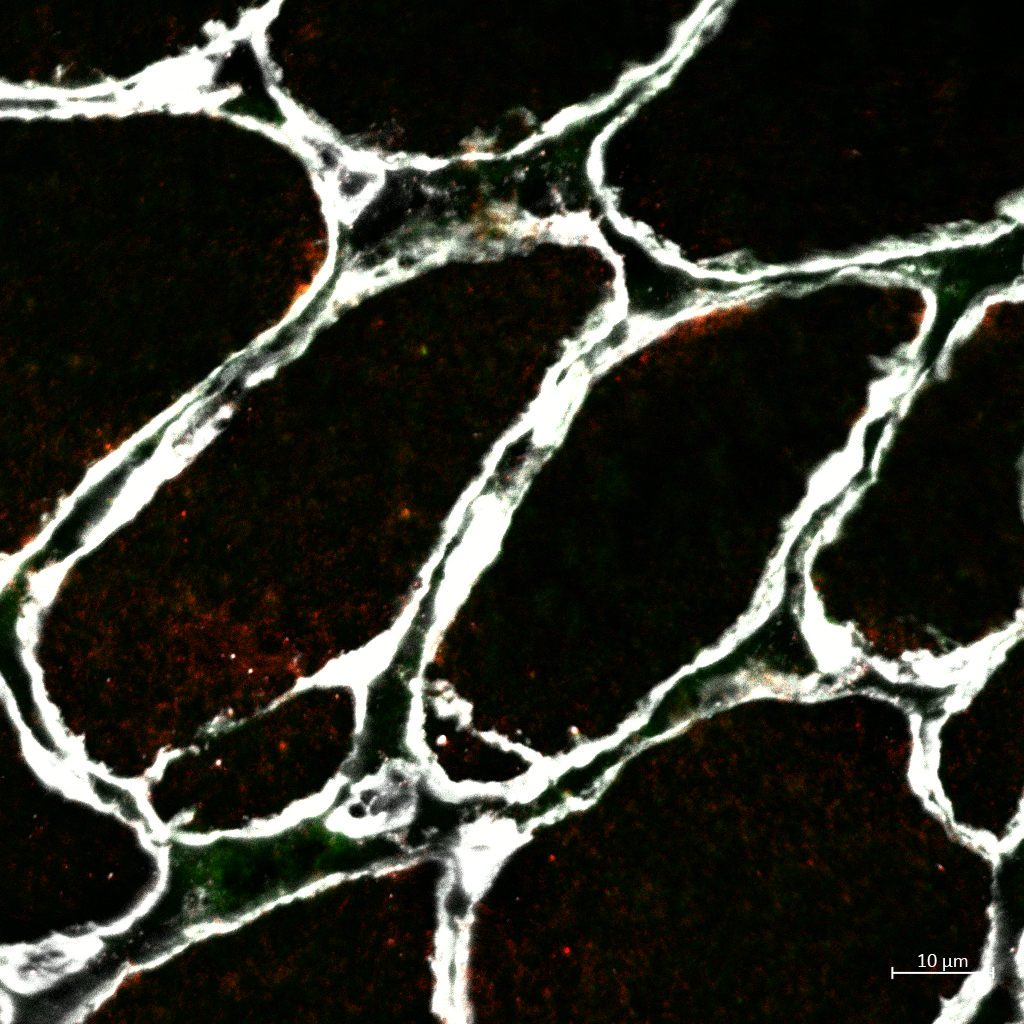

Supplement: Supplementary file 2 — Source data Fig. 1 [file 44319_2024_249_MOESM2_ESM.zip › FIG1/FIG.1G/DMD/DMD 6m 71.tif]

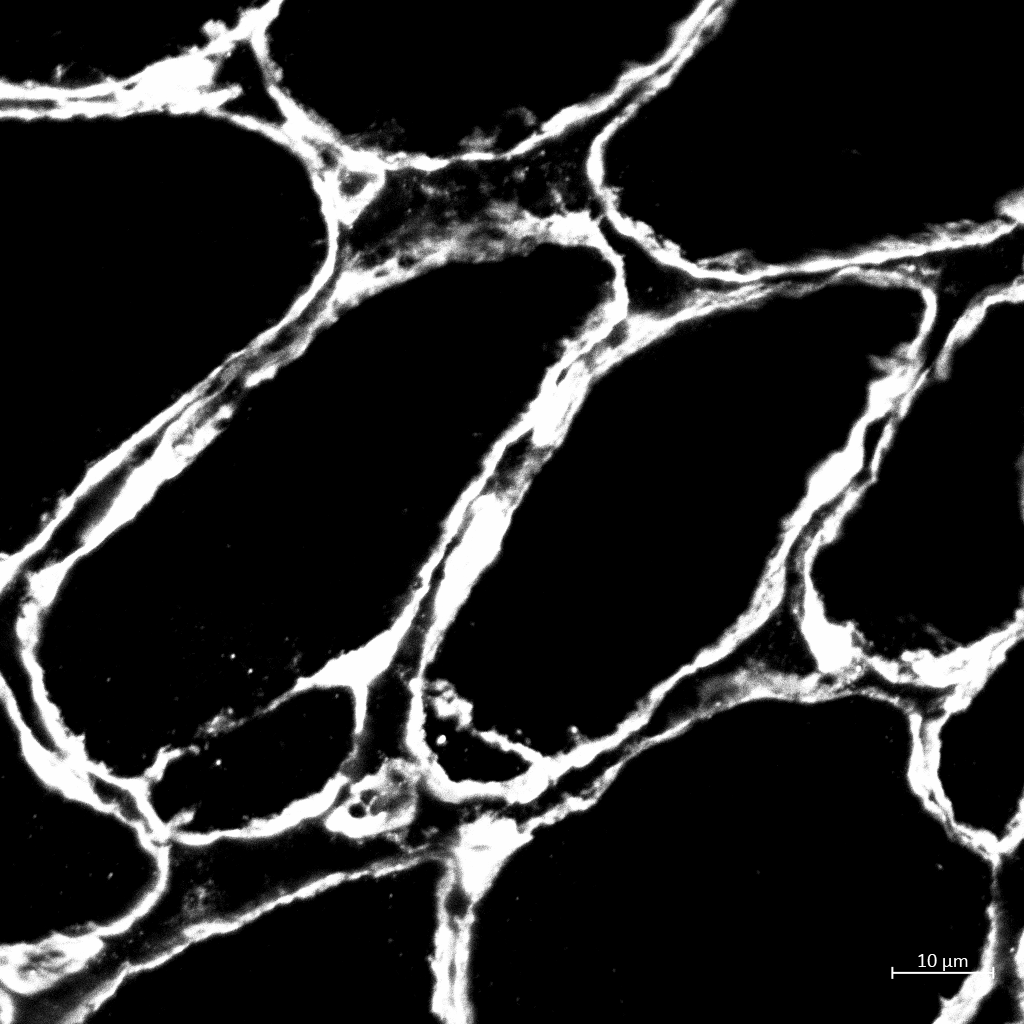

Supplement: Supplementary file 2 — Source data Fig. 1 [file 44319_2024_249_MOESM2_ESM.zip › FIG1/FIG.1G/DMD/DMD 6m 71_Cy5-T1.tif]

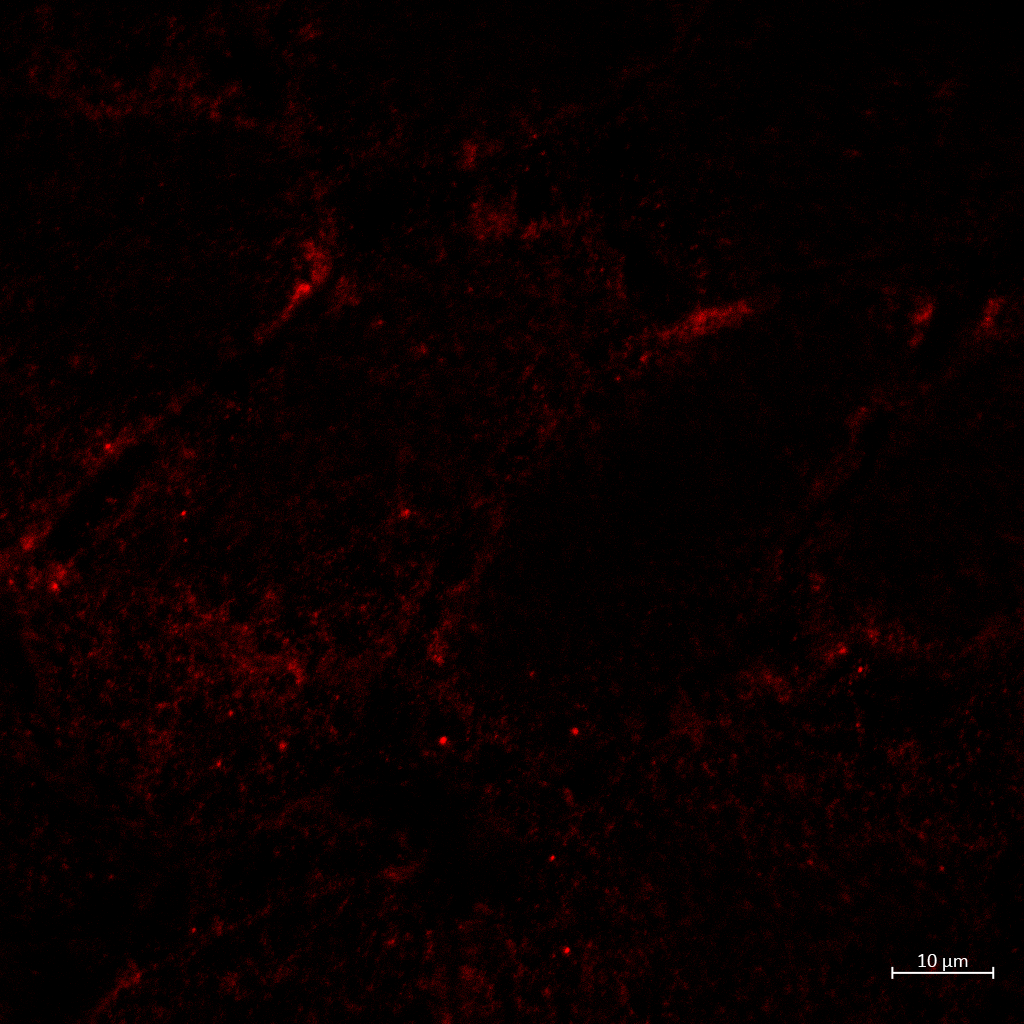

Supplement: Supplementary file 2 — Source data Fig. 1 [file 44319_2024_249_MOESM2_ESM.zip › FIG1/FIG.1G/DMD/DMD 6m 71_DsRed-T2.tif]

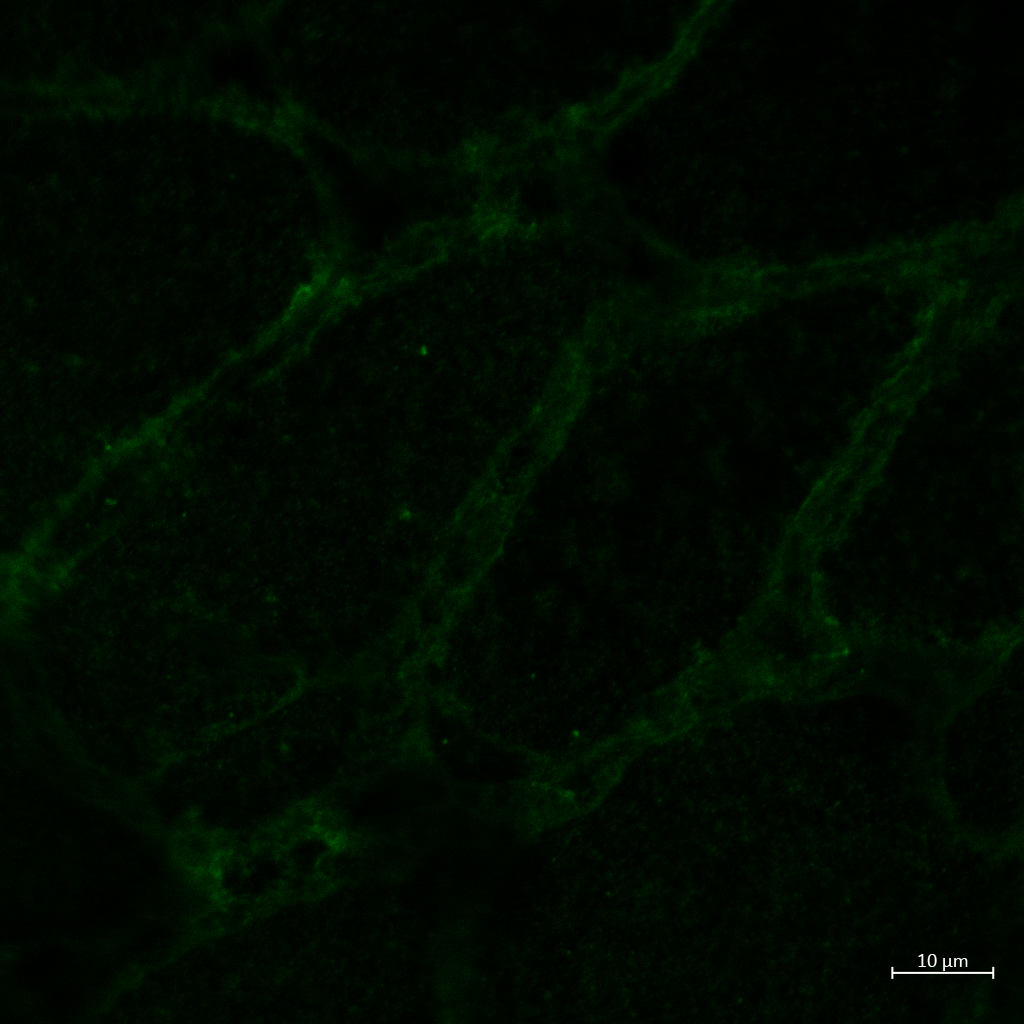

Supplement: Supplementary file 2 — Source data Fig. 1 [file 44319_2024_249_MOESM2_ESM.zip › FIG1/FIG.1G/DMD/DMD 6m 71_EGFP-T3.tif]

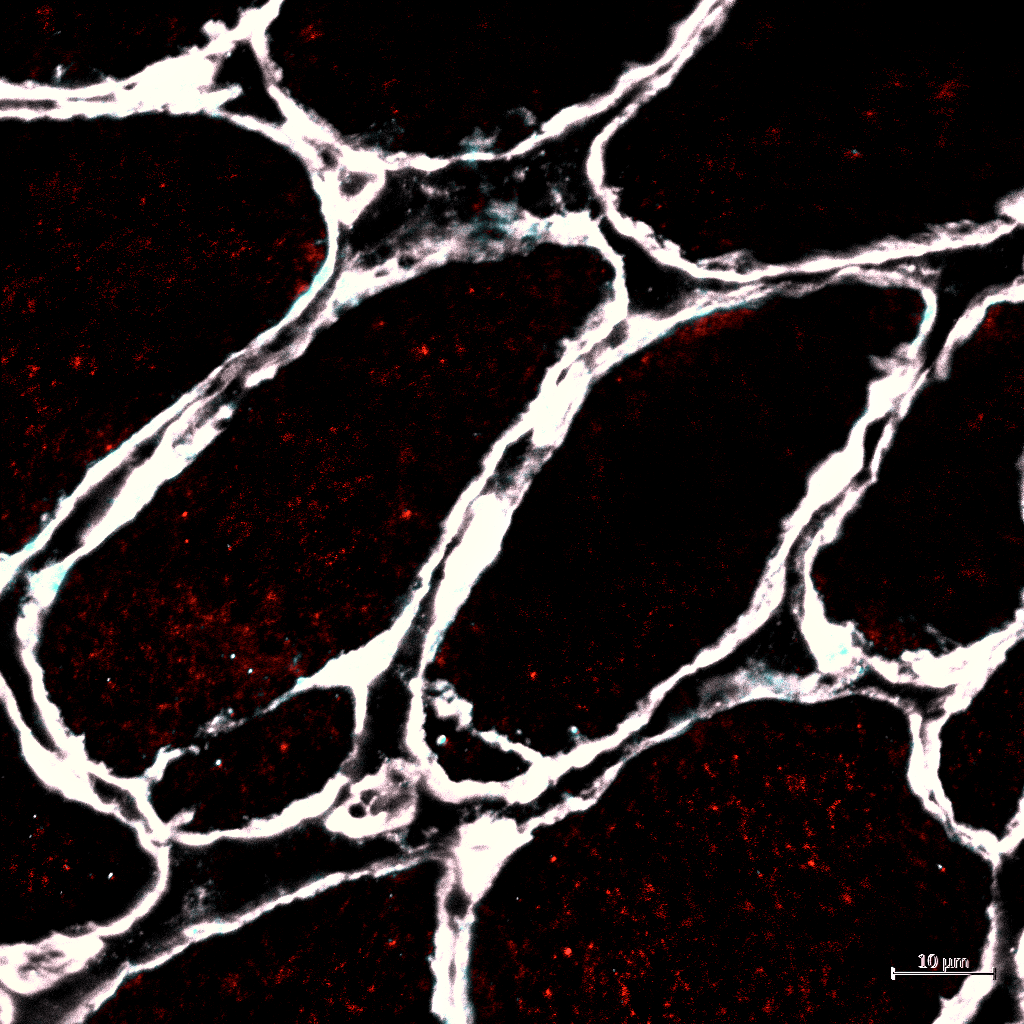

Supplement: Supplementary file 2 — Source data Fig. 1 [file 44319_2024_249_MOESM2_ESM.zip › FIG1/FIG.1G/DMD/OVERLAY.tiff]

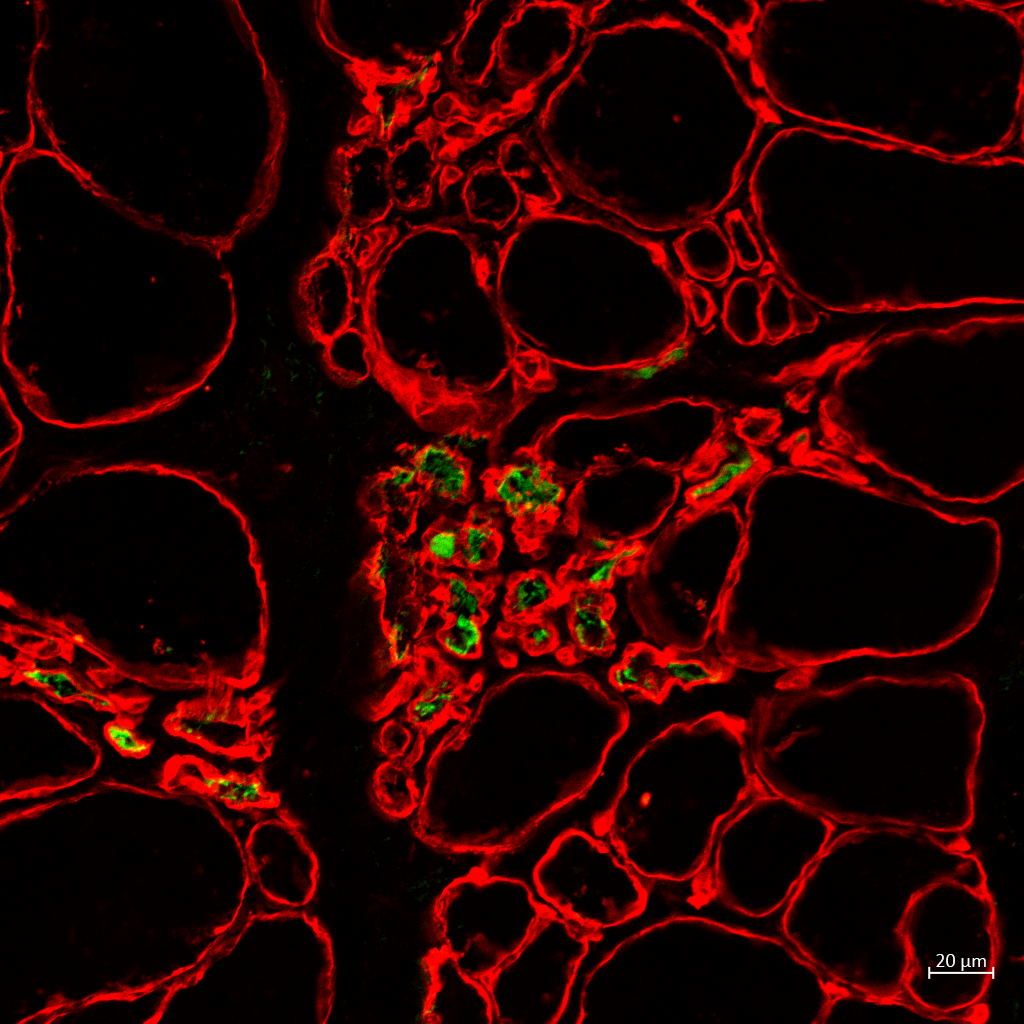

Supplement: Supplementary file 3 — Source data Fig. 2 [file 44319_2024_249_MOESM3_ESM.zip › FIG2/FIG.2G/DMD 3.tif]

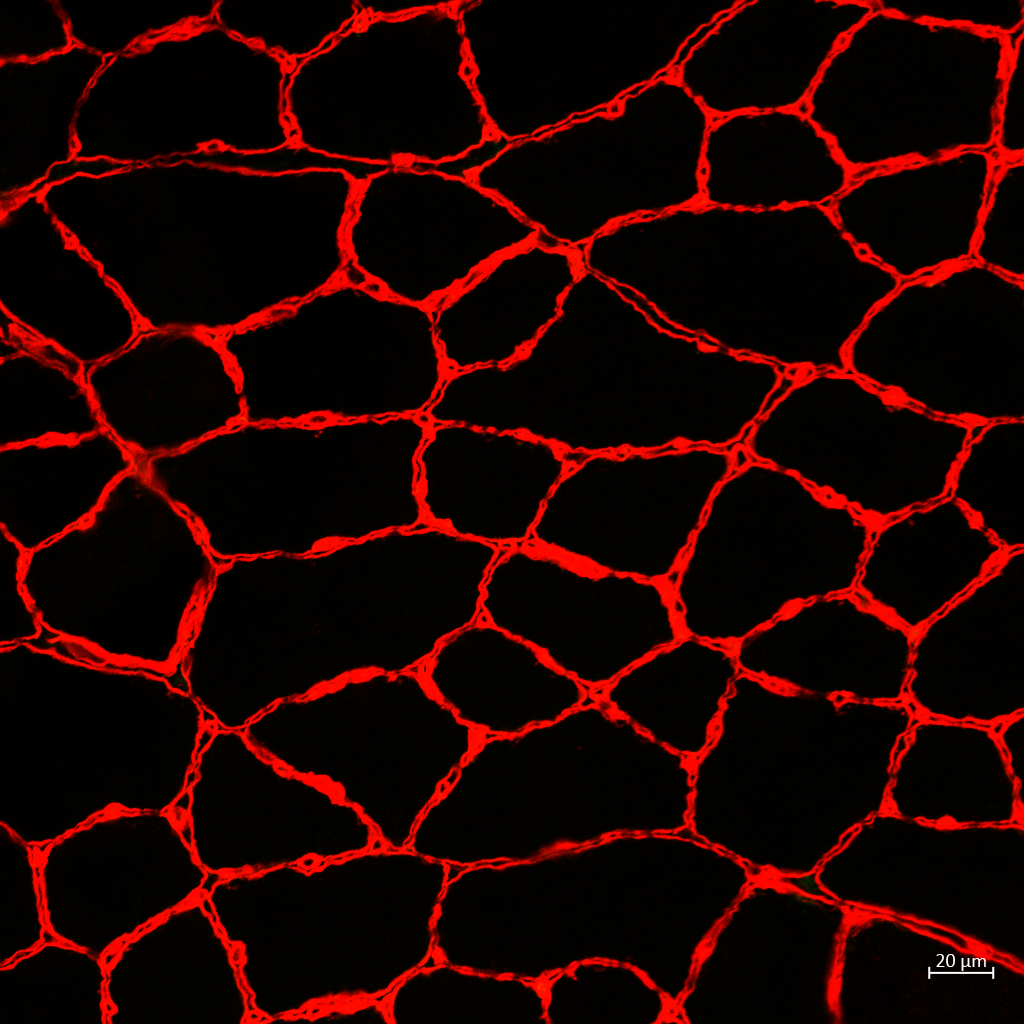

Supplement: Supplementary file 3 — Source data Fig. 2 [file 44319_2024_249_MOESM3_ESM.zip › FIG2/FIG.2G/WT.tif]

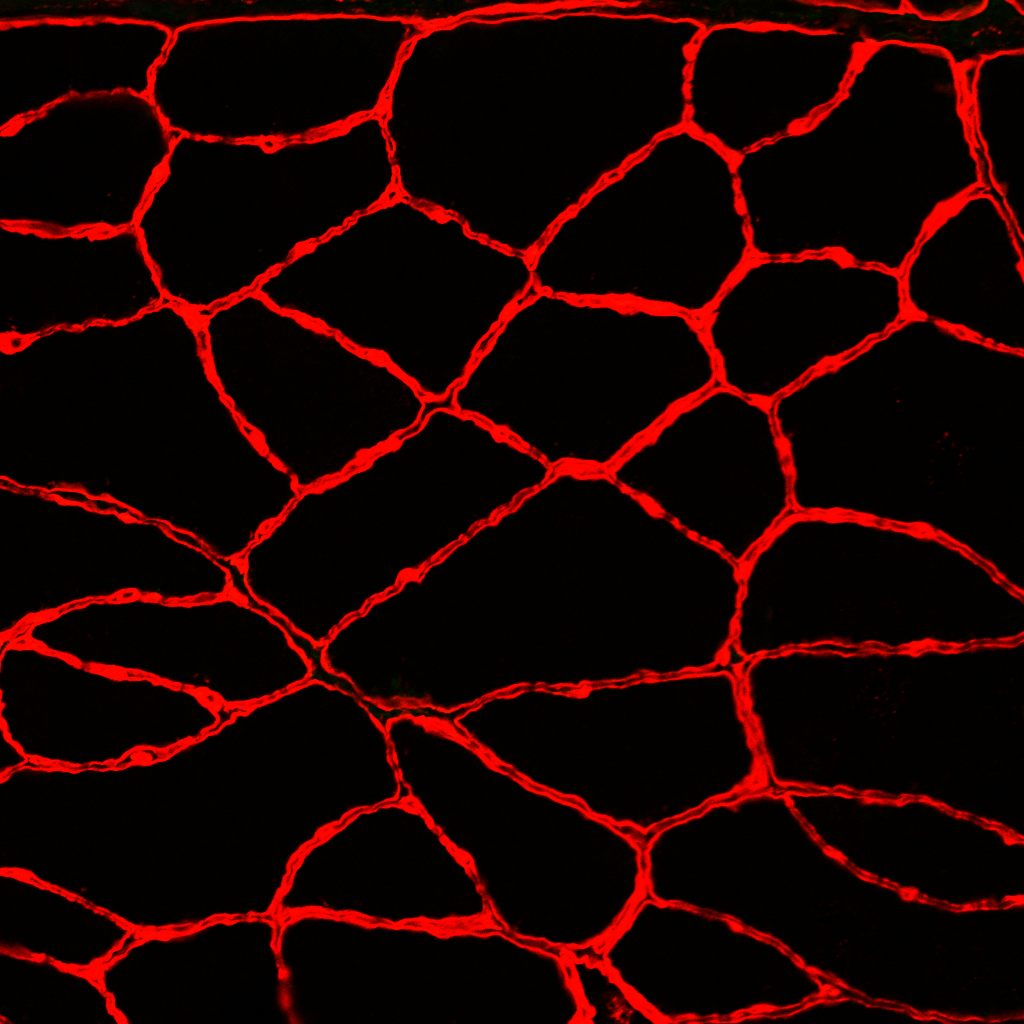

Supplement: Supplementary file 3 — Source data Fig. 2 [file 44319_2024_249_MOESM3_ESM.zip › FIG2/FIG.2G/BMD 4.tif]

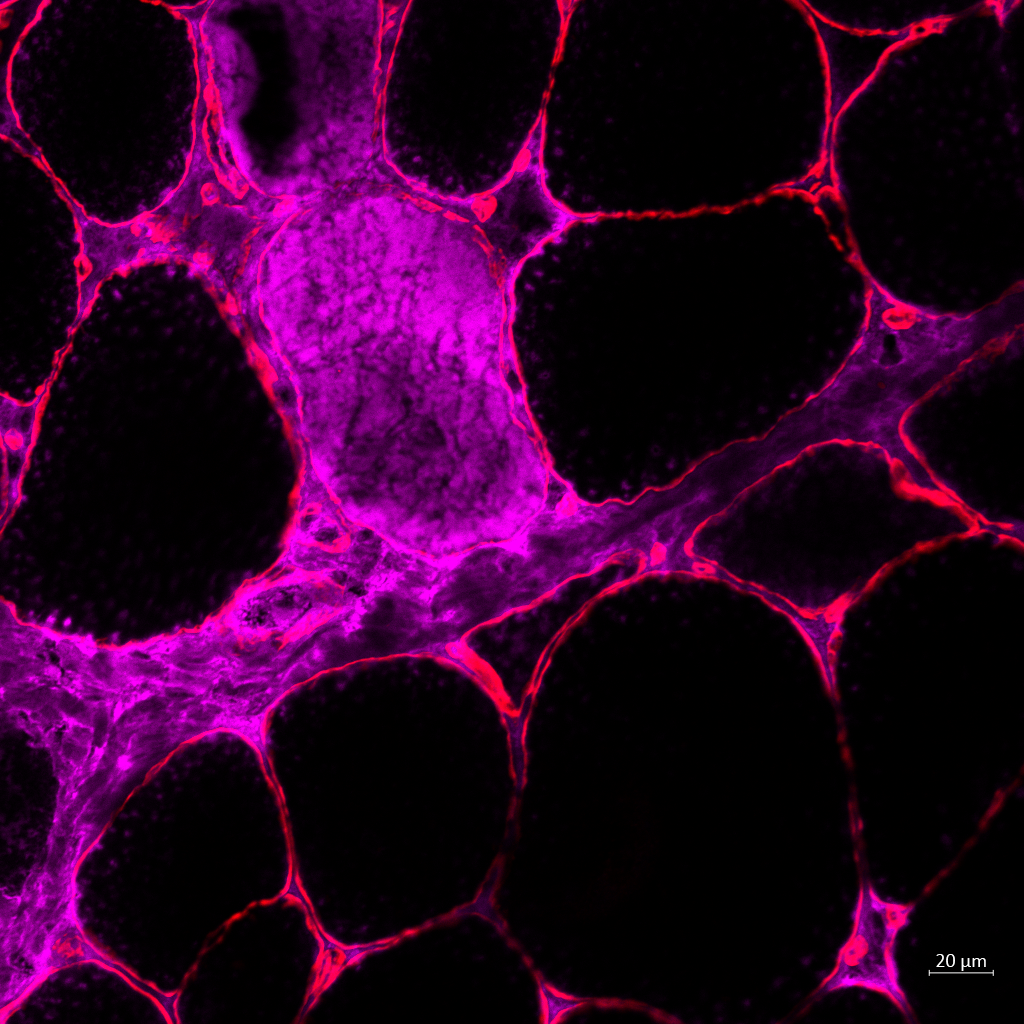

Supplement: Supplementary file 3 — Source data Fig. 2 [file 44319_2024_249_MOESM3_ESM.zip › FIG2/FIG.2E/dmd.tif]

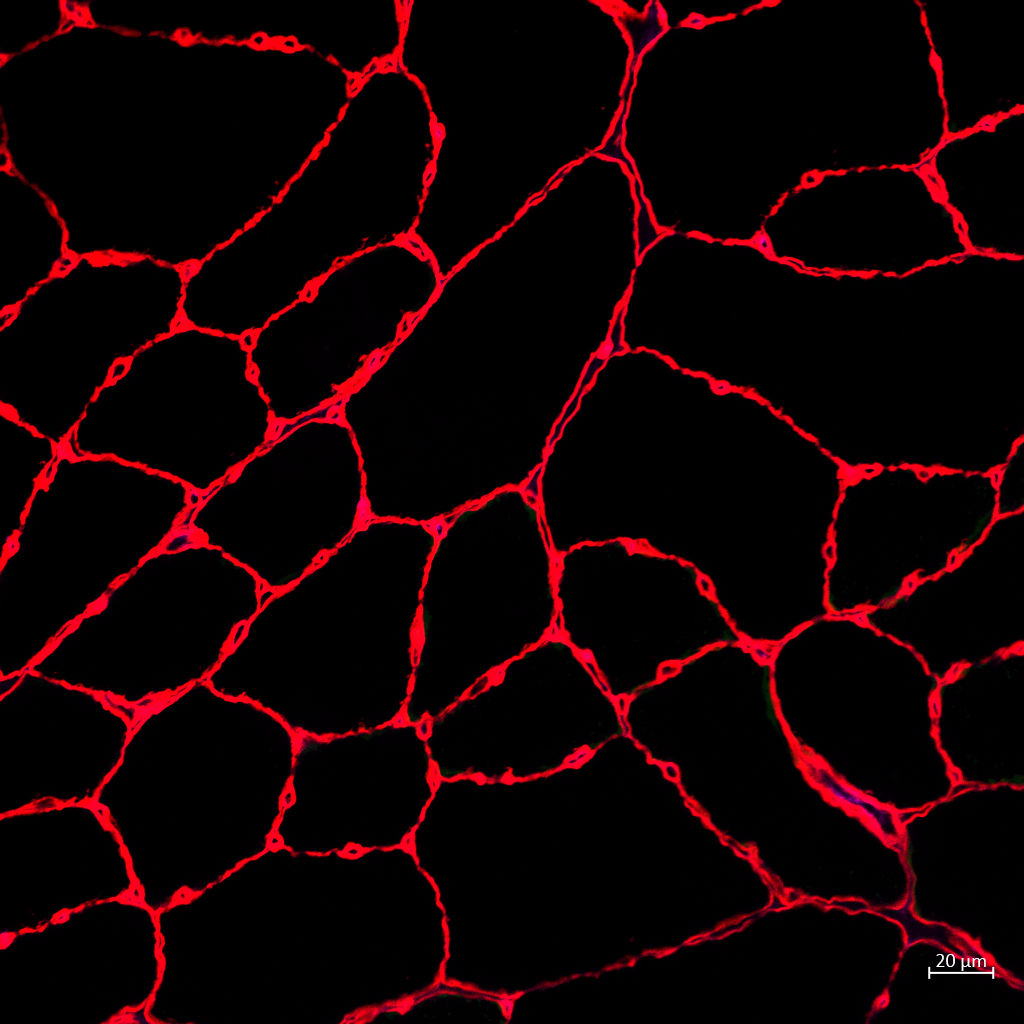

Supplement: Supplementary file 3 — Source data Fig. 2 [file 44319_2024_249_MOESM3_ESM.zip › FIG2/FIG.2E/wt 562.tif]

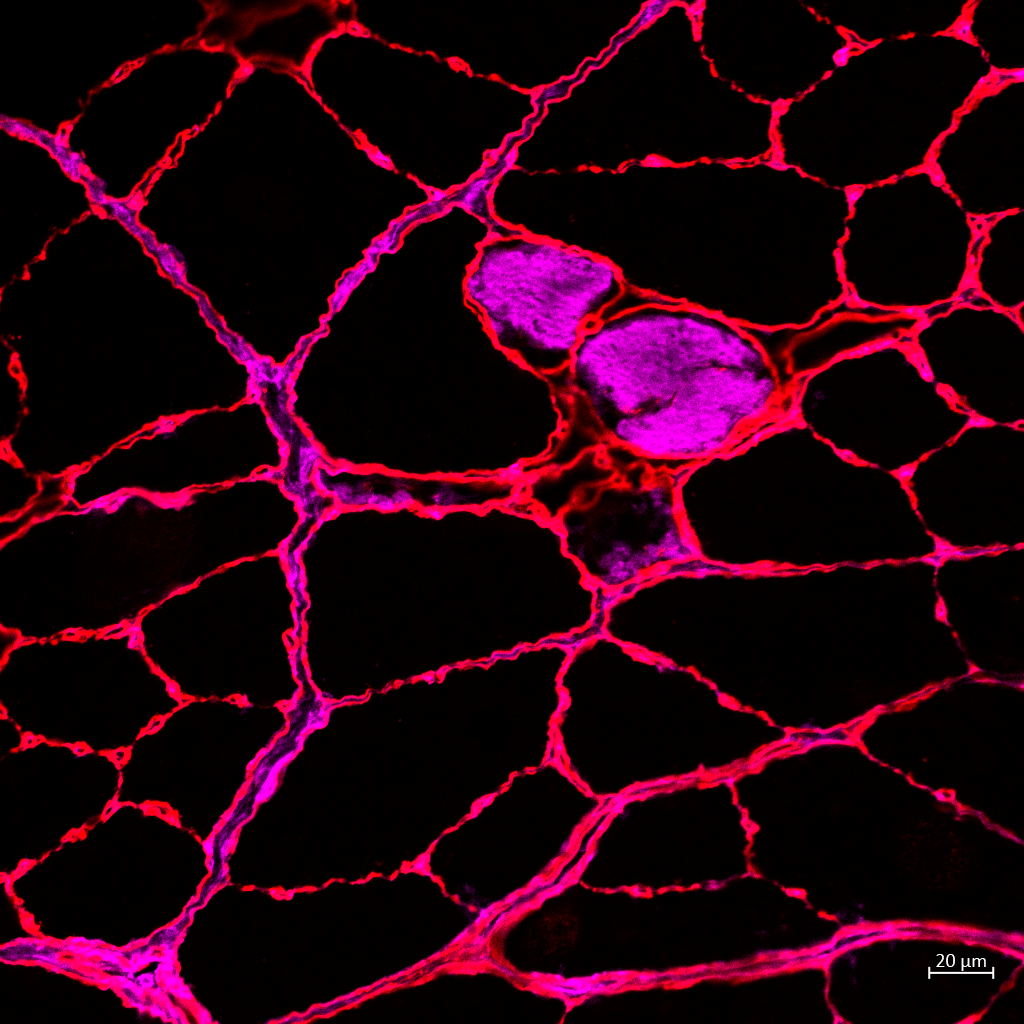

Supplement: Supplementary file 3 — Source data Fig. 2 [file 44319_2024_249_MOESM3_ESM.zip › FIG2/FIG.2E/BMD.tif]

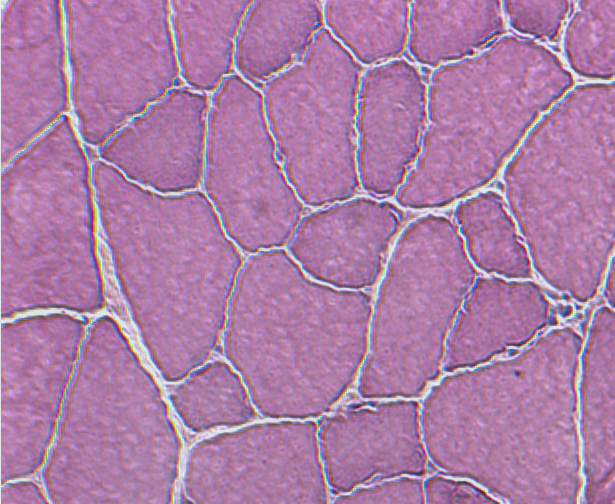

Supplement: Supplementary file 3 — Source data Fig. 2 [file 44319_2024_249_MOESM3_ESM.zip › FIG2/FIG.2A/HE BMD 12M.tiff]

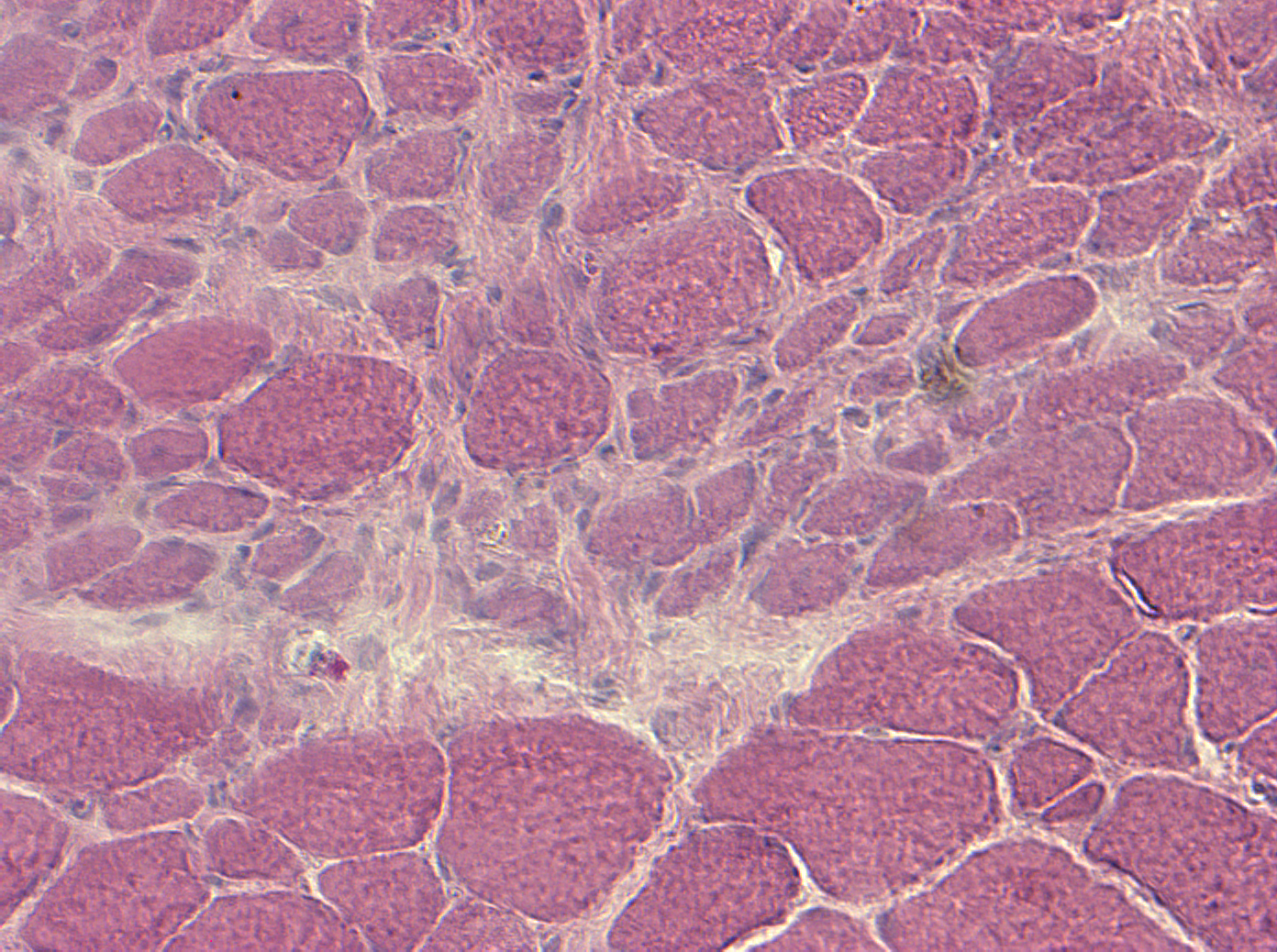

Supplement: Supplementary file 3 — Source data Fig. 2 [file 44319_2024_249_MOESM3_ESM.zip › FIG2/FIG.2A/HE TA DMD 6m.tif]

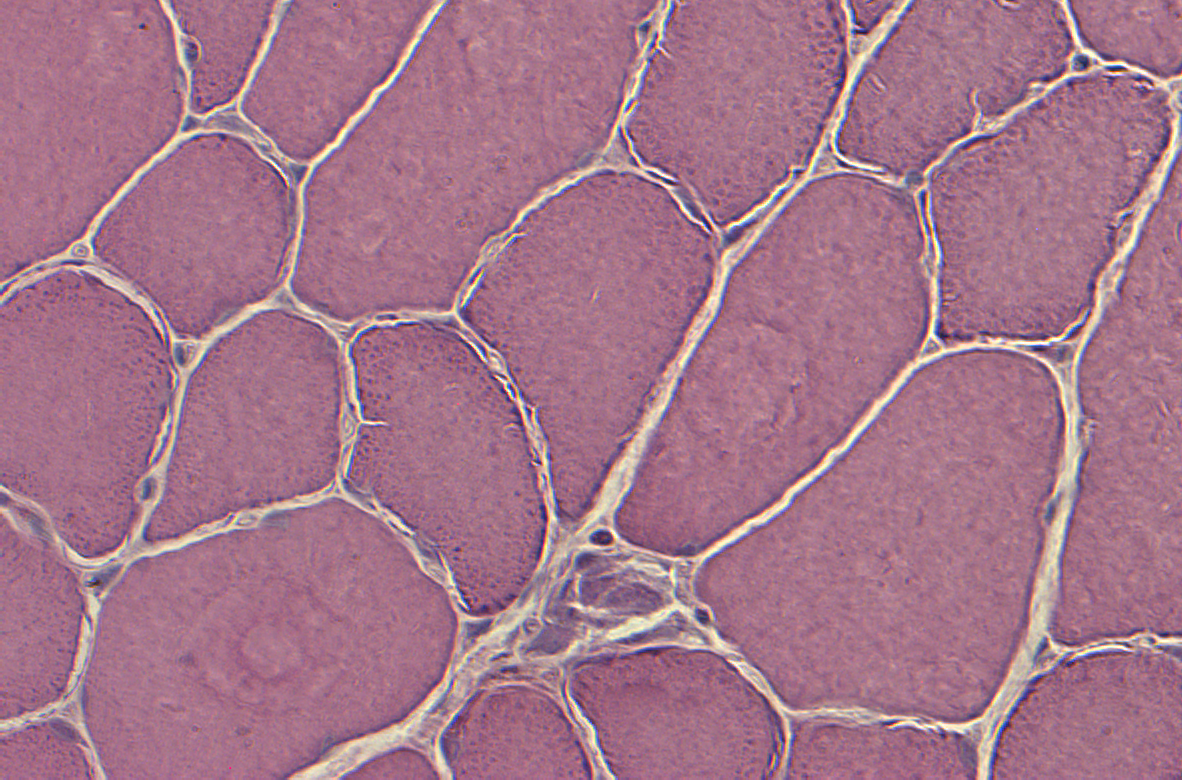

Supplement: Supplementary file 3 — Source data Fig. 2 [file 44319_2024_249_MOESM3_ESM.zip › FIG2/FIG.2A/HE TA BMD 6M.tif]

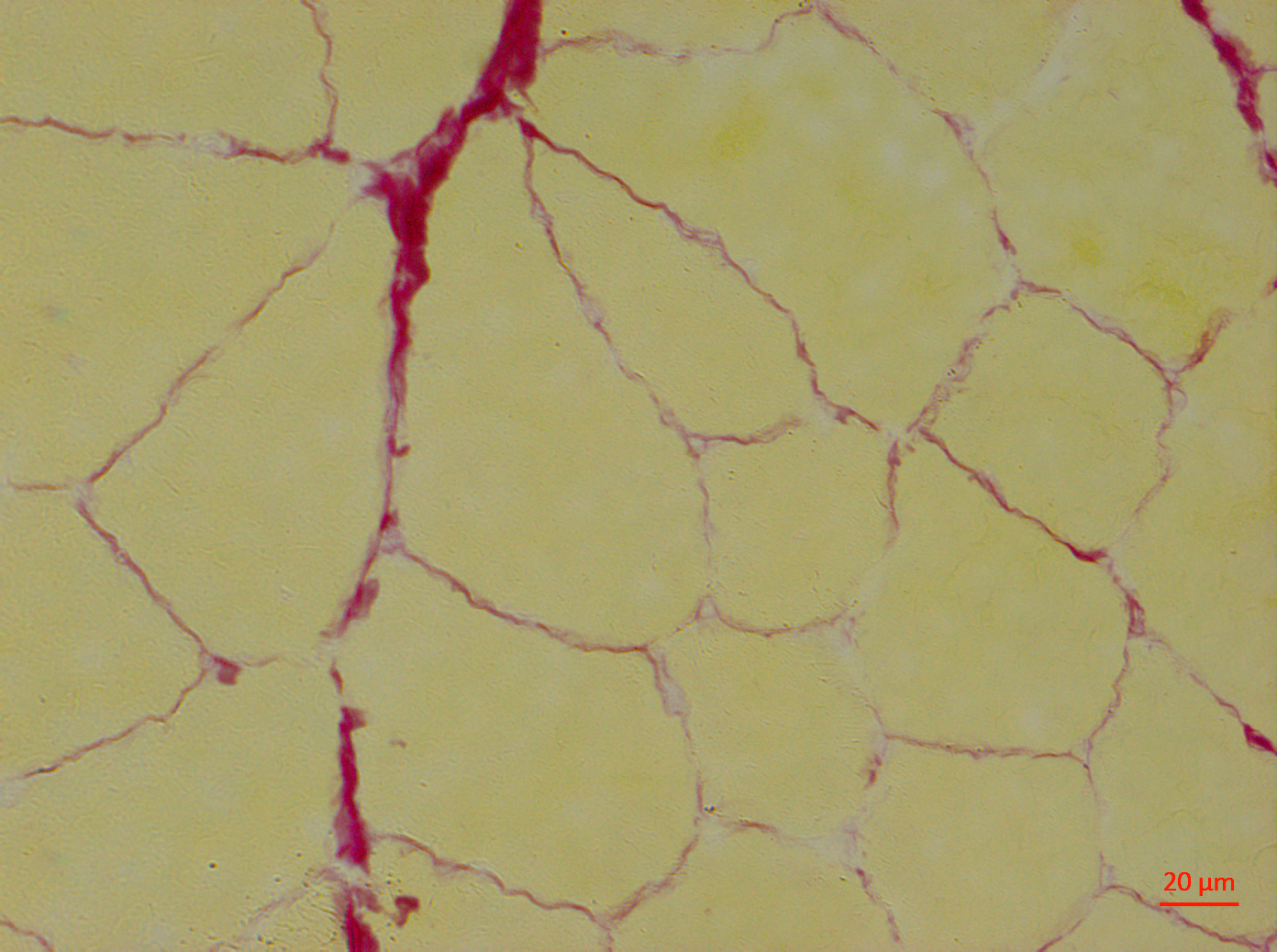

Supplement: Supplementary file 3 — Source data Fig. 2 [file 44319_2024_249_MOESM3_ESM.zip › FIG2/FIG.2A/SR TA BMD 12M.tiff]

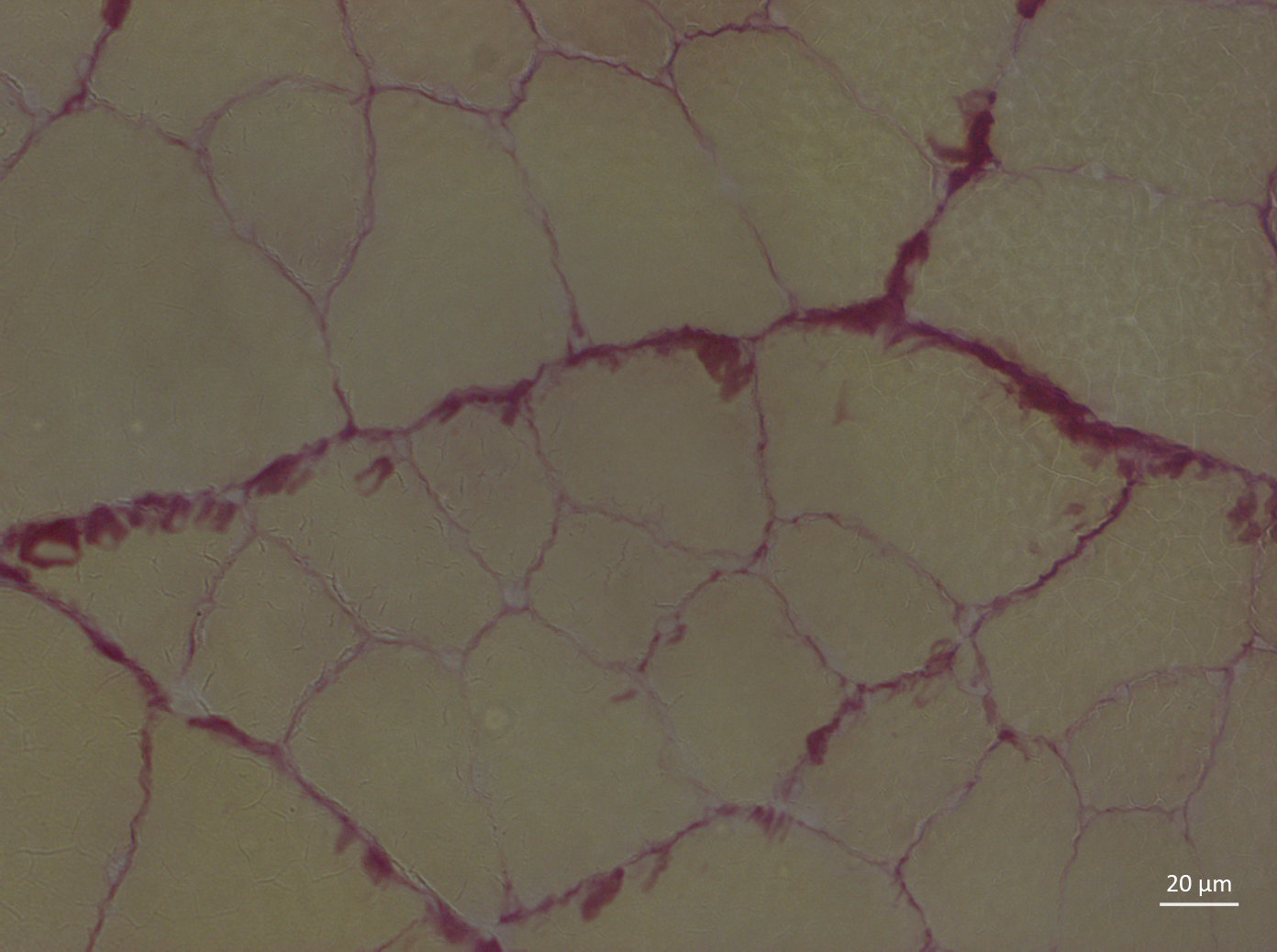

Supplement: Supplementary file 3 — Source data Fig. 2 [file 44319_2024_249_MOESM3_ESM.zip › FIG2/FIG.2A/SR TA BMD 6M.tif]

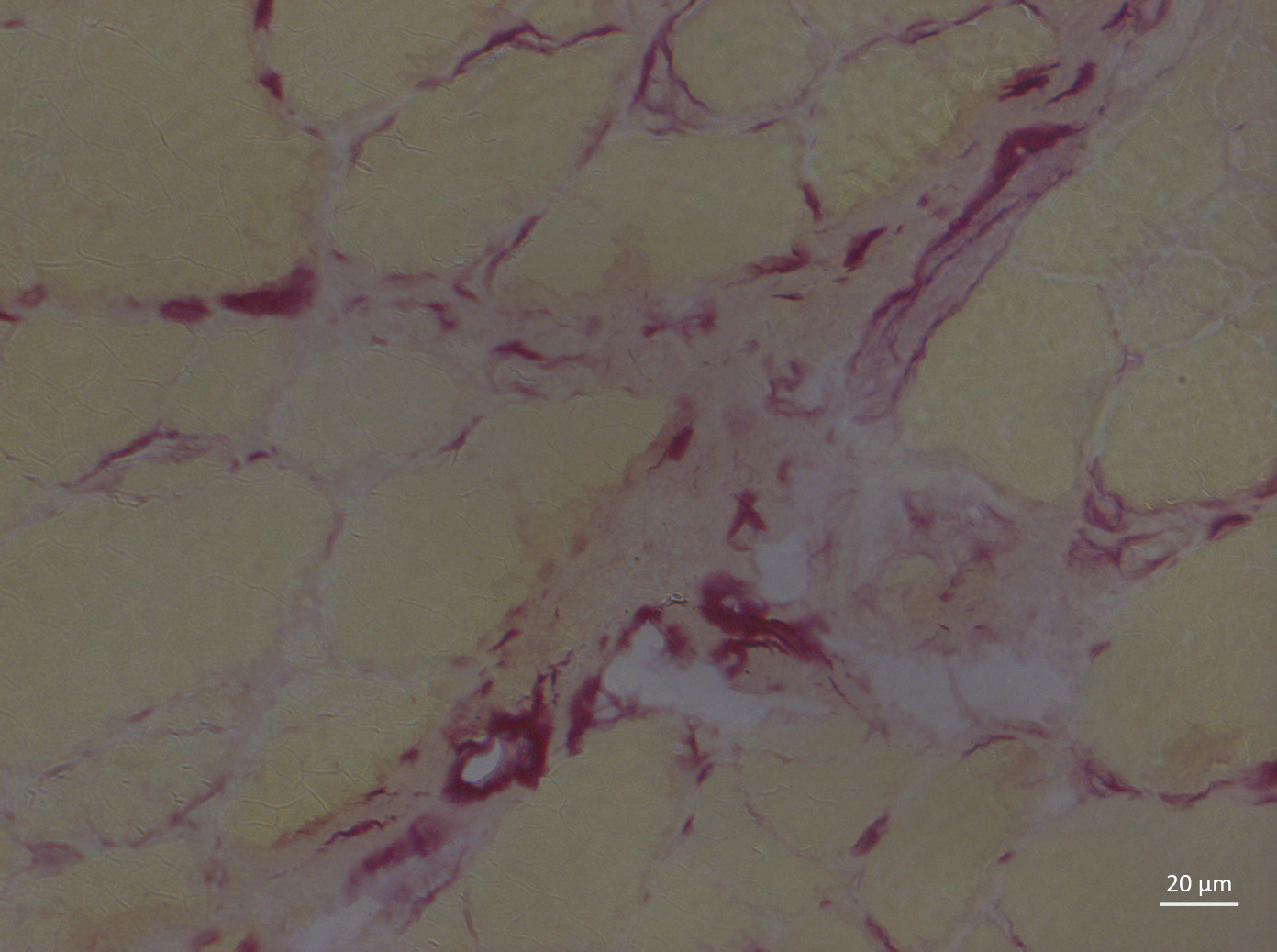

Supplement: Supplementary file 3 — Source data Fig. 2 [file 44319_2024_249_MOESM3_ESM.zip › FIG2/FIG.2A/SR TA DMD 6m X20 1.tif]

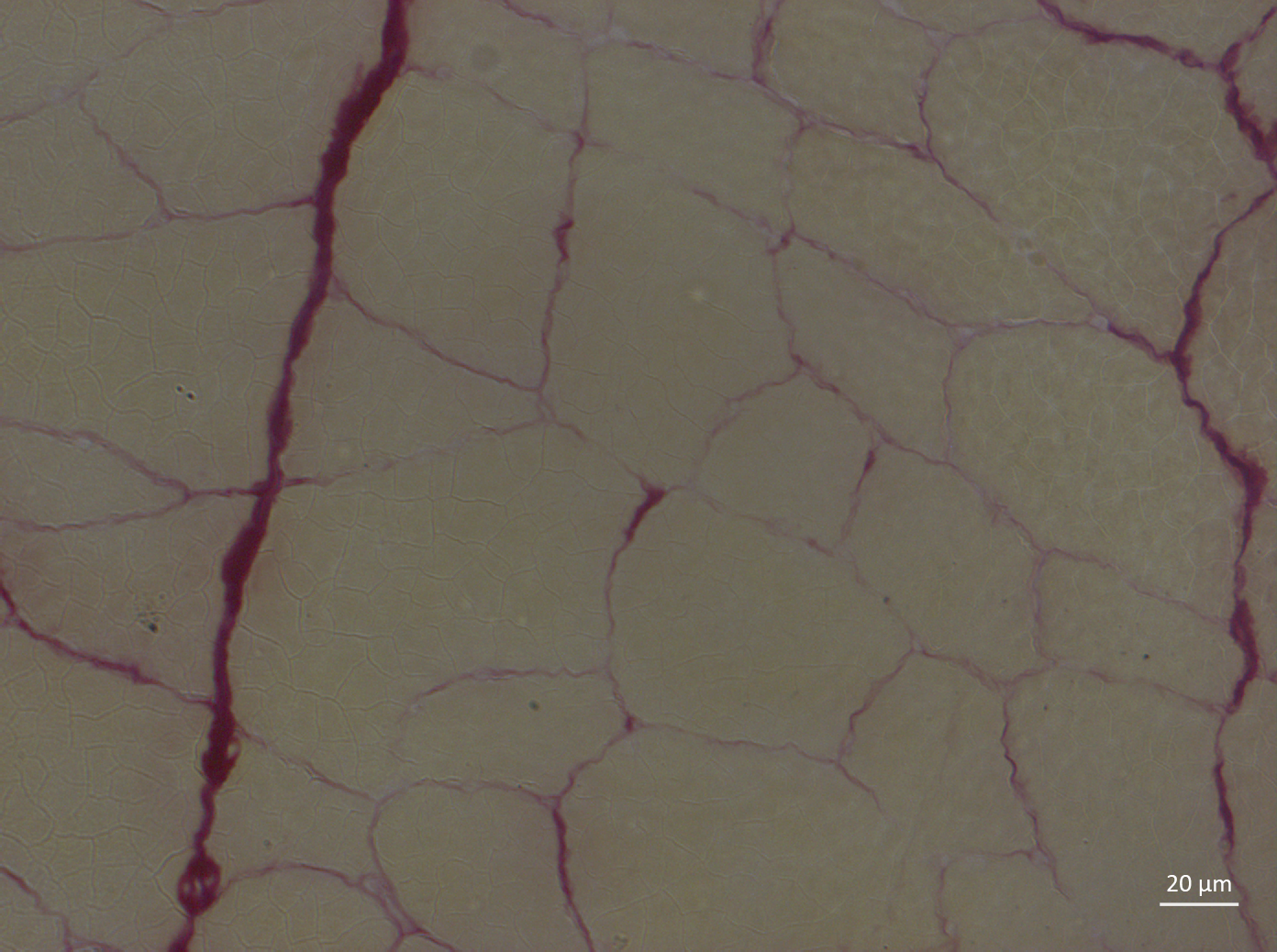

Supplement: Supplementary file 3 — Source data Fig. 2 [file 44319_2024_249_MOESM3_ESM.zip › FIG2/FIG.2A/SR TA WT 12M.tif]

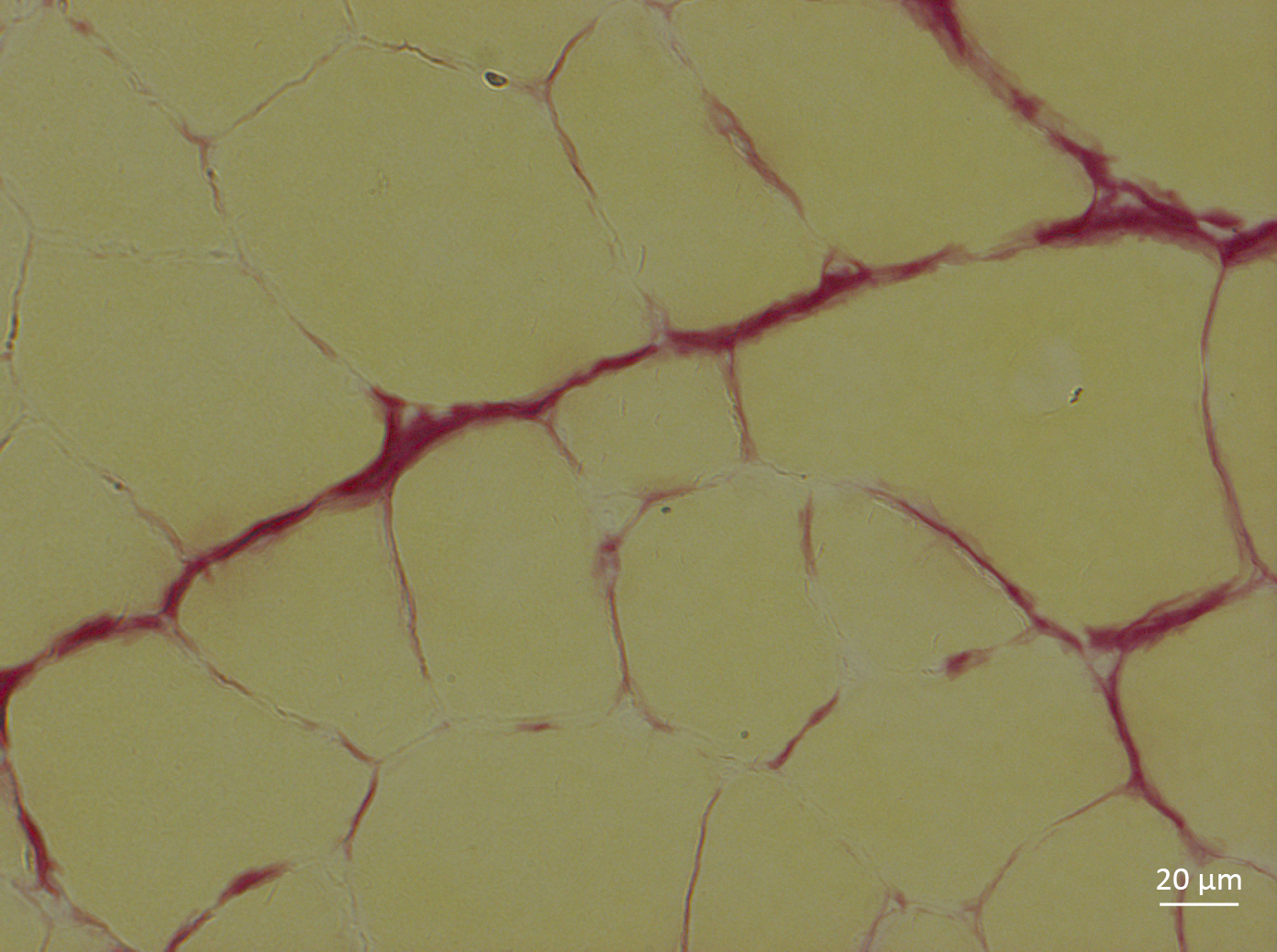

Supplement: Supplementary file 3 — Source data Fig. 2 [file 44319_2024_249_MOESM3_ESM.zip › FIG2/FIG.2A/SR TA WT 6Mtif.tif]

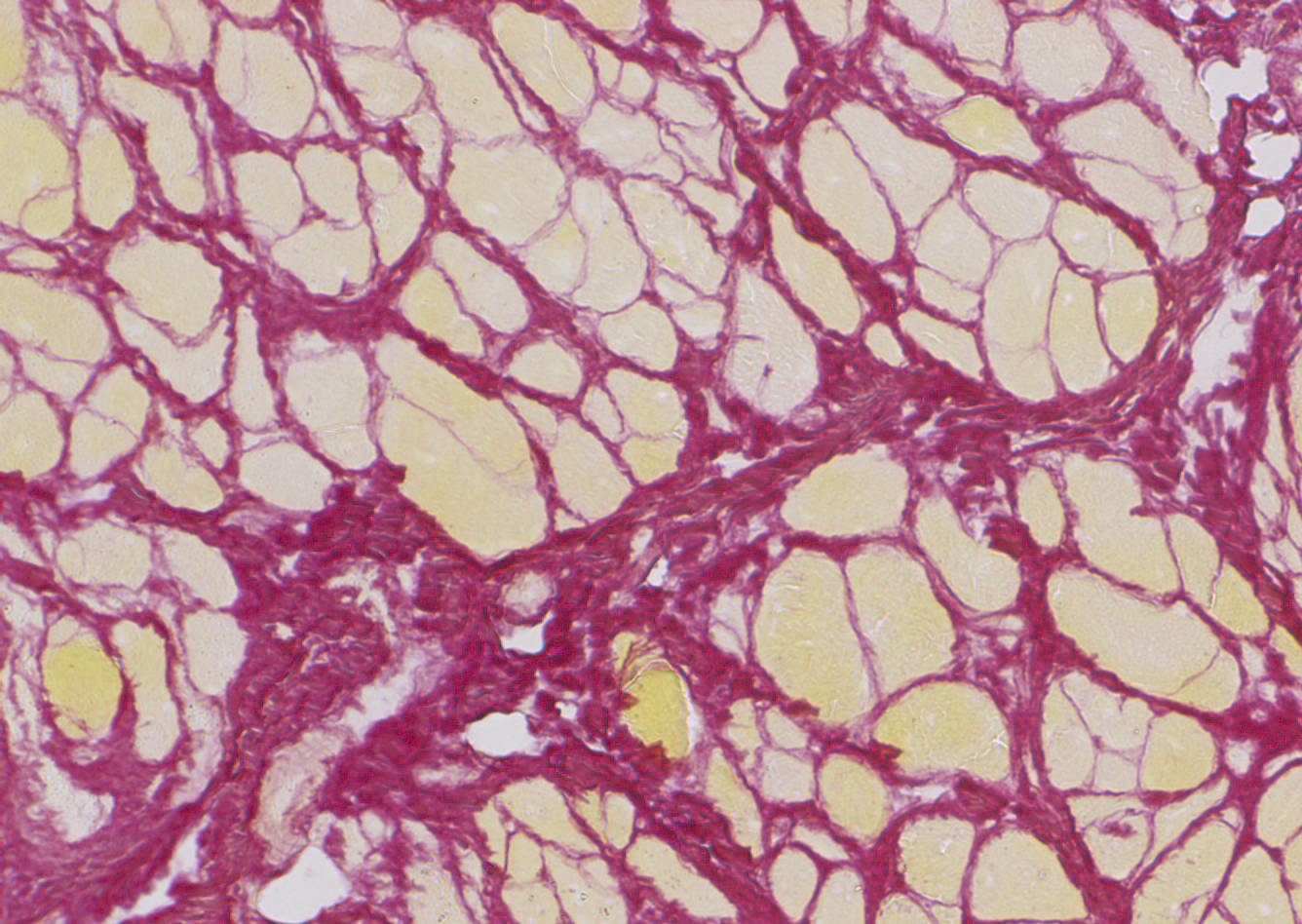

Supplement: Supplementary file 3 — Source data Fig. 2 [file 44319_2024_249_MOESM3_ESM.zip › FIG2/FIG.2A/SR TA DMD 12M.tif]

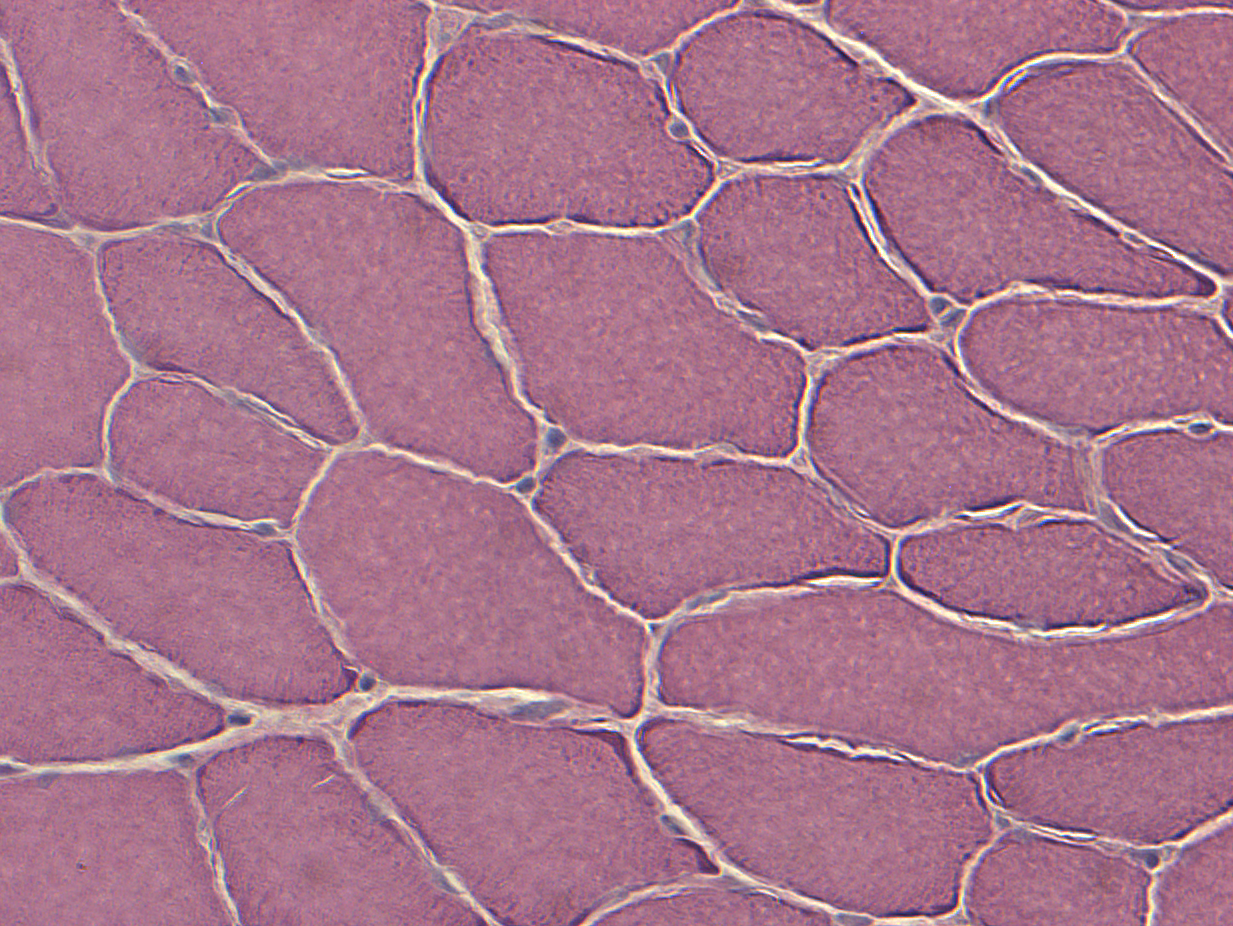

Supplement: Supplementary file 3 — Source data Fig. 2 [file 44319_2024_249_MOESM3_ESM.zip › FIG2/FIG.2A/HE WT 6M.tif]

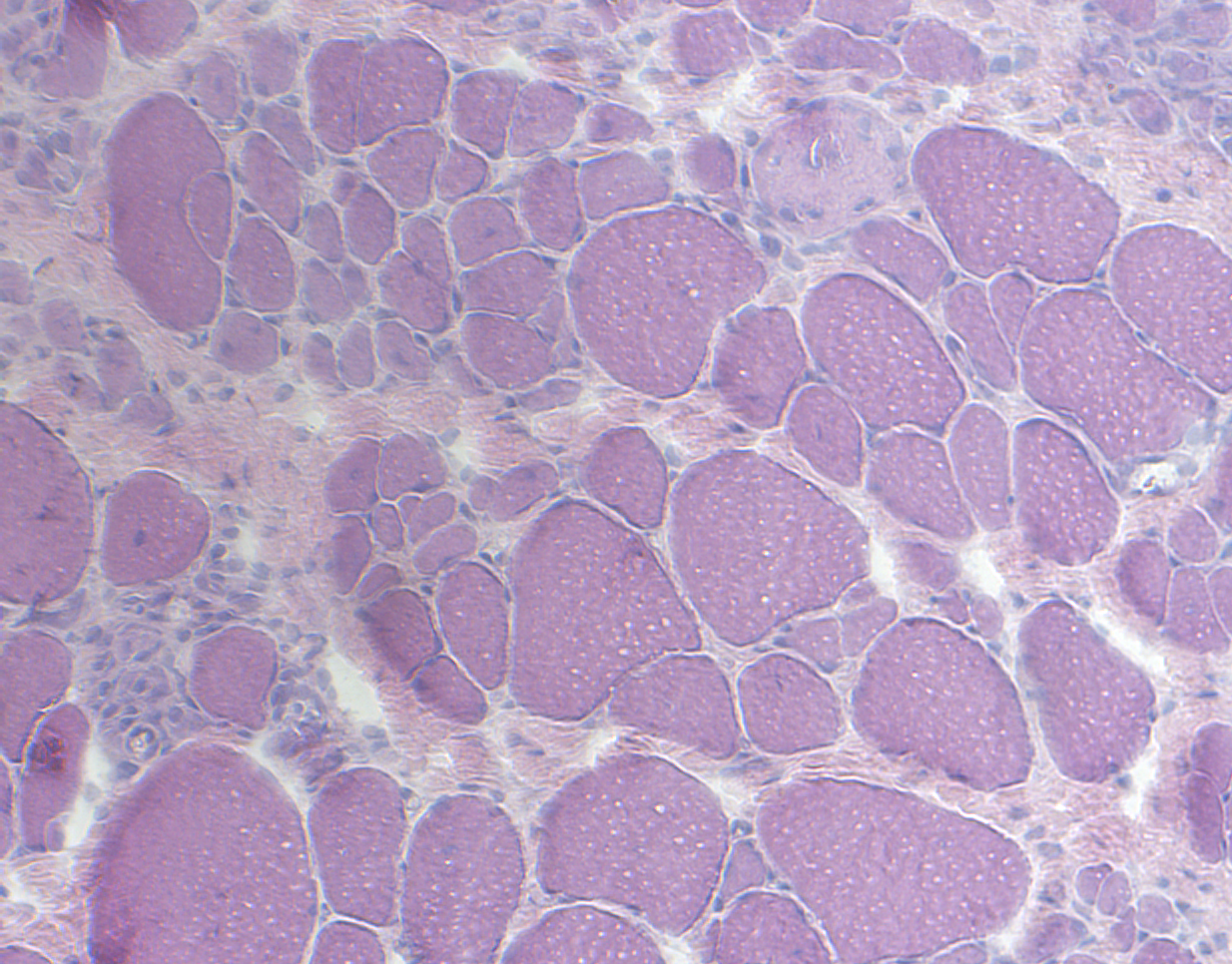

Supplement: Supplementary file 3 — Source data Fig. 2 [file 44319_2024_249_MOESM3_ESM.zip › FIG2/FIG.2A/HE TA DMD 12m.tif]

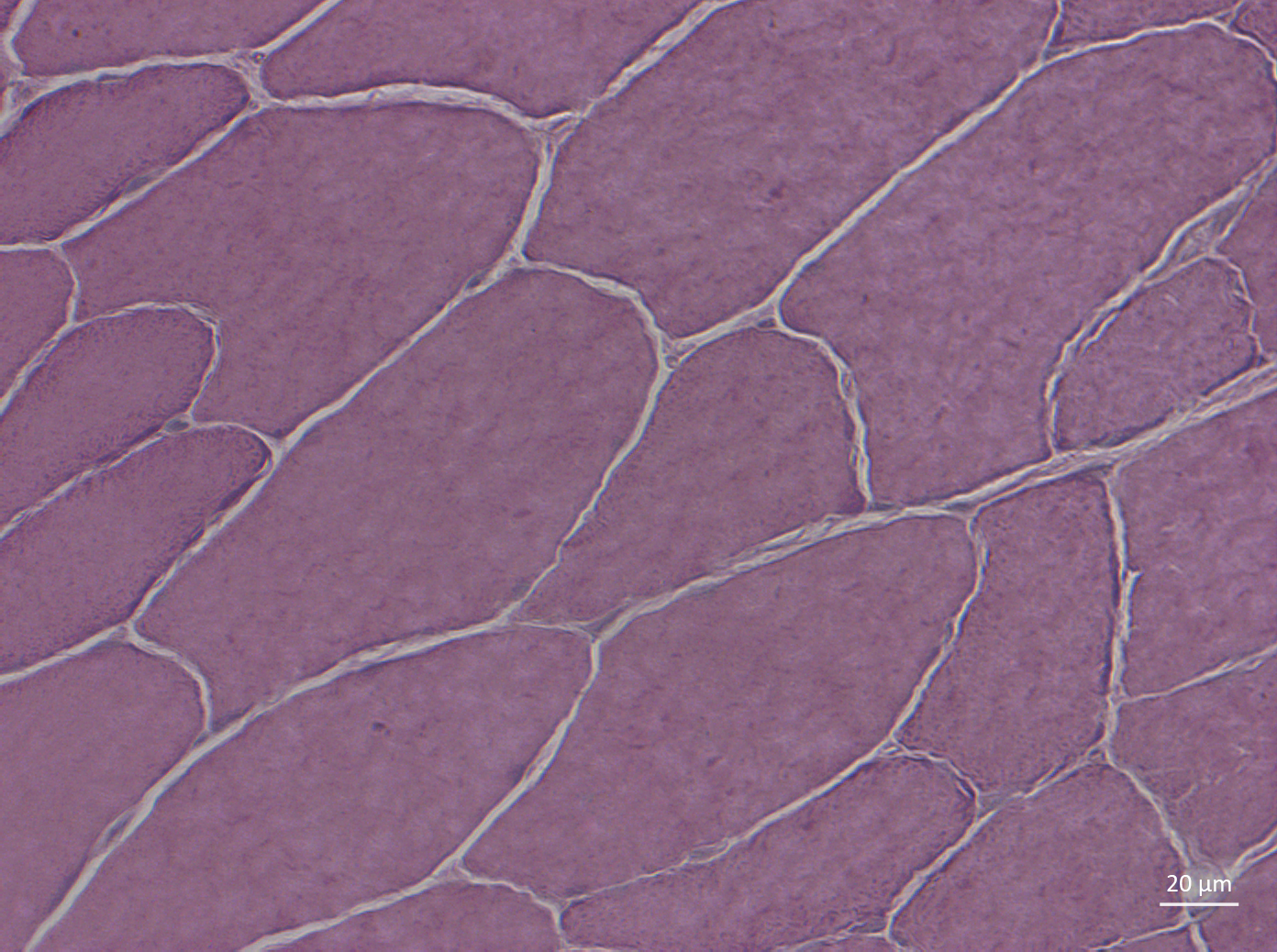

Supplement: Supplementary file 3 — Source data Fig. 2 [file 44319_2024_249_MOESM3_ESM.zip › FIG2/FIG.2A/HE WT 12m.tif]

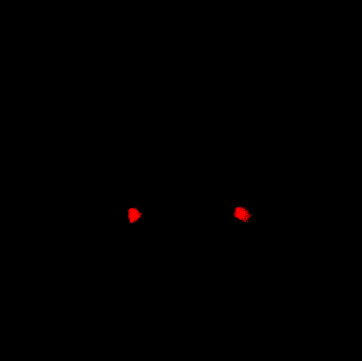

Supplement: Supplementary file 4 — Source data Fig. 3 [file 44319_2024_249_MOESM4_ESM.zip › FIG3/FIG.3A/BMD 6m 743 5_Cy5-T1.tif]

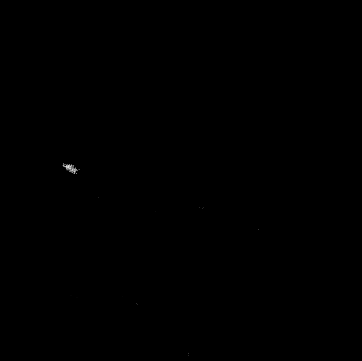

Supplement: Supplementary file 4 — Source data Fig. 3 [file 44319_2024_249_MOESM4_ESM.zip › FIG3/FIG.3A/BMD 6m 743 5_DsRed-T2.tif]

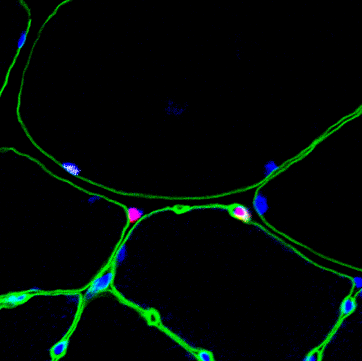

Supplement: Supplementary file 4 — Source data Fig. 3 [file 44319_2024_249_MOESM4_ESM.zip › FIG3/FIG.3A/BMD 6m 743 5.tif]

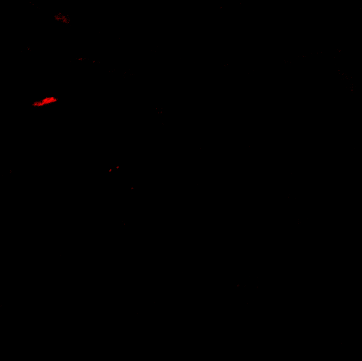

Supplement: Supplementary file 4 — Source data Fig. 3 [file 44319_2024_249_MOESM4_ESM.zip › FIG3/FIG.3A/DMD 6m 3_DsRed-T2.tif]

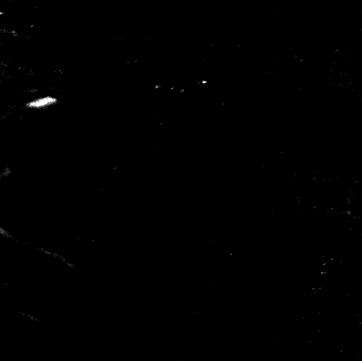

Supplement: Supplementary file 4 — Source data Fig. 3 [file 44319_2024_249_MOESM4_ESM.zip › FIG3/FIG.3A/DMD 6m 3_EGFP-T3.tif]

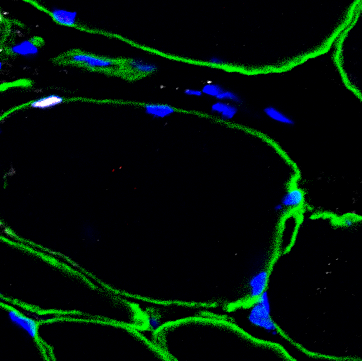

Supplement: Supplementary file 4 — Source data Fig. 3 [file 44319_2024_249_MOESM4_ESM.zip › FIG3/FIG.3A/DMD 6m 3.tif]

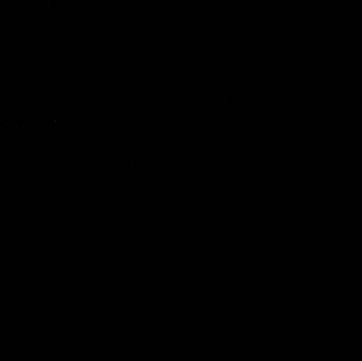

Supplement: Supplementary file 4 — Source data Fig. 3 [file 44319_2024_249_MOESM4_ESM.zip › FIG3/FIG.3A/WT 6m 527 2_Cy5-T1.tif]

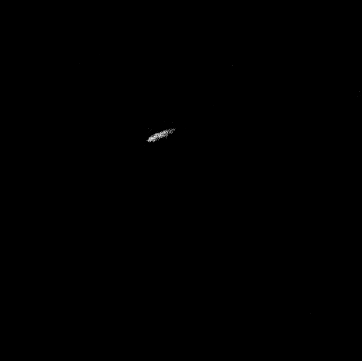

Supplement: Supplementary file 4 — Source data Fig. 3 [file 44319_2024_249_MOESM4_ESM.zip › FIG3/FIG.3A/WT 6m 527 2_DsRed-T2.tif]

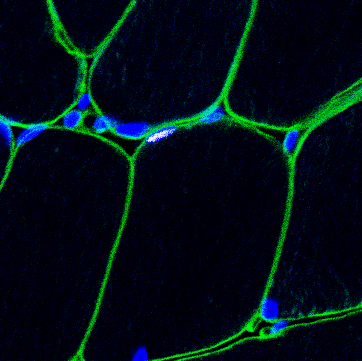

Supplement: Supplementary file 4 — Source data Fig. 3 [file 44319_2024_249_MOESM4_ESM.zip › FIG3/FIG.3A/WT 6m 527 2.tif]

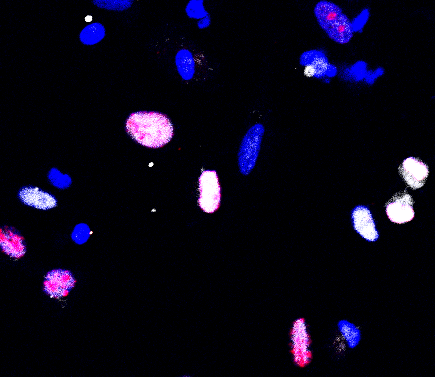

Supplement: Supplementary file 4 — Source data Fig. 3 [file 44319_2024_249_MOESM4_ESM.zip › FIG3/FIG.3D/DMD-Snap-21349_c1+2+3.tif]

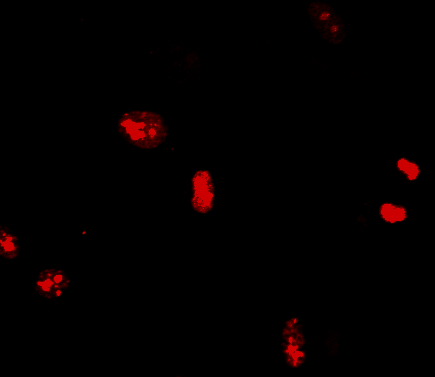

Supplement: Supplementary file 4 — Source data Fig. 3 [file 44319_2024_249_MOESM4_ESM.zip › FIG3/FIG.3D/DMD-Snap-21349_c2.tif]

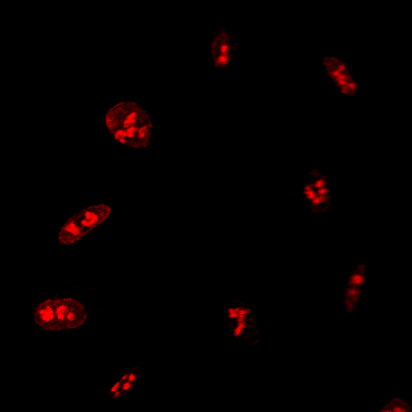

Supplement: Supplementary file 4 — Source data Fig. 3 [file 44319_2024_249_MOESM4_ESM.zip › FIG3/FIG.3D/11m Prolif WT A4_DsRed-T2.tif]

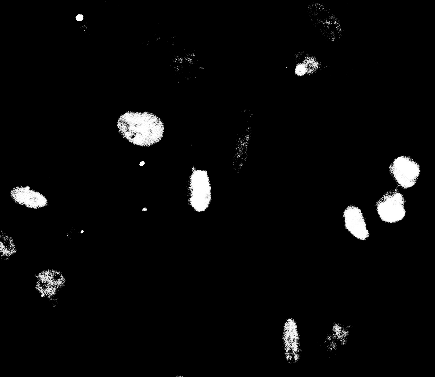

Supplement: Supplementary file 4 — Source data Fig. 3 [file 44319_2024_249_MOESM4_ESM.zip › FIG3/FIG.3D/DMD-Snap-21349_c1.tif]

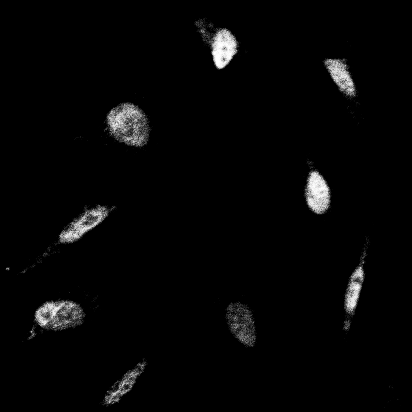

Supplement: Supplementary file 4 — Source data Fig. 3 [file 44319_2024_249_MOESM4_ESM.zip › FIG3/FIG.3D/11m Prolif WT A4_EGFP-T3.tif]

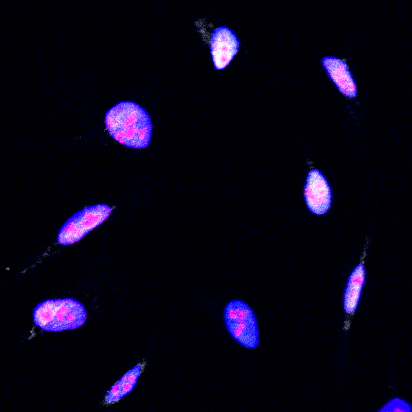

Supplement: Supplementary file 4 — Source data Fig. 3 [file 44319_2024_249_MOESM4_ESM.zip › FIG3/FIG.3D/11m Prolif WT A4.tif]

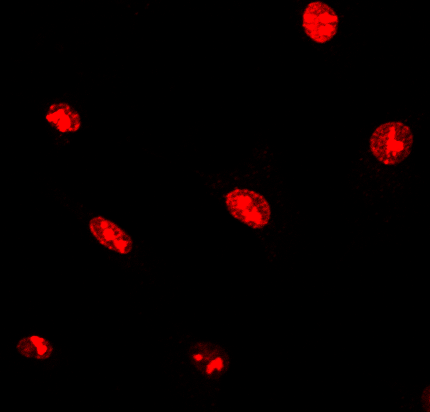

Supplement: Supplementary file 4 — Source data Fig. 3 [file 44319_2024_249_MOESM4_ESM.zip › FIG3/FIG.3D/11m Prolif BMD 23 5_DsRed-T2.tif]

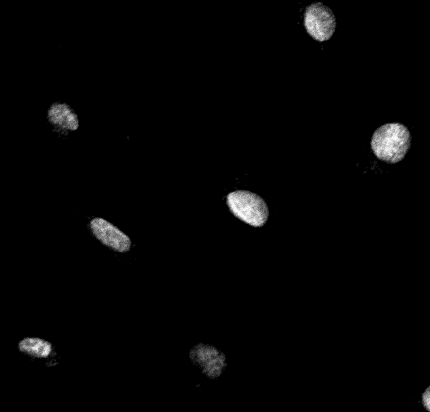

Supplement: Supplementary file 4 — Source data Fig. 3 [file 44319_2024_249_MOESM4_ESM.zip › FIG3/FIG.3D/11m Prolif BMD 23 5_EGFP-T3.tif]

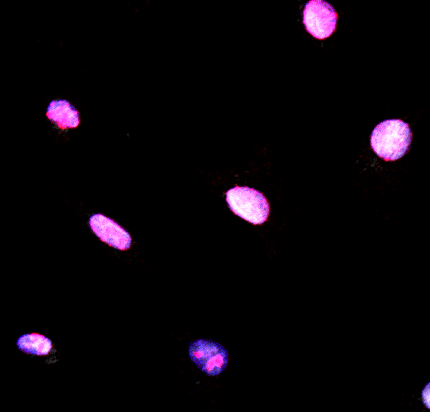

Supplement: Supplementary file 4 — Source data Fig. 3 [file 44319_2024_249_MOESM4_ESM.zip › FIG3/FIG.3D/11m Prolif BMD 23 5.tif]

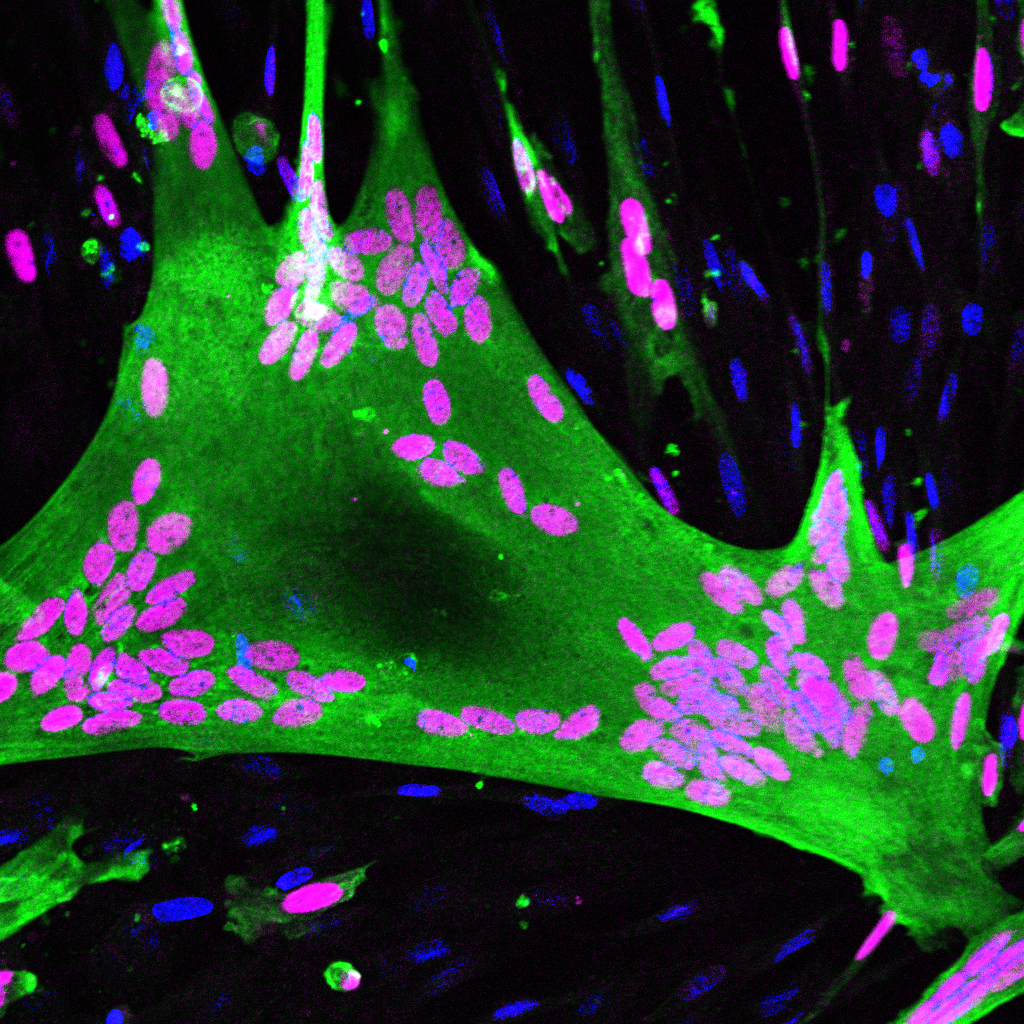

Supplement: Supplementary file 4 — Source data Fig. 3 [file 44319_2024_249_MOESM4_ESM.zip › FIG3/FIG.3G/WT.tif]

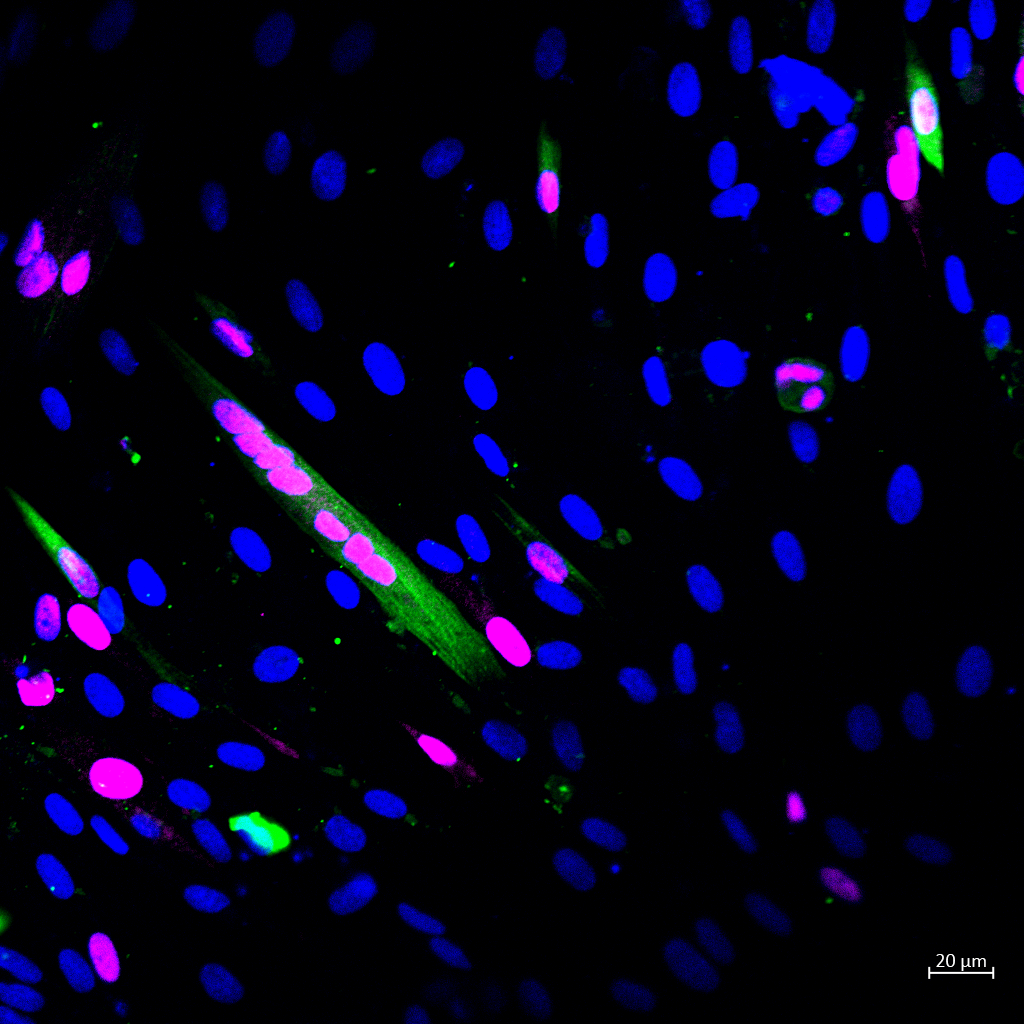

Supplement: Supplementary file 4 — Source data Fig. 3 [file 44319_2024_249_MOESM4_ESM.zip › FIG3/FIG.3G/DMD.tif]

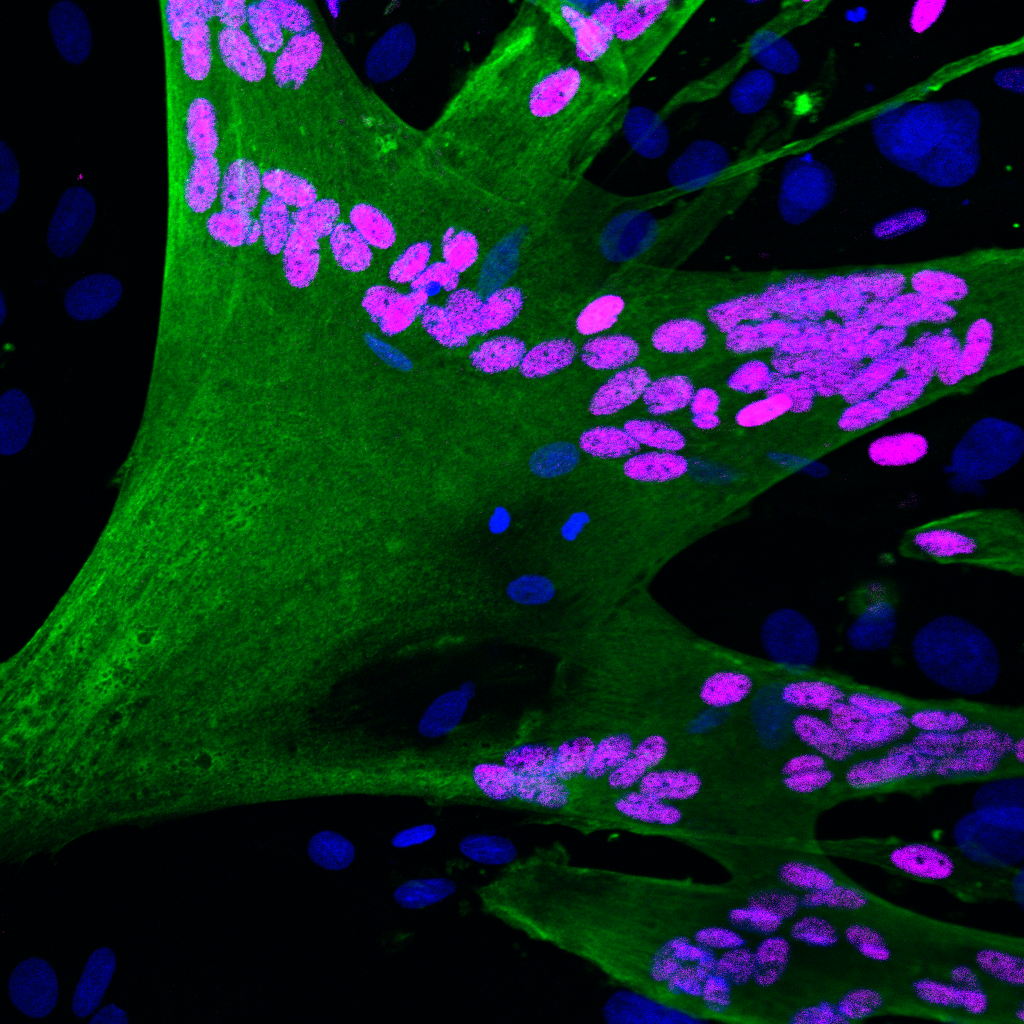

Supplement: Supplementary file 4 — Source data Fig. 3 [file 44319_2024_249_MOESM4_ESM.zip › FIG3/FIG.3G/BMD.tif]

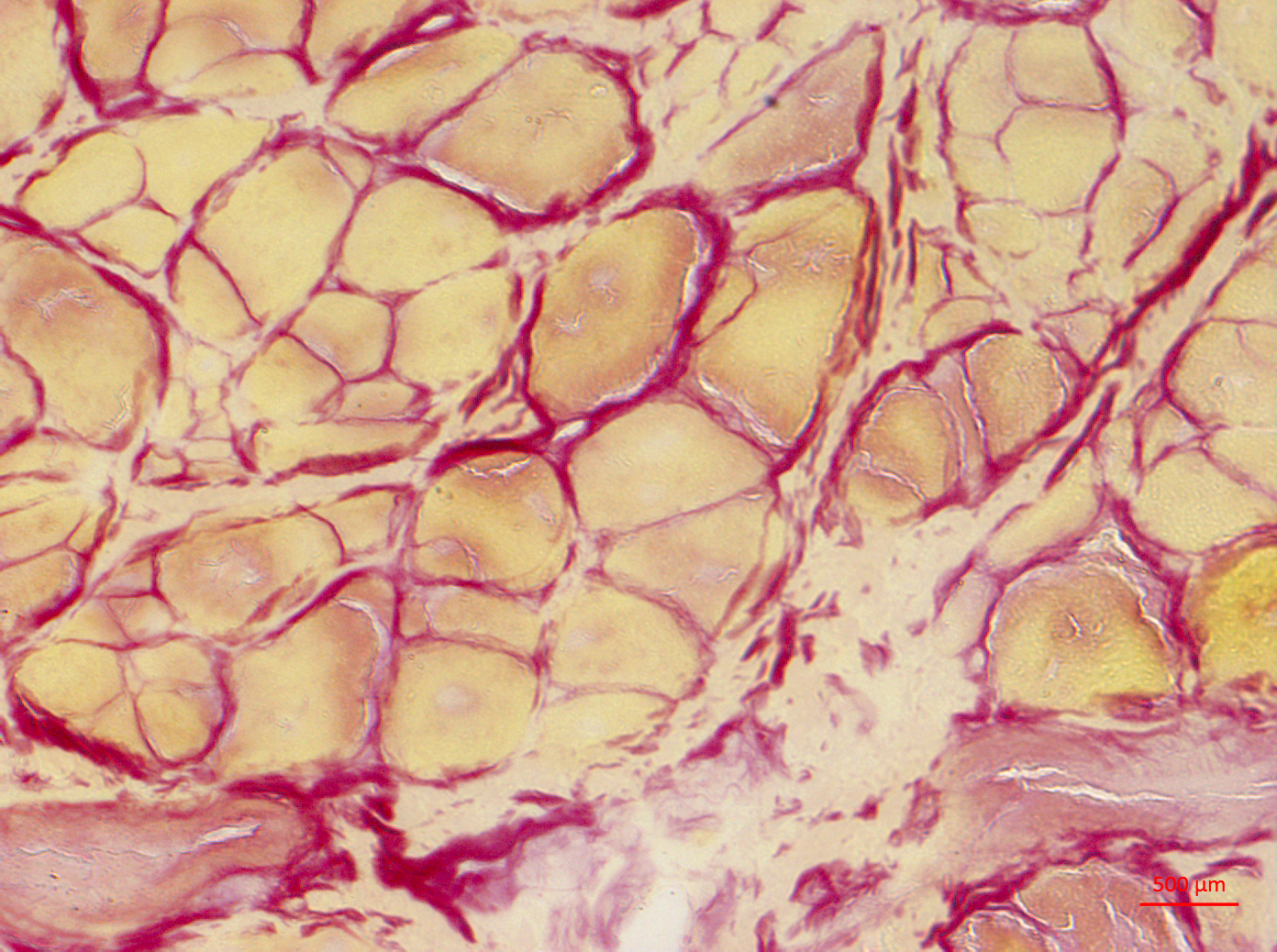

Supplement: Supplementary file 5 — Source data Fig. 4 [file 44319_2024_249_MOESM5_ESM.zip › FIG4/FIG.4A/SR DIA DMD 6mX20 2.tif]

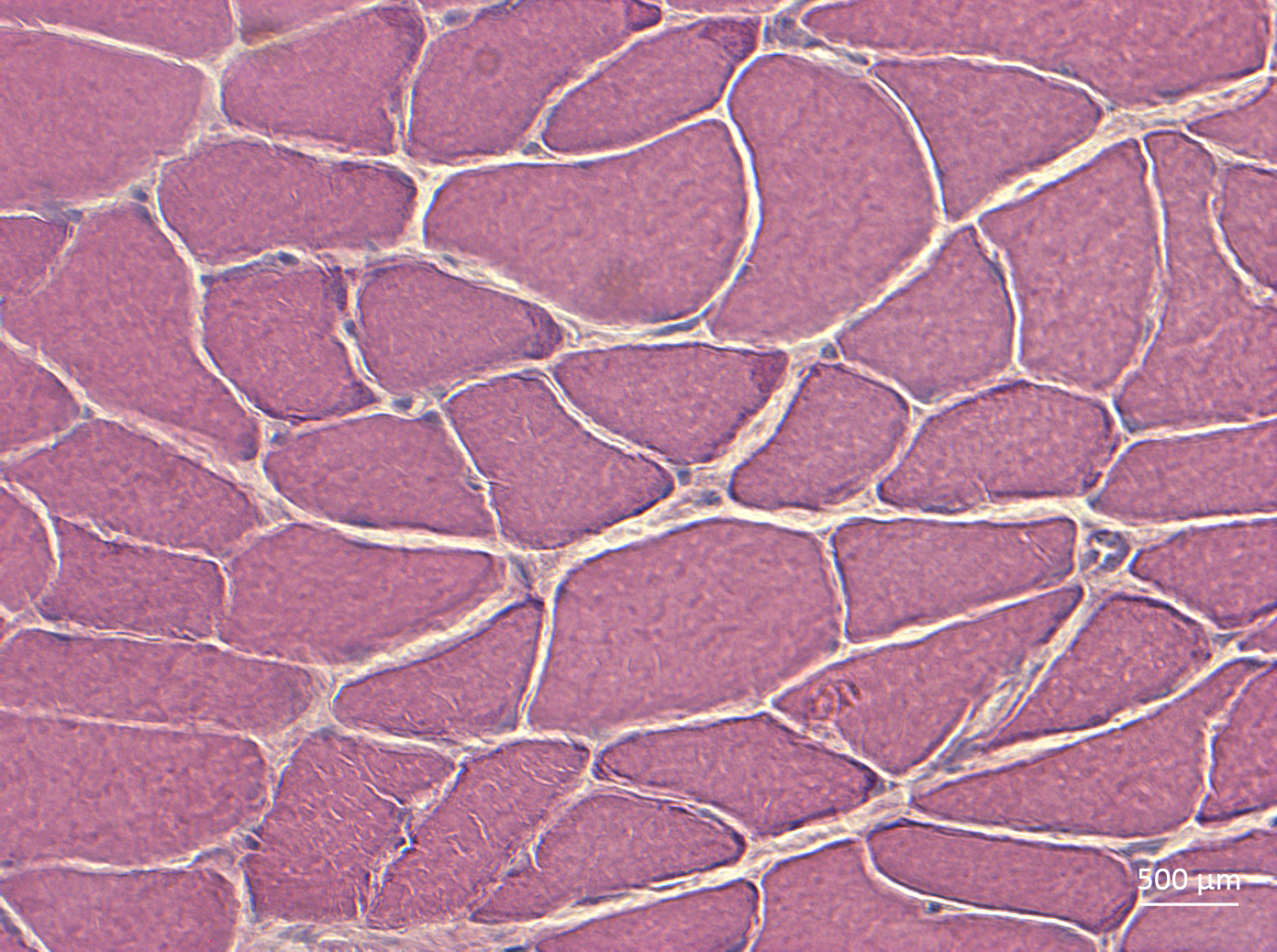

Supplement: Supplementary file 5 — Source data Fig. 4 [file 44319_2024_249_MOESM5_ESM.zip › FIG4/FIG.4A/DIA RAT 6m wt 230 X20 5.tif]

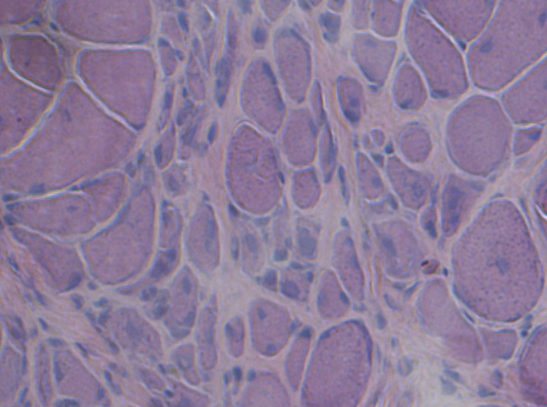

Supplement: Supplementary file 5 — Source data Fig. 4 [file 44319_2024_249_MOESM5_ESM.zip › FIG4/FIG.4A/HE DIA DMD 6m .tif]

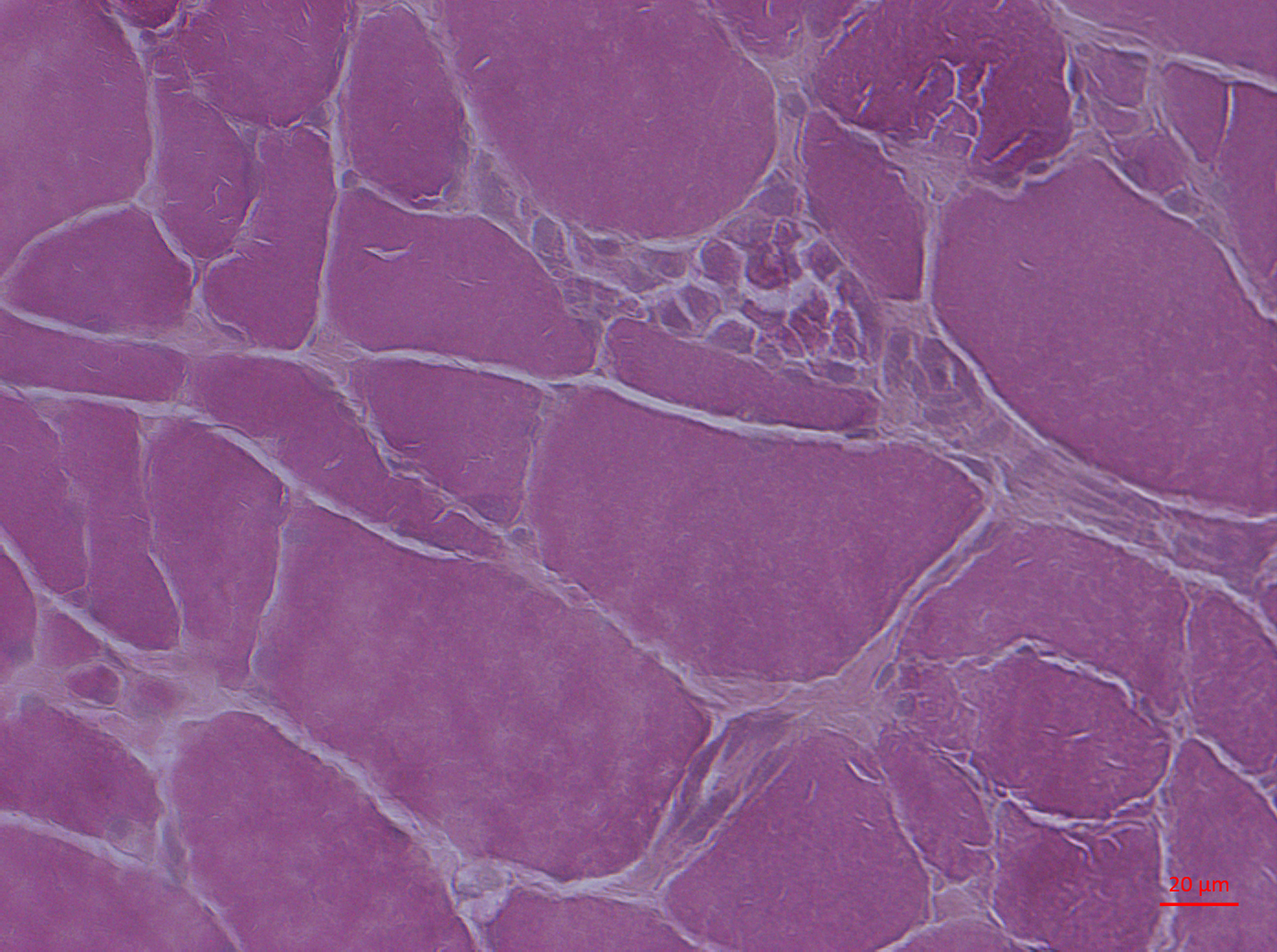

Supplement: Supplementary file 5 — Source data Fig. 4 [file 44319_2024_249_MOESM5_ESM.zip › FIG4/FIG.4A/HE DIA BMD 12mX20 9.tif]

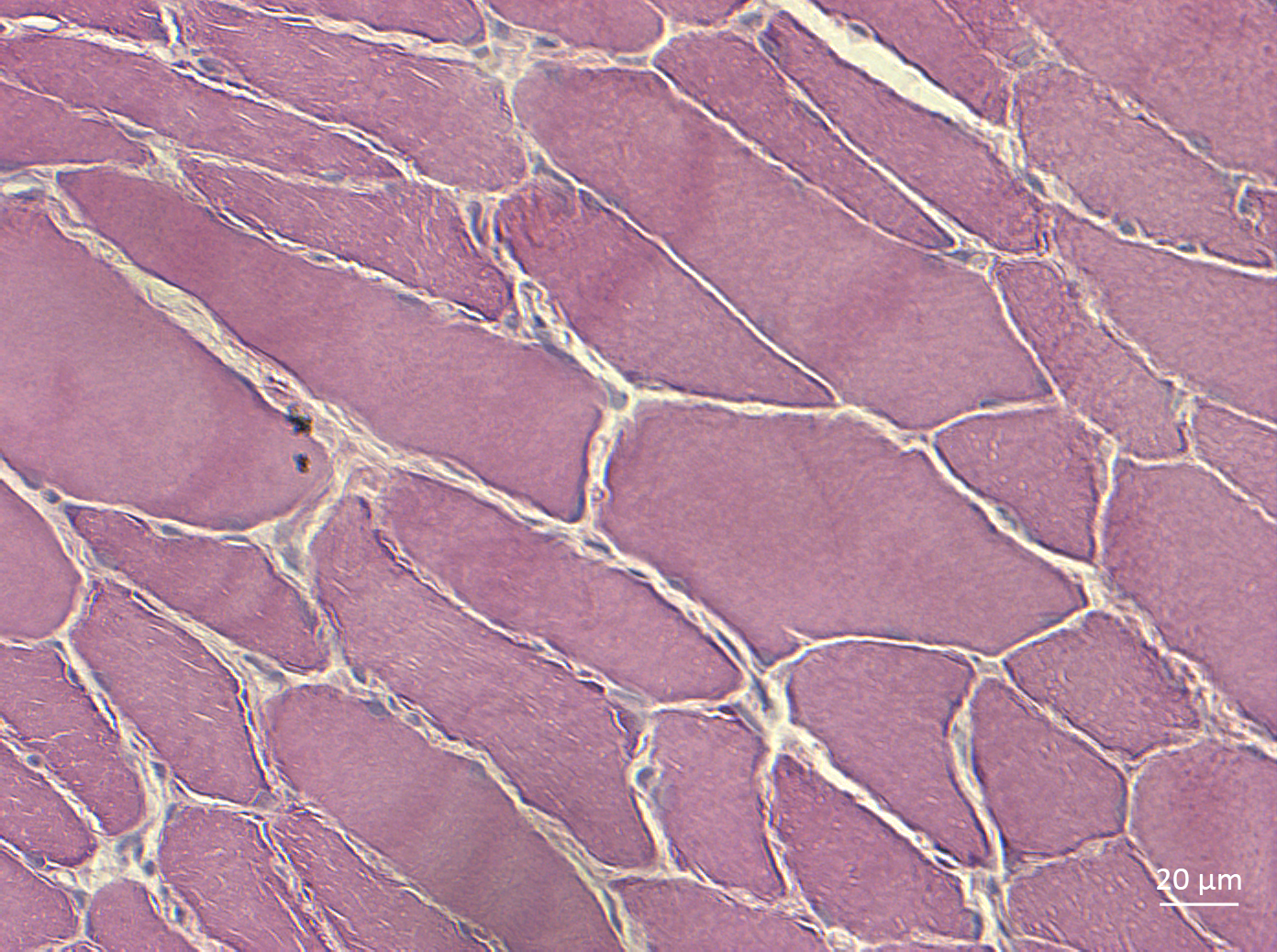

Supplement: Supplementary file 5 — Source data Fig. 4 [file 44319_2024_249_MOESM5_ESM.zip › FIG4/FIG.4A/HE DIA BMD 6M.tif]

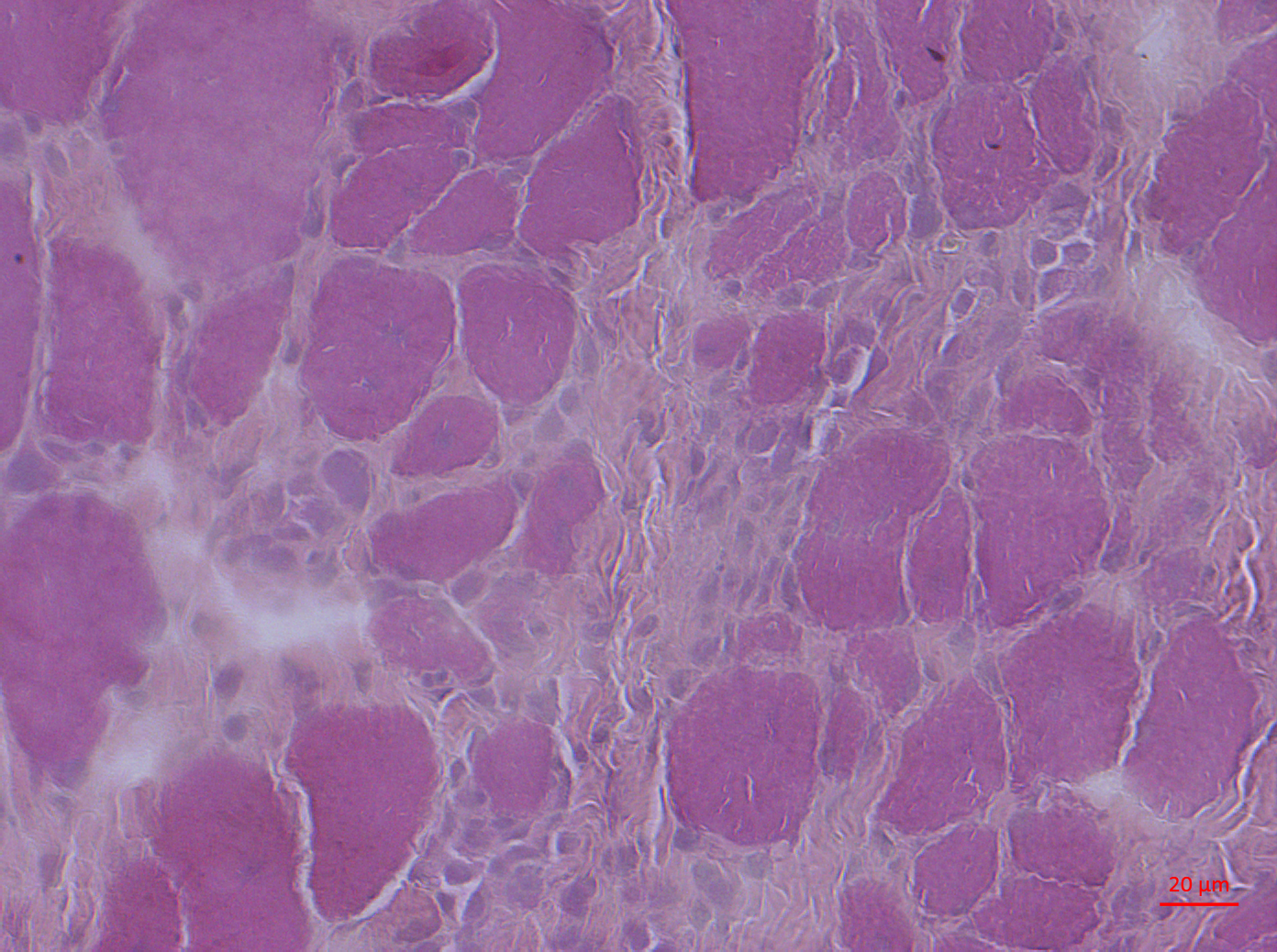

Supplement: Supplementary file 5 — Source data Fig. 4 [file 44319_2024_249_MOESM5_ESM.zip › FIG4/FIG.4A/HE DIA DMD 12mX20 1.tif]

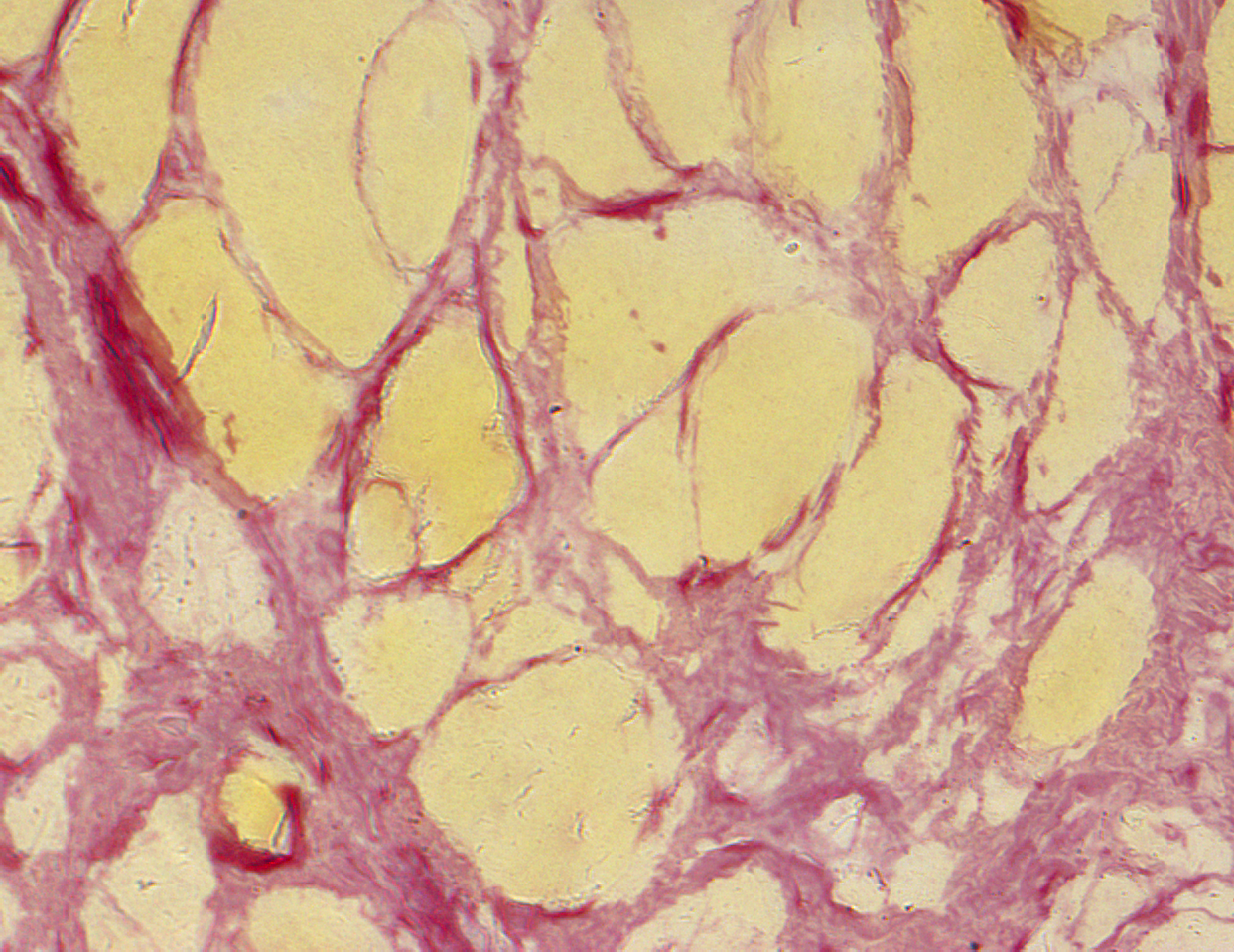

Supplement: Supplementary file 5 — Source data Fig. 4 [file 44319_2024_249_MOESM5_ESM.zip › FIG4/FIG.4A/SR DIA DMD 12m X20 19.tiff]

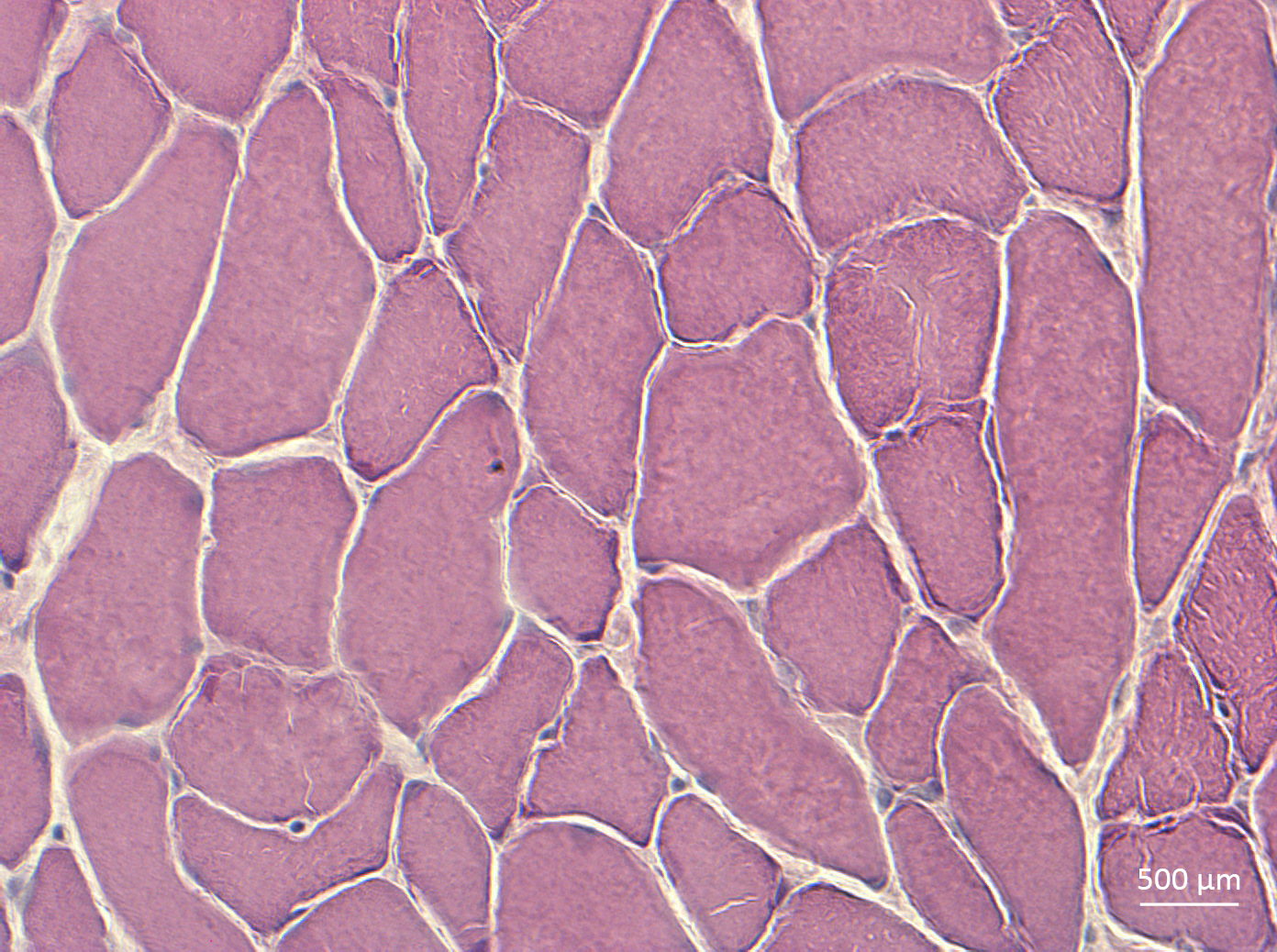

Supplement: Supplementary file 5 — Source data Fig. 4 [file 44319_2024_249_MOESM5_ESM.zip › FIG4/FIG.4A/DIA RAT 12m wt 240 X20 1.tif]

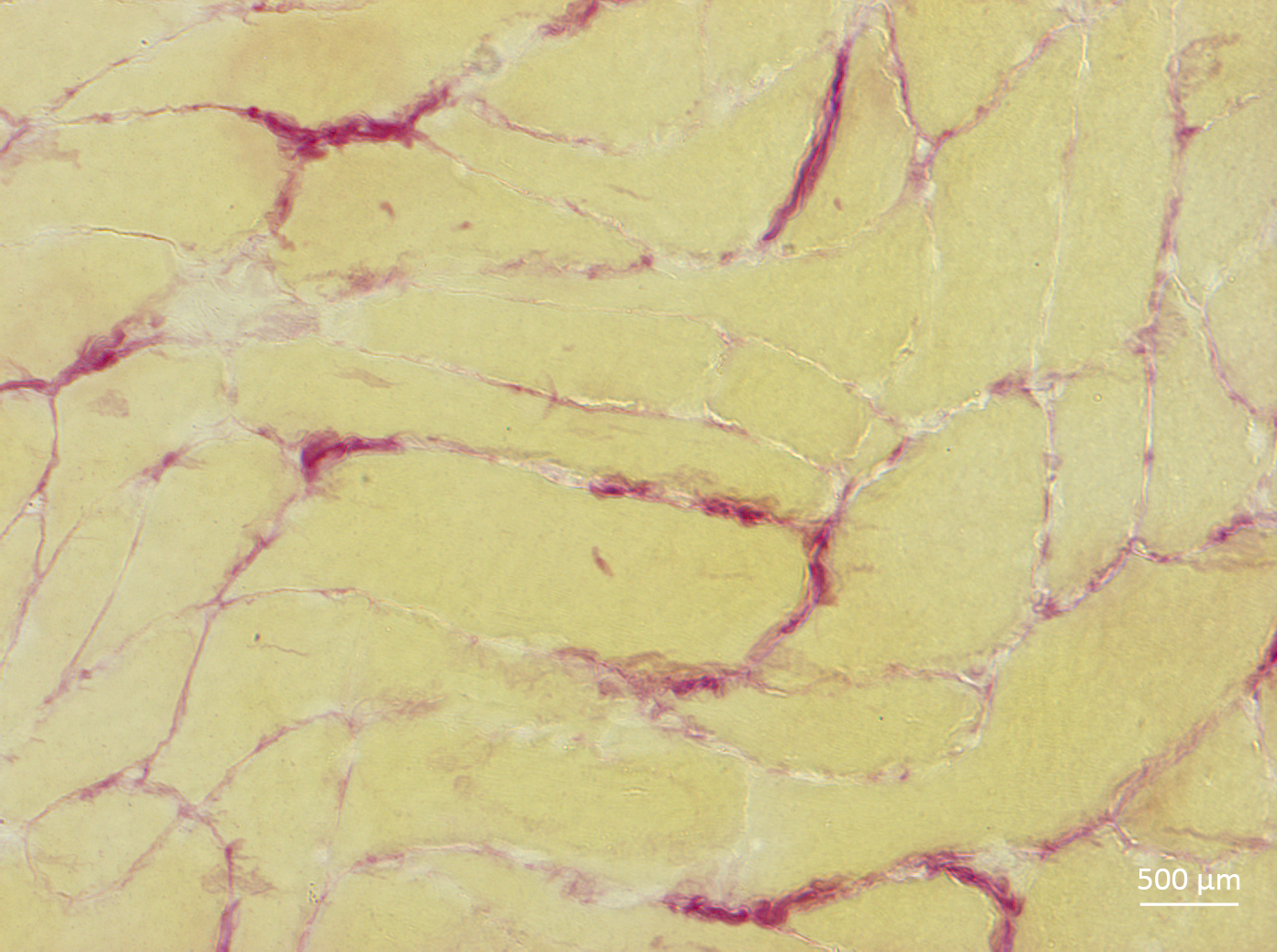

Supplement: Supplementary file 5 — Source data Fig. 4 [file 44319_2024_249_MOESM5_ESM.zip › FIG4/FIG.4A/SR DIA WT 6M.tif]

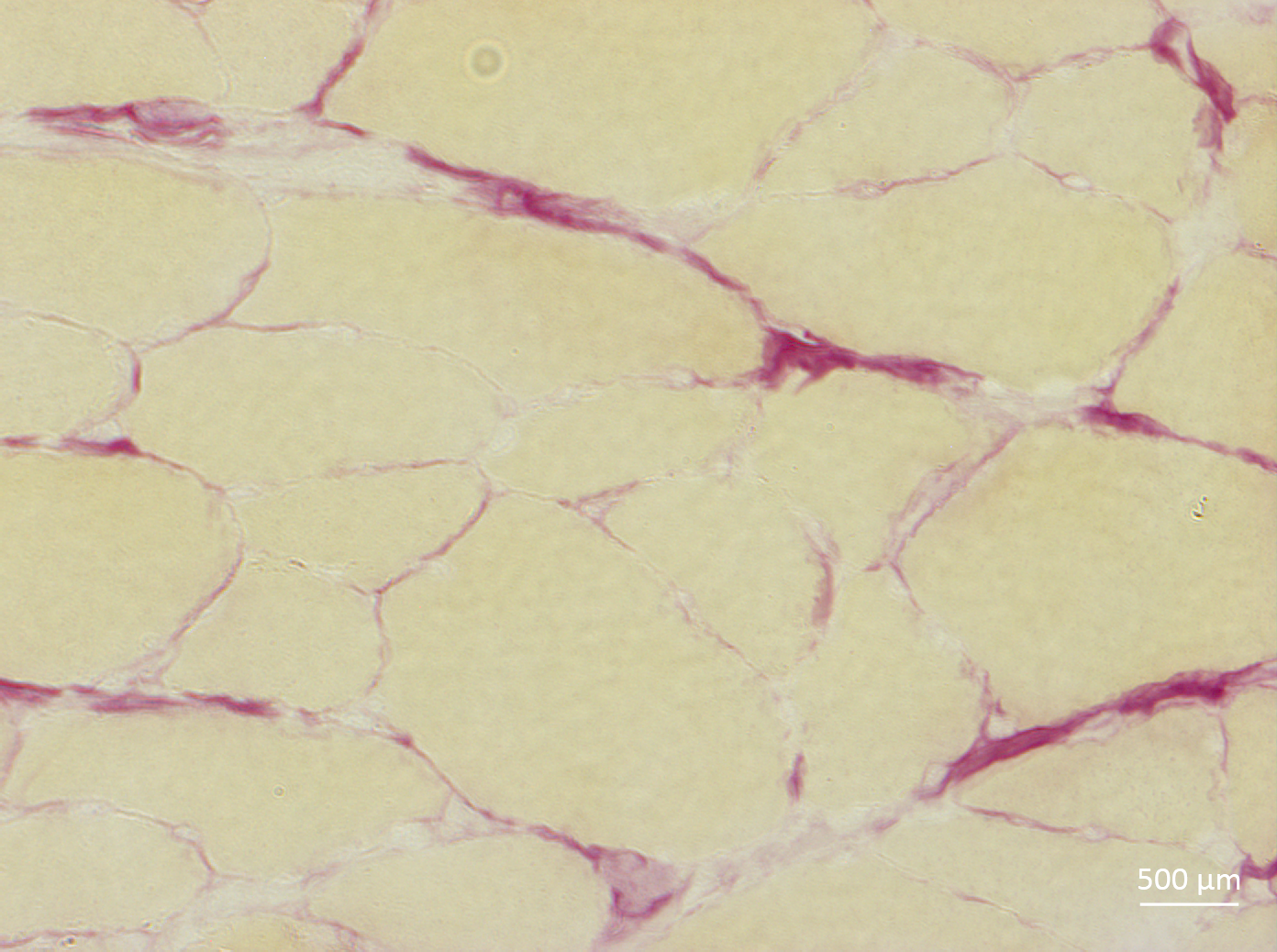

Supplement: Supplementary file 5 — Source data Fig. 4 [file 44319_2024_249_MOESM5_ESM.zip › FIG4/FIG.4A/SR DIA BMD 6M.tif]

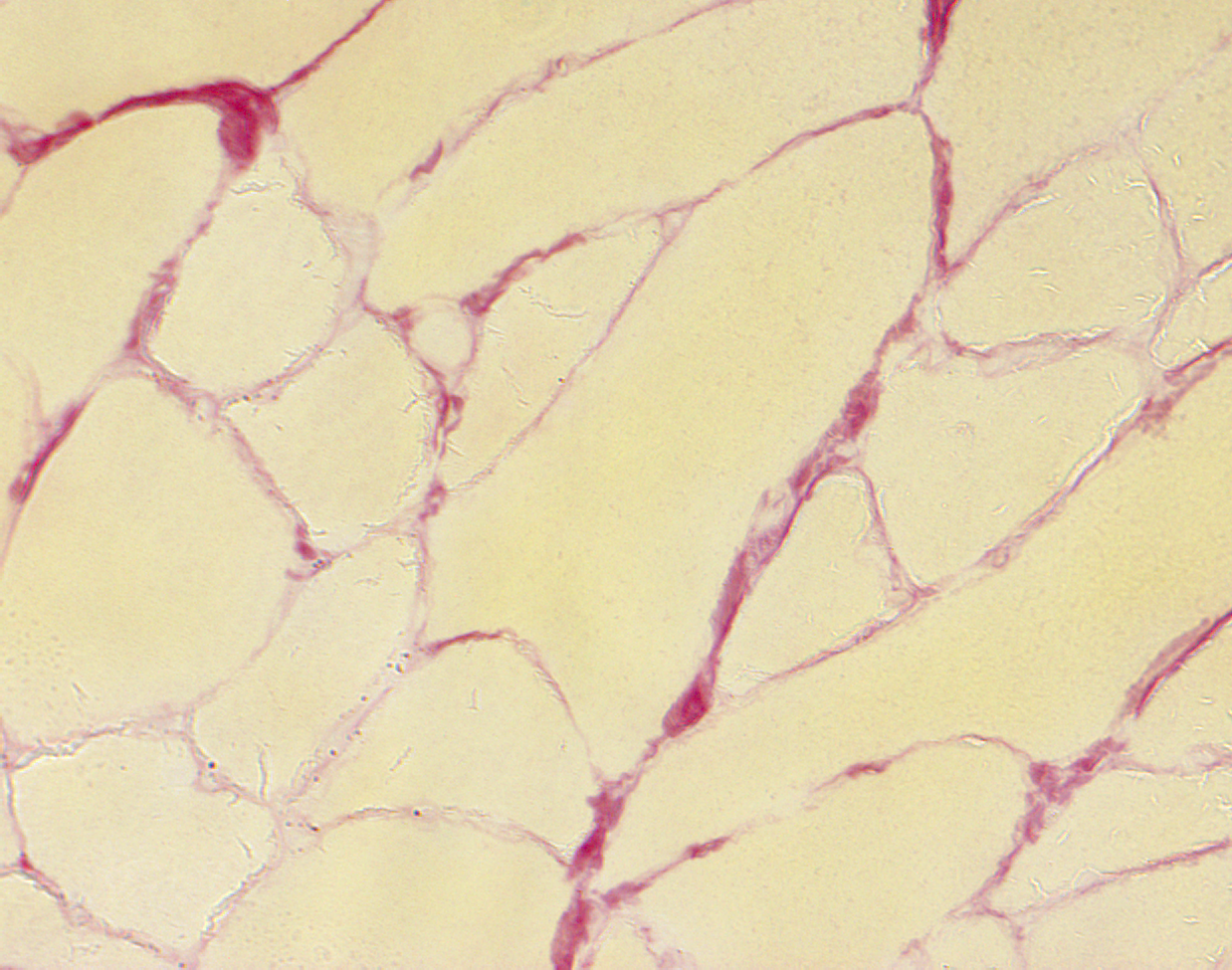

Supplement: Supplementary file 5 — Source data Fig. 4 [file 44319_2024_249_MOESM5_ESM.zip › FIG4/FIG.4A/SR DIA WT 12m X20 6.tiff]

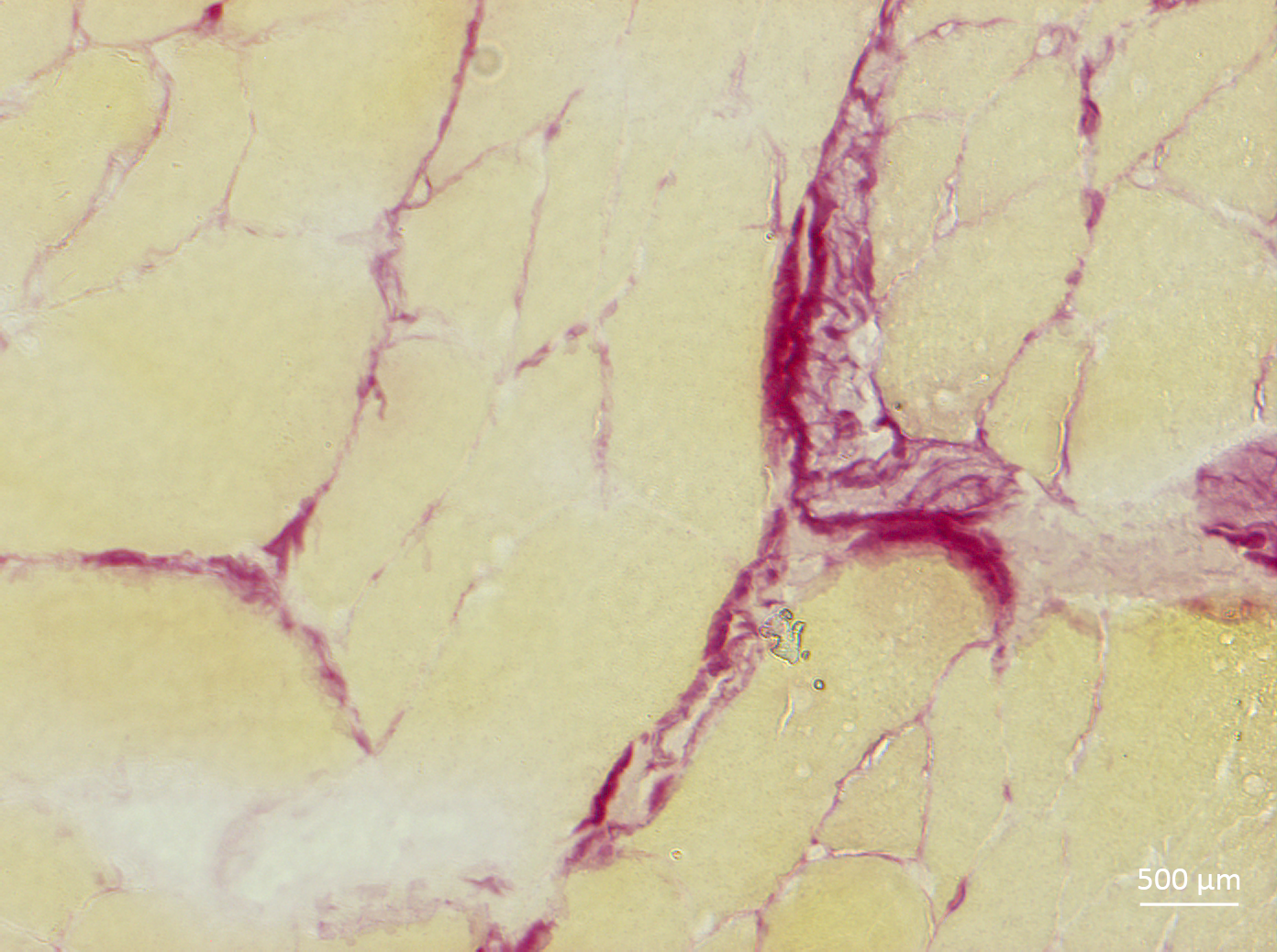

Supplement: Supplementary file 5 — Source data Fig. 4 [file 44319_2024_249_MOESM5_ESM.zip › FIG4/FIG.4A/SR DIA BMD 12M.tif]

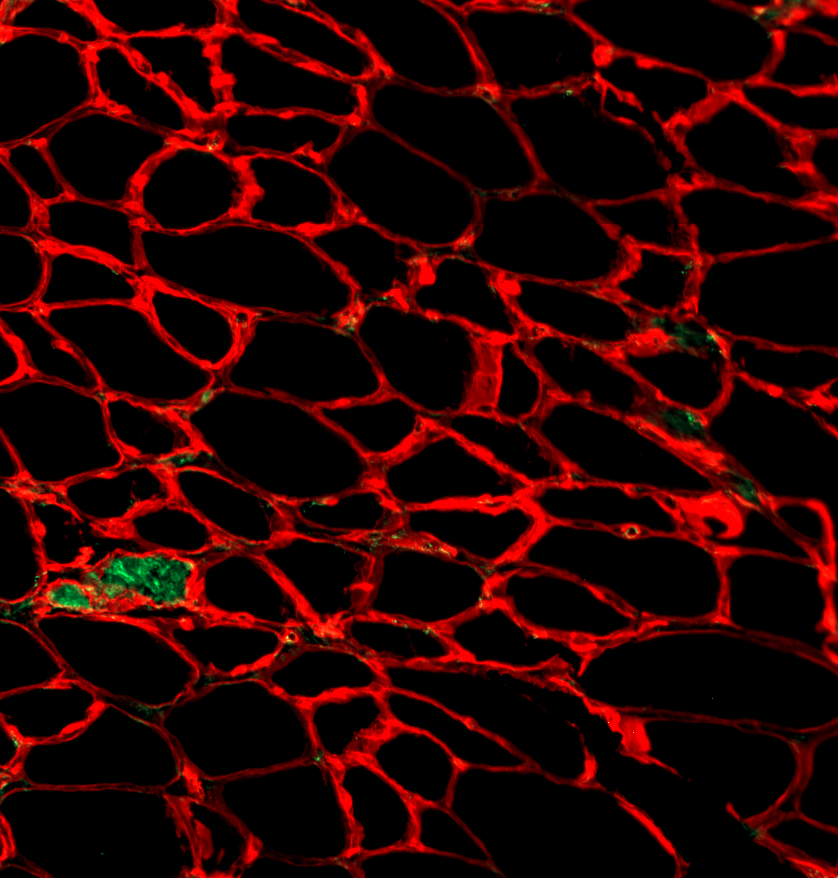

Supplement: Supplementary file 5 — Source data Fig. 4 [file 44319_2024_249_MOESM5_ESM.zip › FIG4/FIG.4C/BMD.tif]

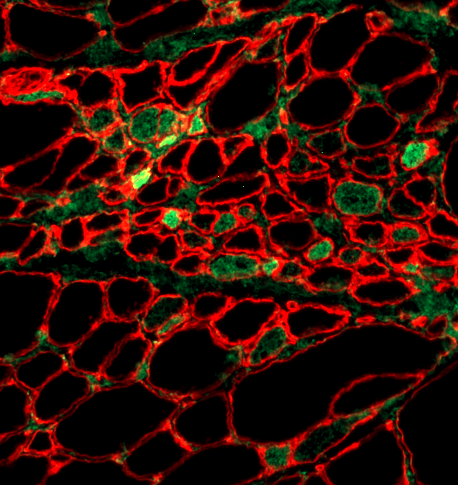

Supplement: Supplementary file 5 — Source data Fig. 4 [file 44319_2024_249_MOESM5_ESM.zip › FIG4/FIG.4C/DMD.tif]

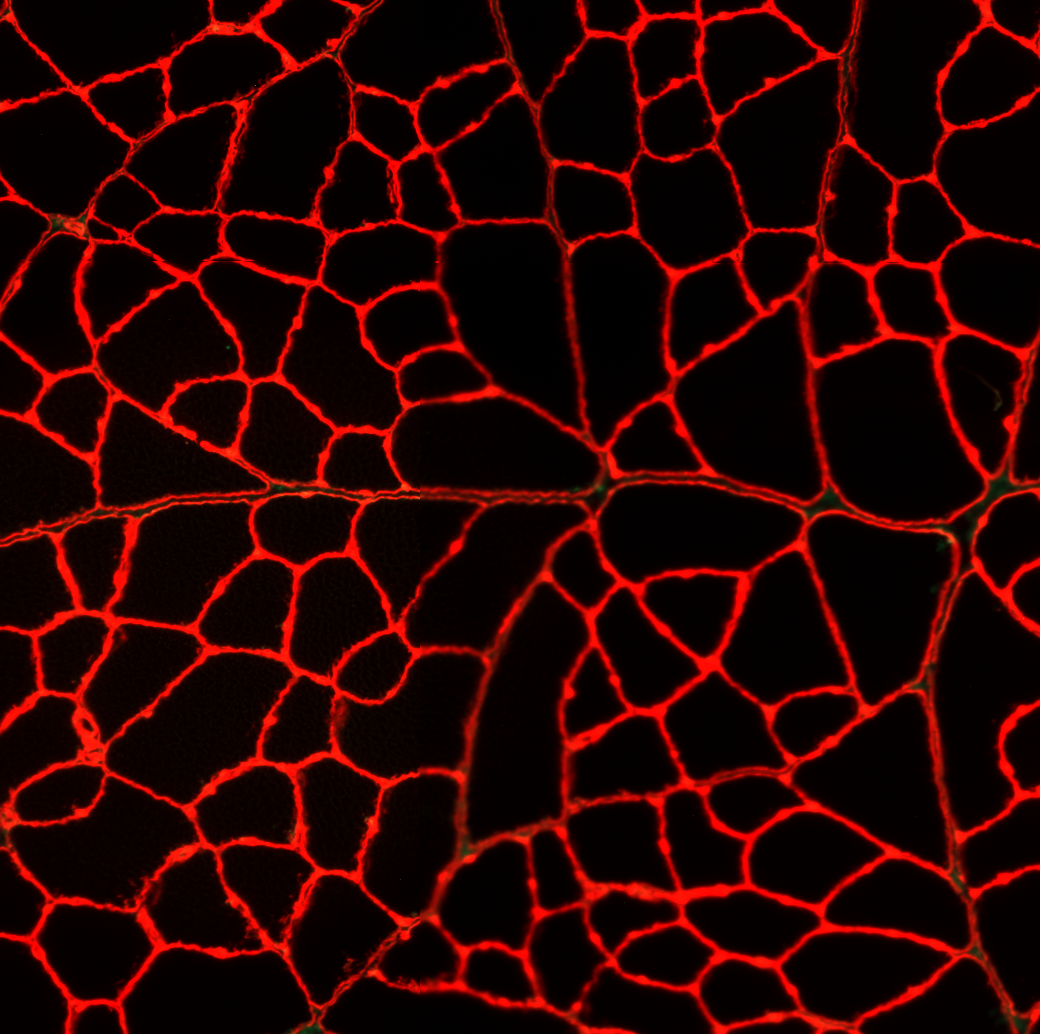

Supplement: Supplementary file 5 — Source data Fig. 4 [file 44319_2024_249_MOESM5_ESM.zip › FIG4/FIG.4C/WT.tif]

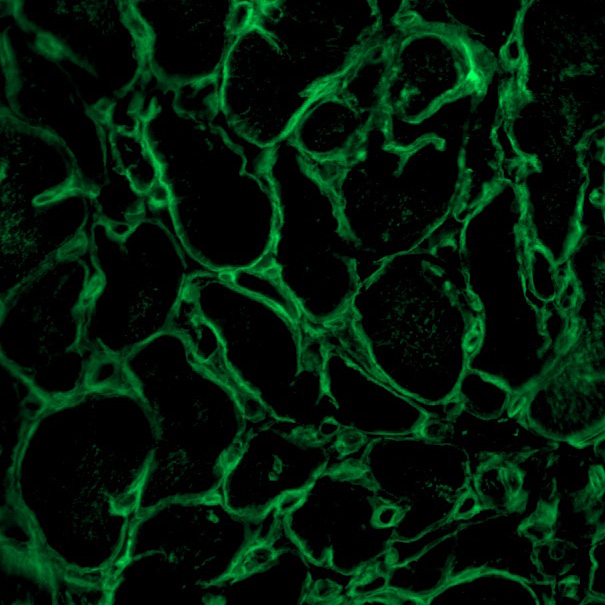

Supplement: Supplementary file 6 — Source data Fig. 5 [file 44319_2024_249_MOESM6_ESM.zip › FIG5/FIG.5C/BMD 11M 22 3.jpg]

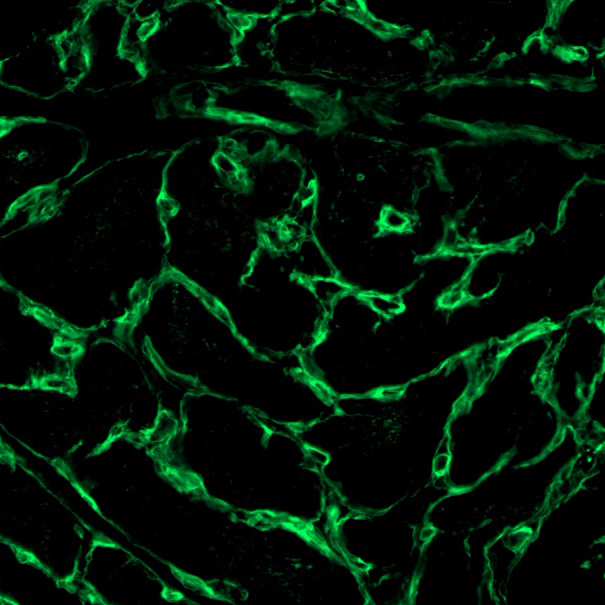

Supplement: Supplementary file 6 — Source data Fig. 5 [file 44319_2024_249_MOESM6_ESM.zip › FIG5/FIG.5C/DMD 11M 196.jpg]

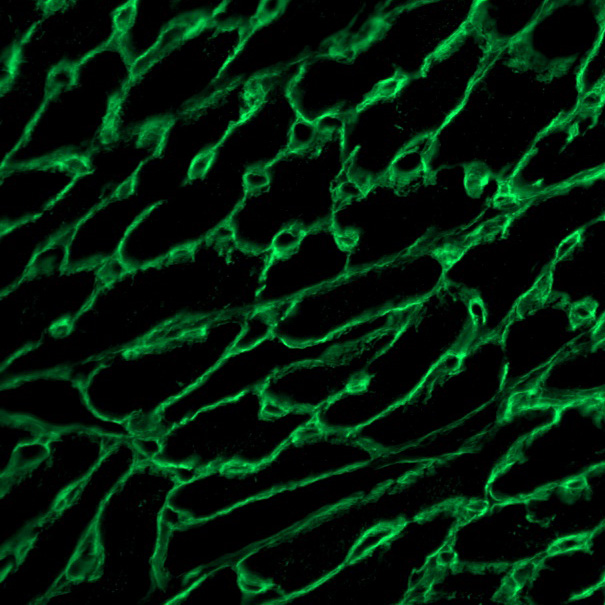

Supplement: Supplementary file 6 — Source data Fig. 5 [file 44319_2024_249_MOESM6_ESM.zip › FIG5/FIG.5C/WT 11M 343 2_.jpg]

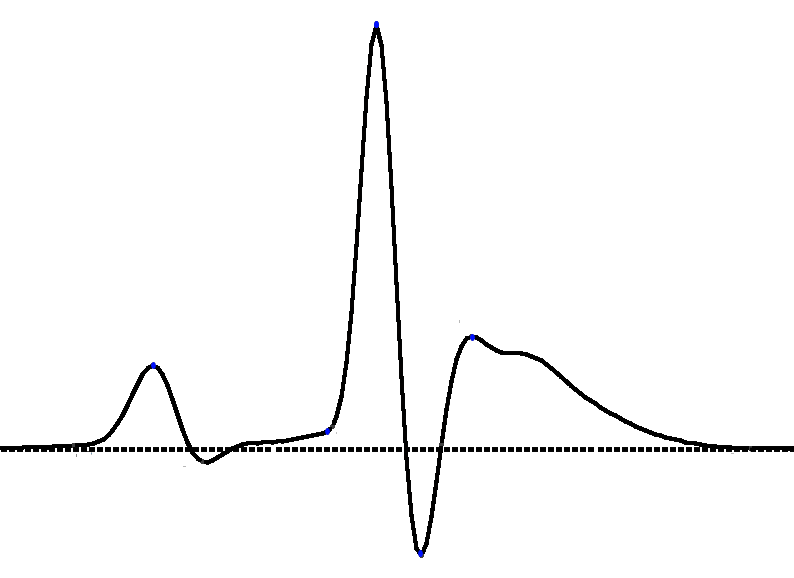

Supplement: Supplementary file 6 — Source data Fig. 5 [file 44319_2024_249_MOESM6_ESM.zip › FIG5/FIG.5E/WT-662-b-BASAL.tiff]

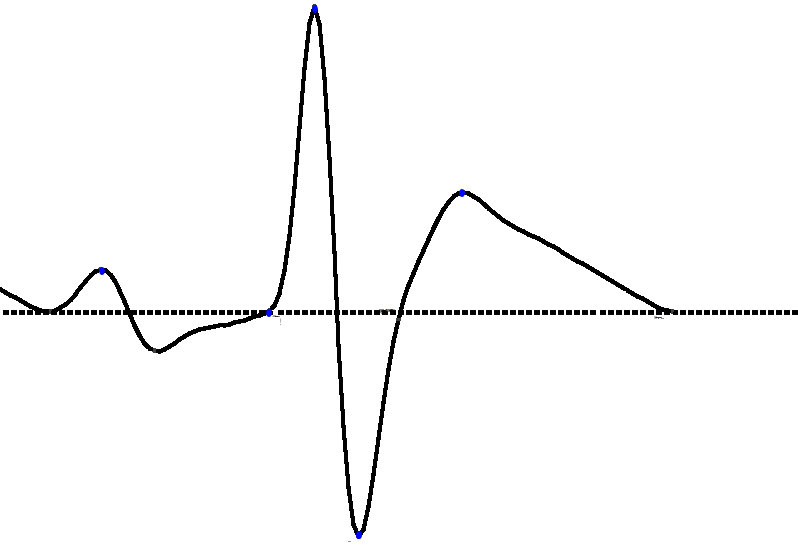

Supplement: Supplementary file 6 — Source data Fig. 5 [file 44319_2024_249_MOESM6_ESM.zip › FIG5/FIG.5E/WT-662-10-ISO.tiff]

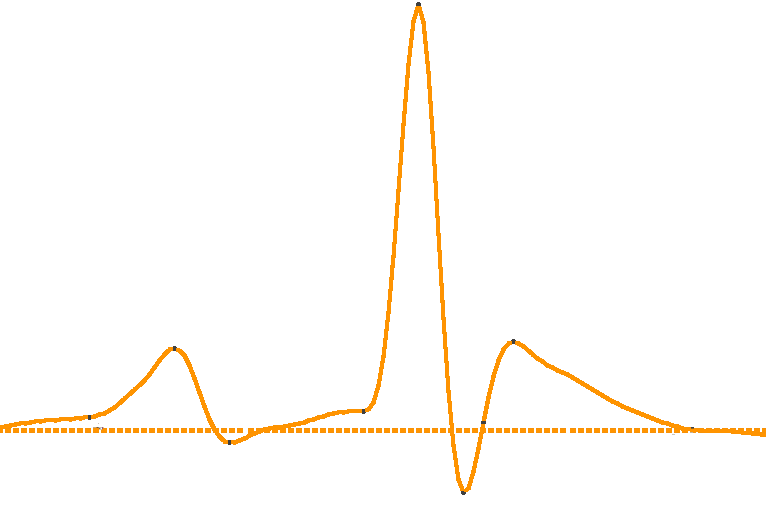

Supplement: Supplementary file 6 — Source data Fig. 5 [file 44319_2024_249_MOESM6_ESM.zip › FIG5/FIG.5E/BMD-865-b-vt-BASAL.tiff]

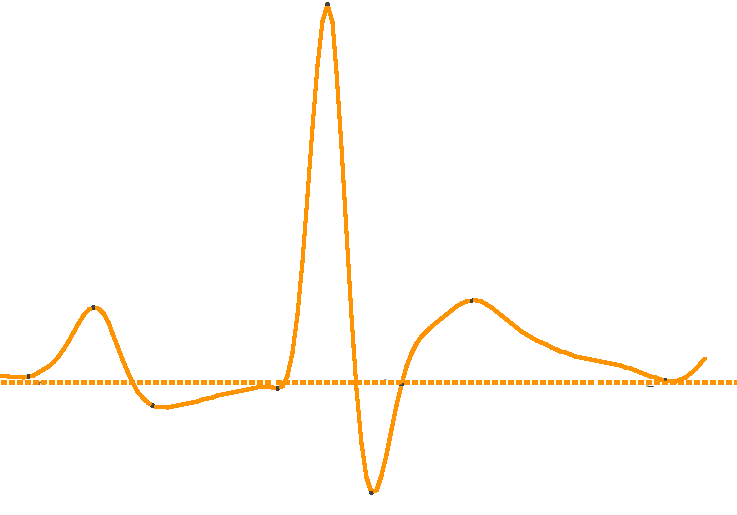

Supplement: Supplementary file 6 — Source data Fig. 5 [file 44319_2024_249_MOESM6_ESM.zip › FIG5/FIG.5E/BMD-865-10-ISO.tiff]

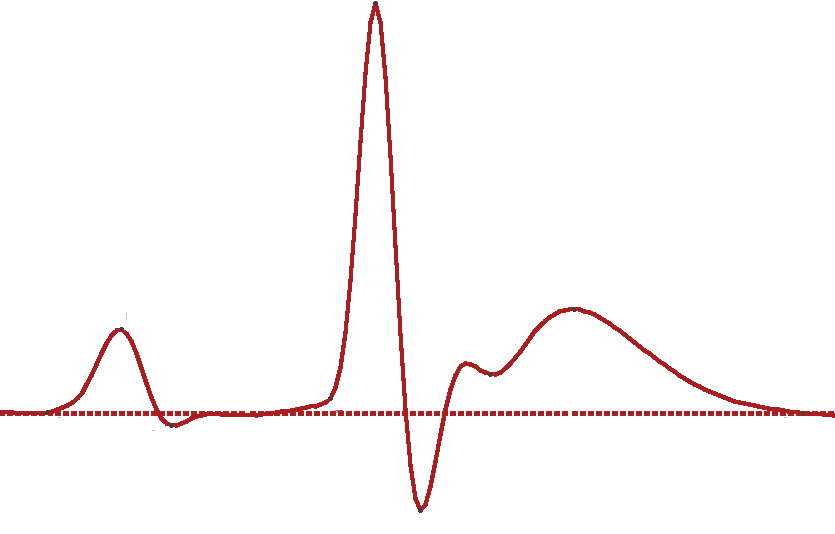

Supplement: Supplementary file 6 — Source data Fig. 5 [file 44319_2024_249_MOESM6_ESM.zip › FIG5/FIG.5E/DMD-741-b-BASAL.tiff]

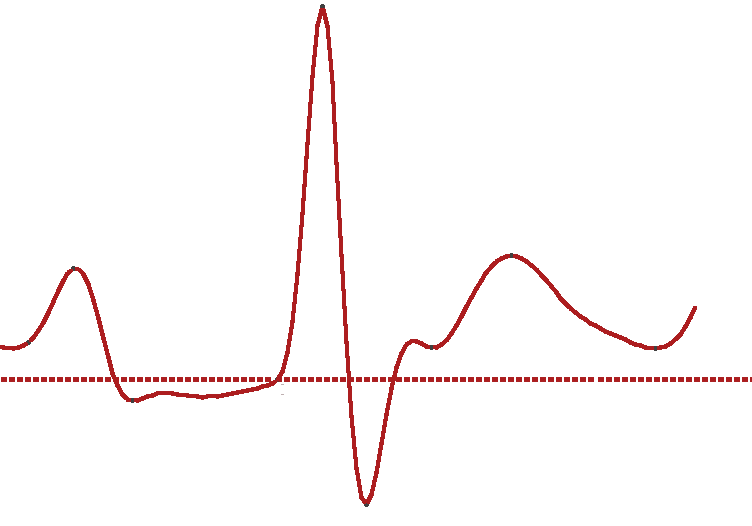

Supplement: Supplementary file 6 — Source data Fig. 5 [file 44319_2024_249_MOESM6_ESM.zip › FIG5/FIG.5E/DMD-741-10_ISO.tiff]

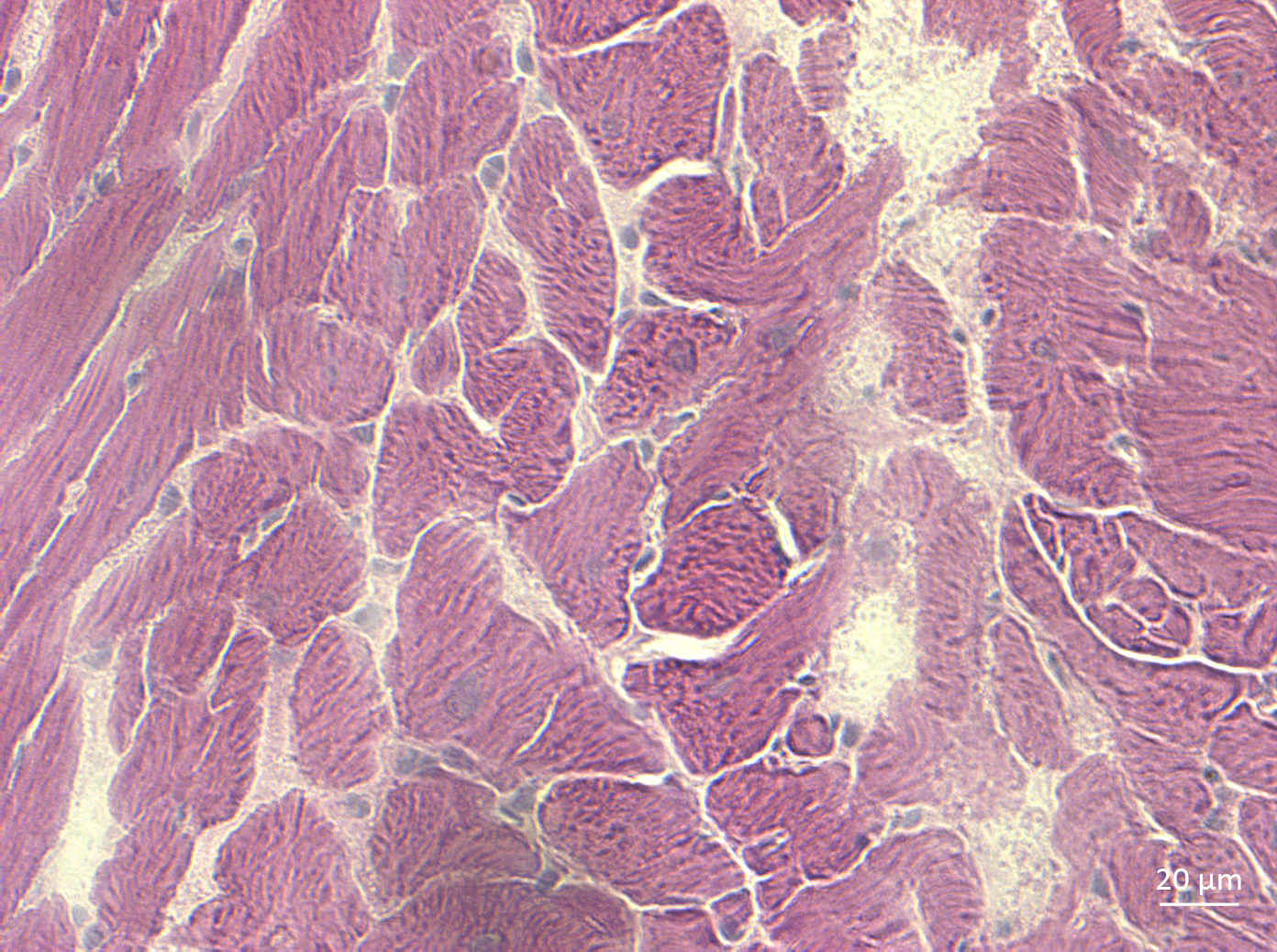

Supplement: Supplementary file 6 — Source data Fig. 5 [file 44319_2024_249_MOESM6_ESM.zip › FIG5/FIG.5A/HE HEART BMD 6M.tif]

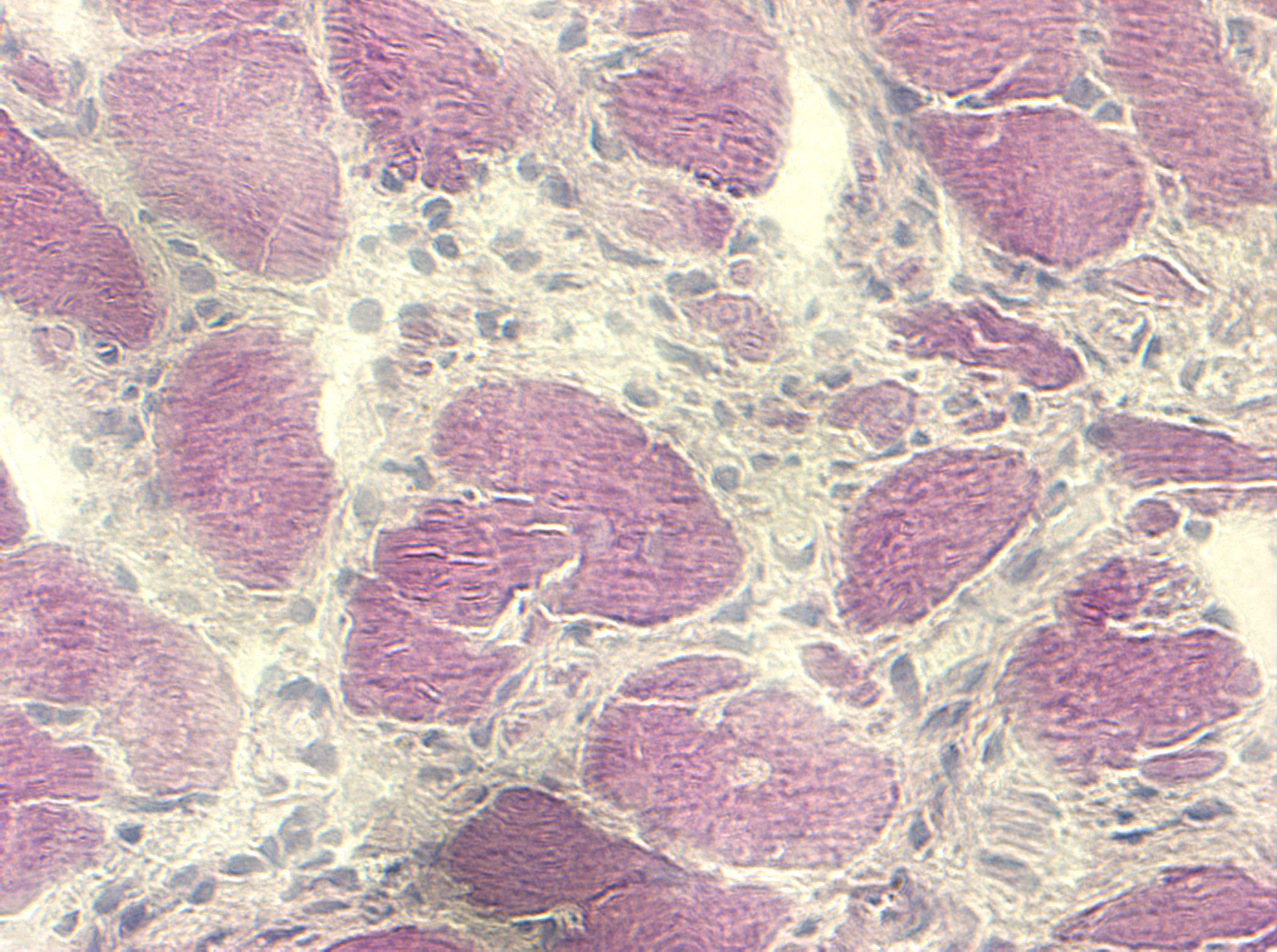

Supplement: Supplementary file 6 — Source data Fig. 5 [file 44319_2024_249_MOESM6_ESM.zip › FIG5/FIG.5A/HE HEART BMD 12M.tif]

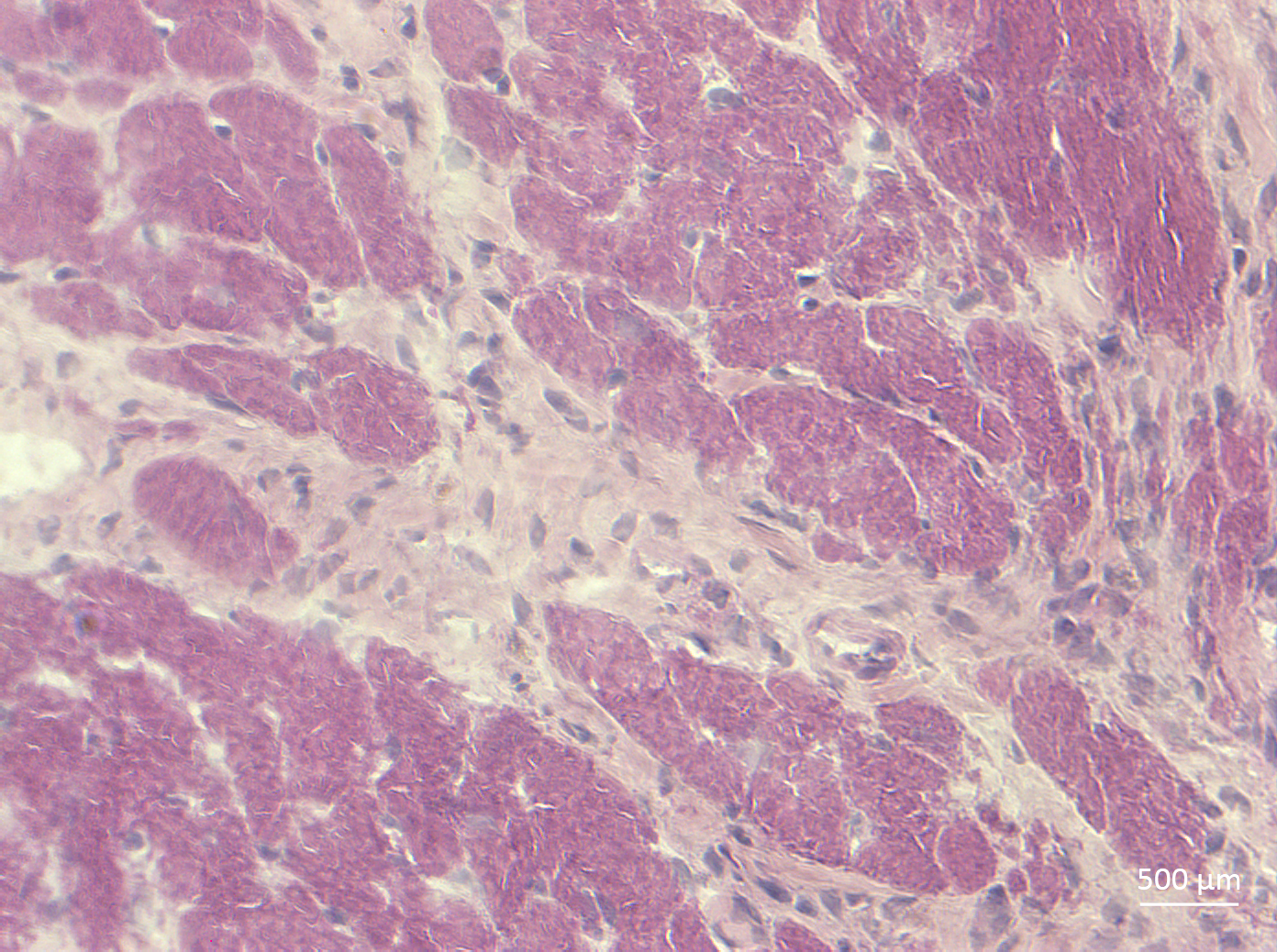

Supplement: Supplementary file 6 — Source data Fig. 5 [file 44319_2024_249_MOESM6_ESM.zip › FIG5/FIG.5A/HE HEART DMD 6M.tif]

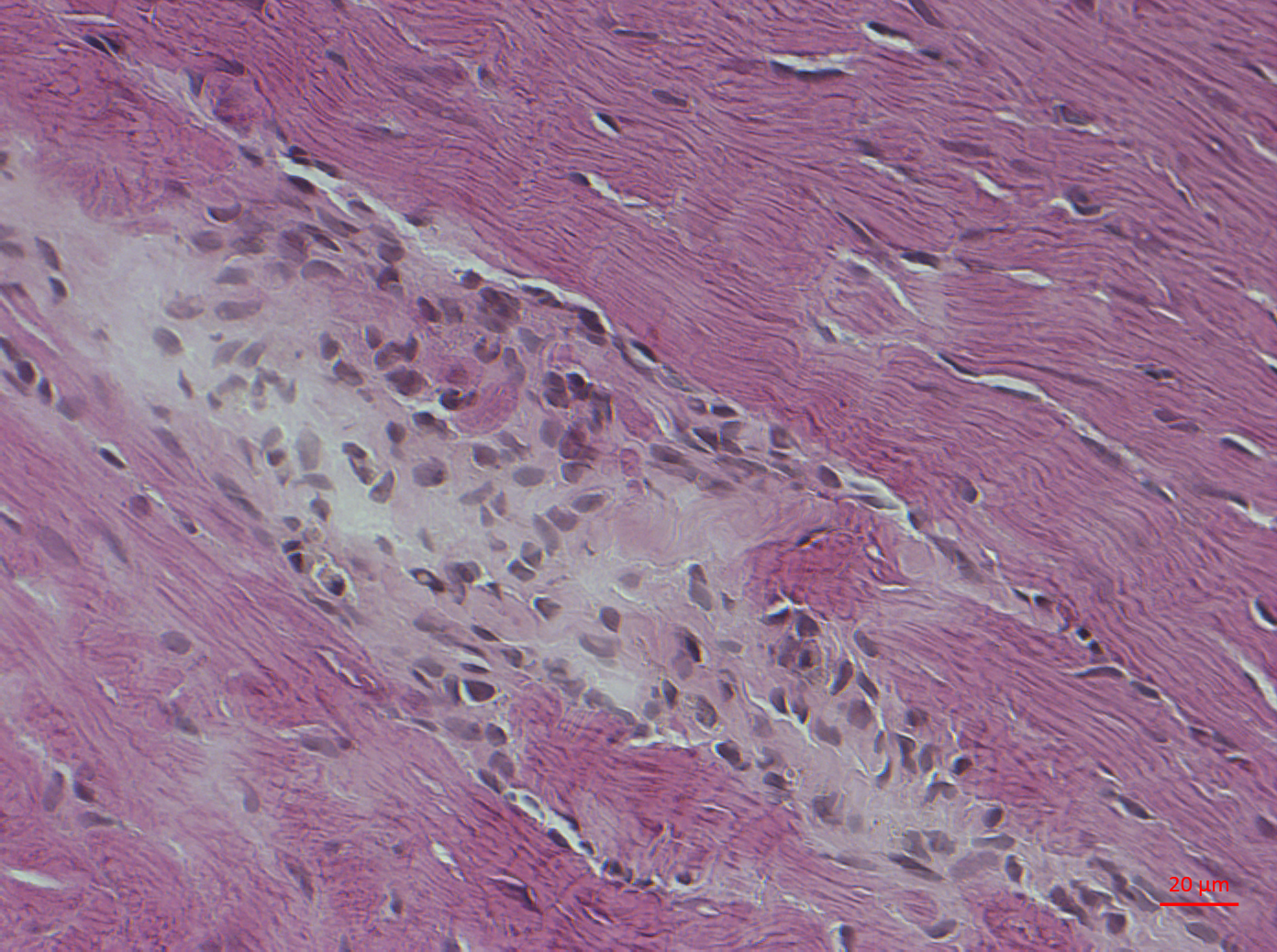

Supplement: Supplementary file 6 — Source data Fig. 5 [file 44319_2024_249_MOESM6_ESM.zip › FIG5/FIG.5A/HE HEART DMD 12M.tif]

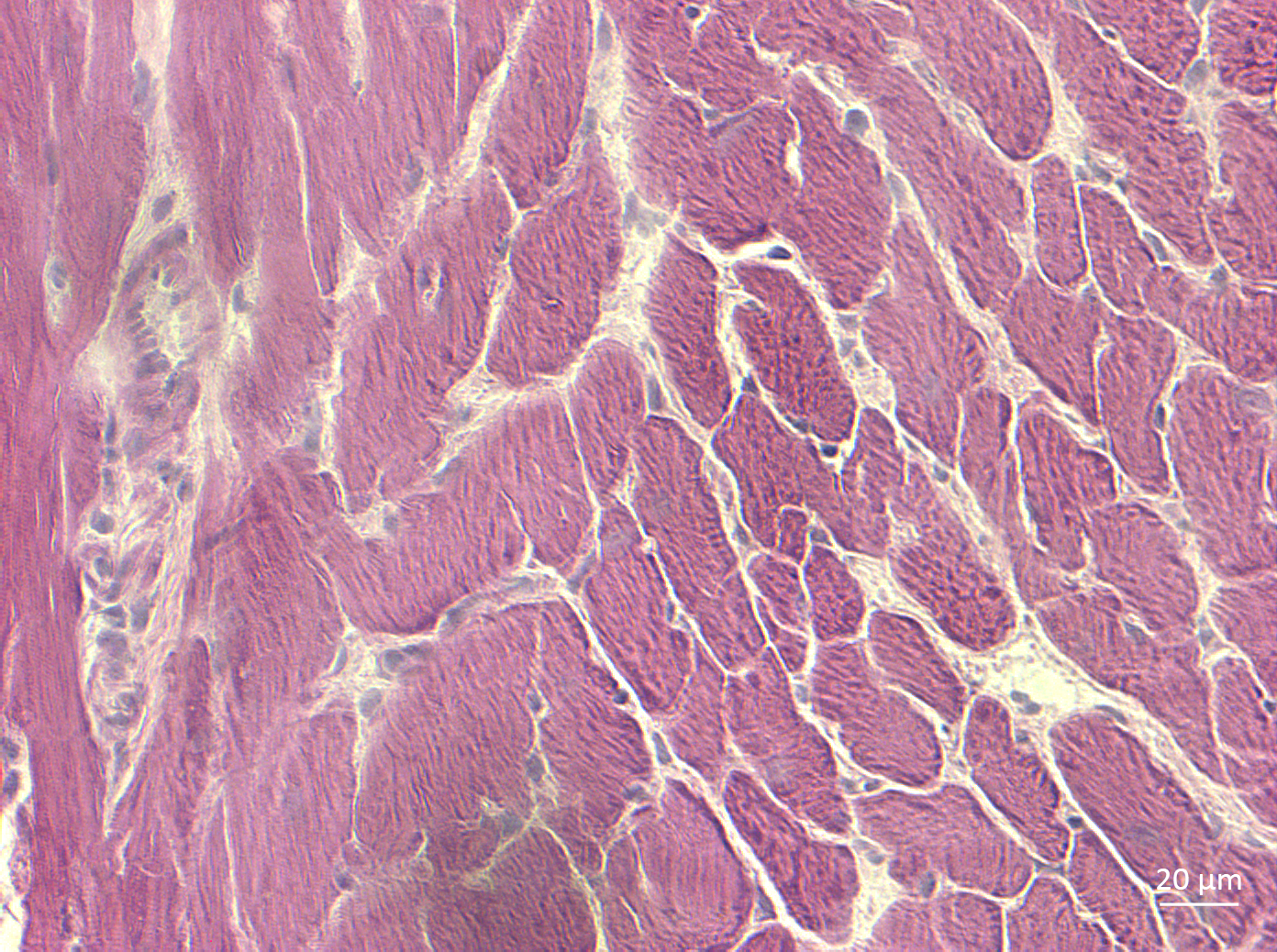

Supplement: Supplementary file 6 — Source data Fig. 5 [file 44319_2024_249_MOESM6_ESM.zip › FIG5/FIG.5A/HE HEART WT 6M.tif]

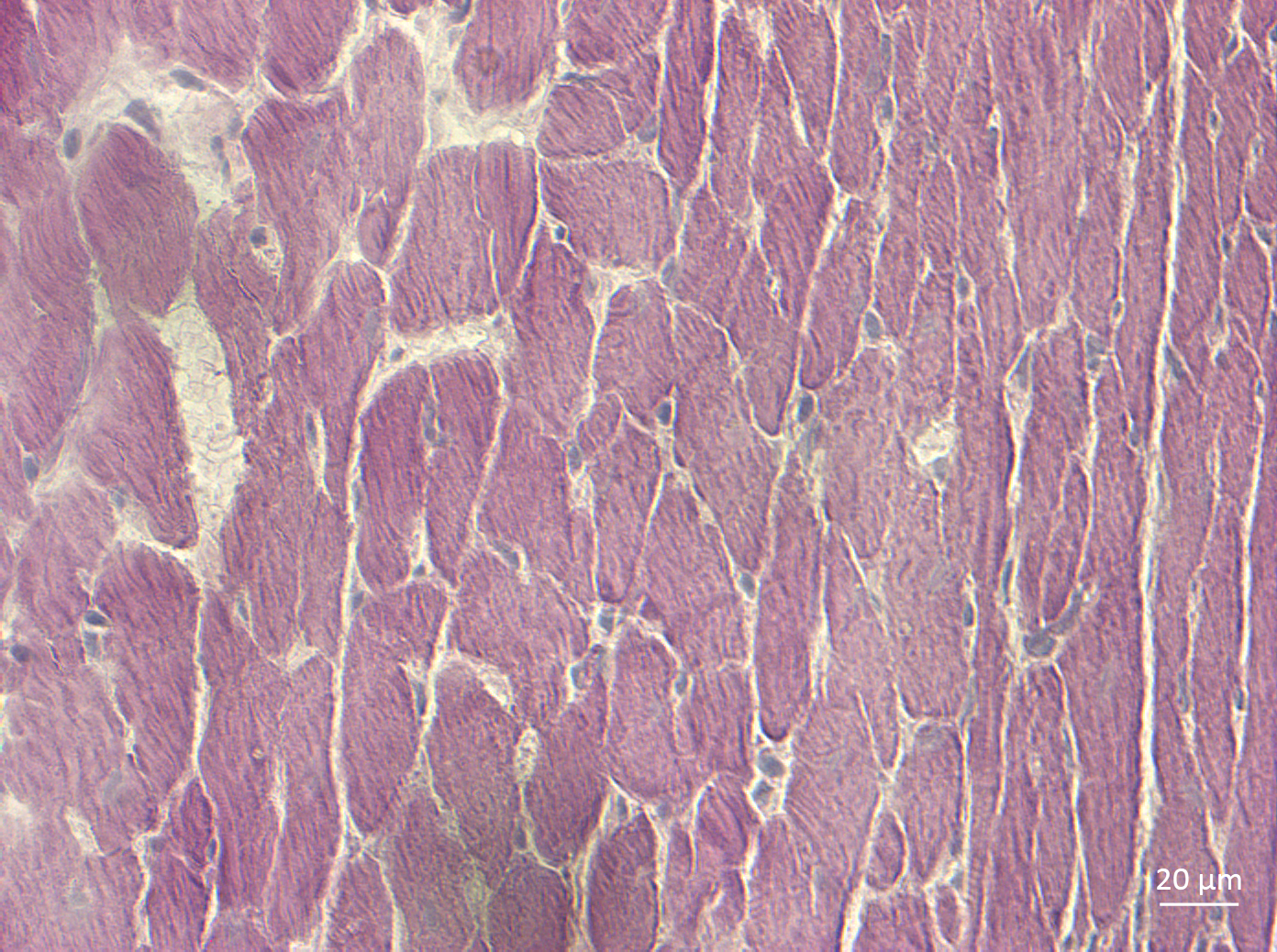

Supplement: Supplementary file 6 — Source data Fig. 5 [file 44319_2024_249_MOESM6_ESM.zip › FIG5/FIG.5A/HE HEART WT 12M .tif]

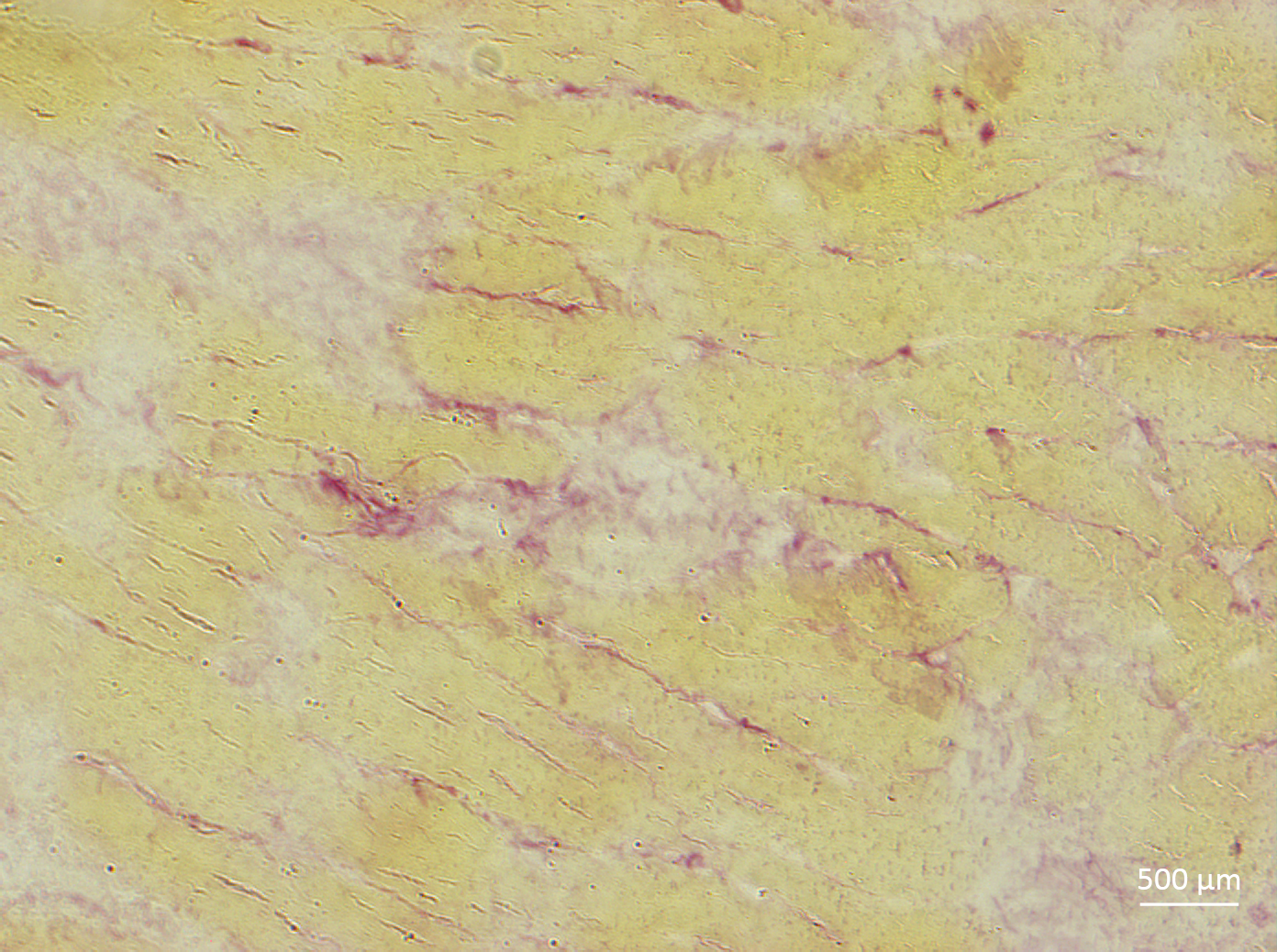

Supplement: Supplementary file 6 — Source data Fig. 5 [file 44319_2024_249_MOESM6_ESM.zip › FIG5/FIG.5A/SR HEART BMD 6M.tif]

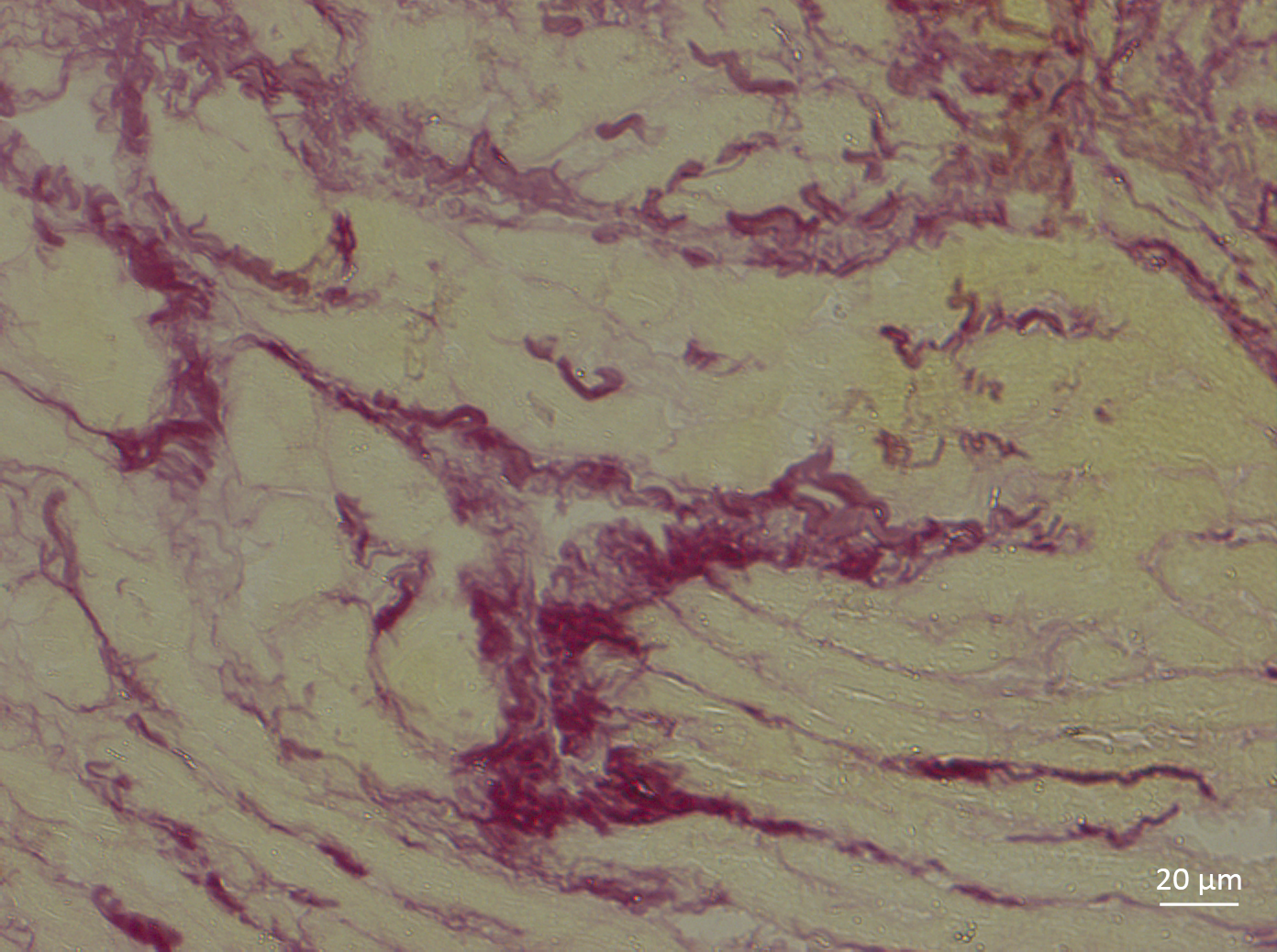

Supplement: Supplementary file 6 — Source data Fig. 5 [file 44319_2024_249_MOESM6_ESM.zip › FIG5/FIG.5A/SR HEART BMD 12M.tif]

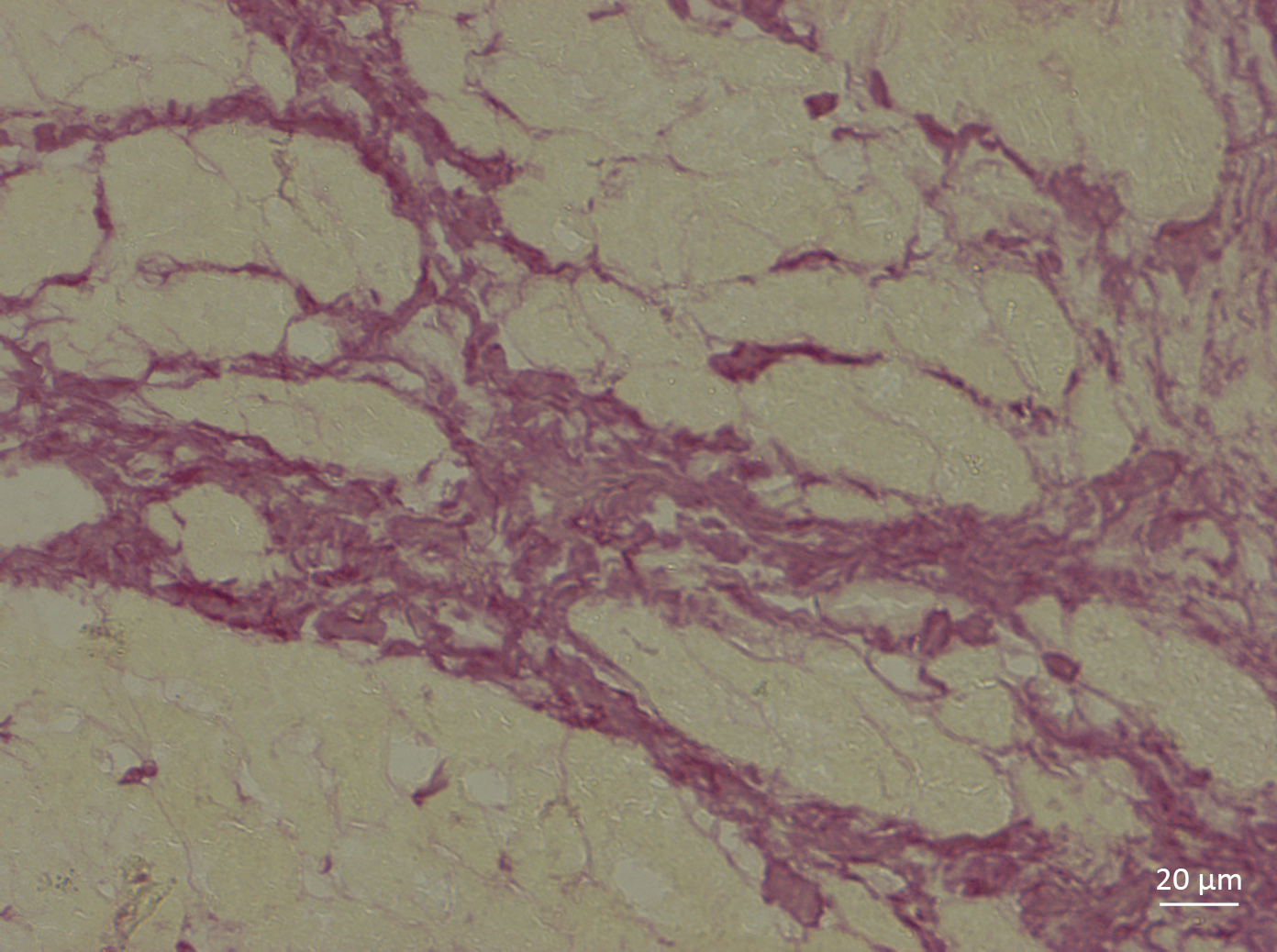

Supplement: Supplementary file 6 — Source data Fig. 5 [file 44319_2024_249_MOESM6_ESM.zip › FIG5/FIG.5A/SR HEART DMD 6M.tif]

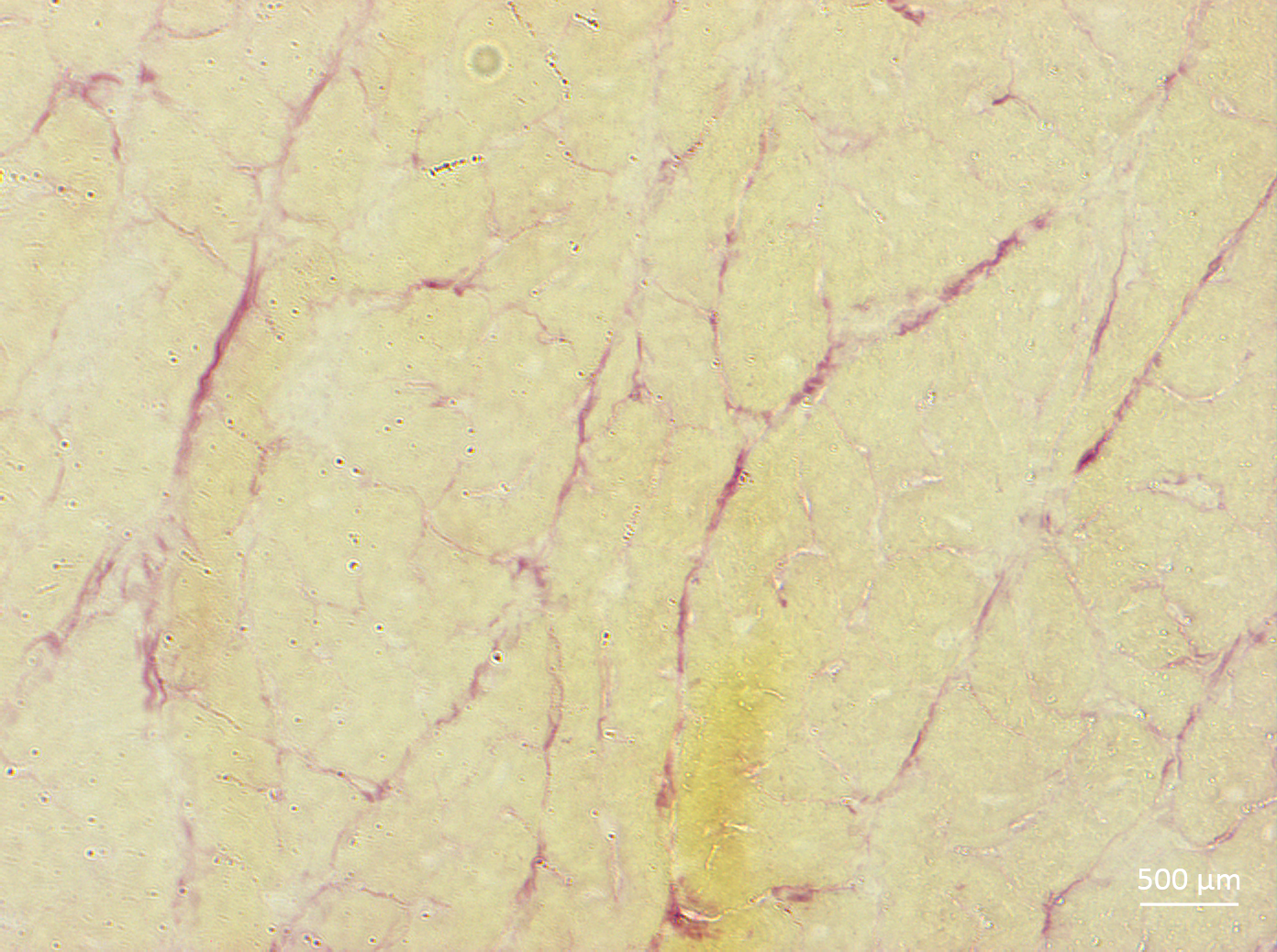

Supplement: Supplementary file 6 — Source data Fig. 5 [file 44319_2024_249_MOESM6_ESM.zip › FIG5/FIG.5A/SR HEART WT 6M.tif]

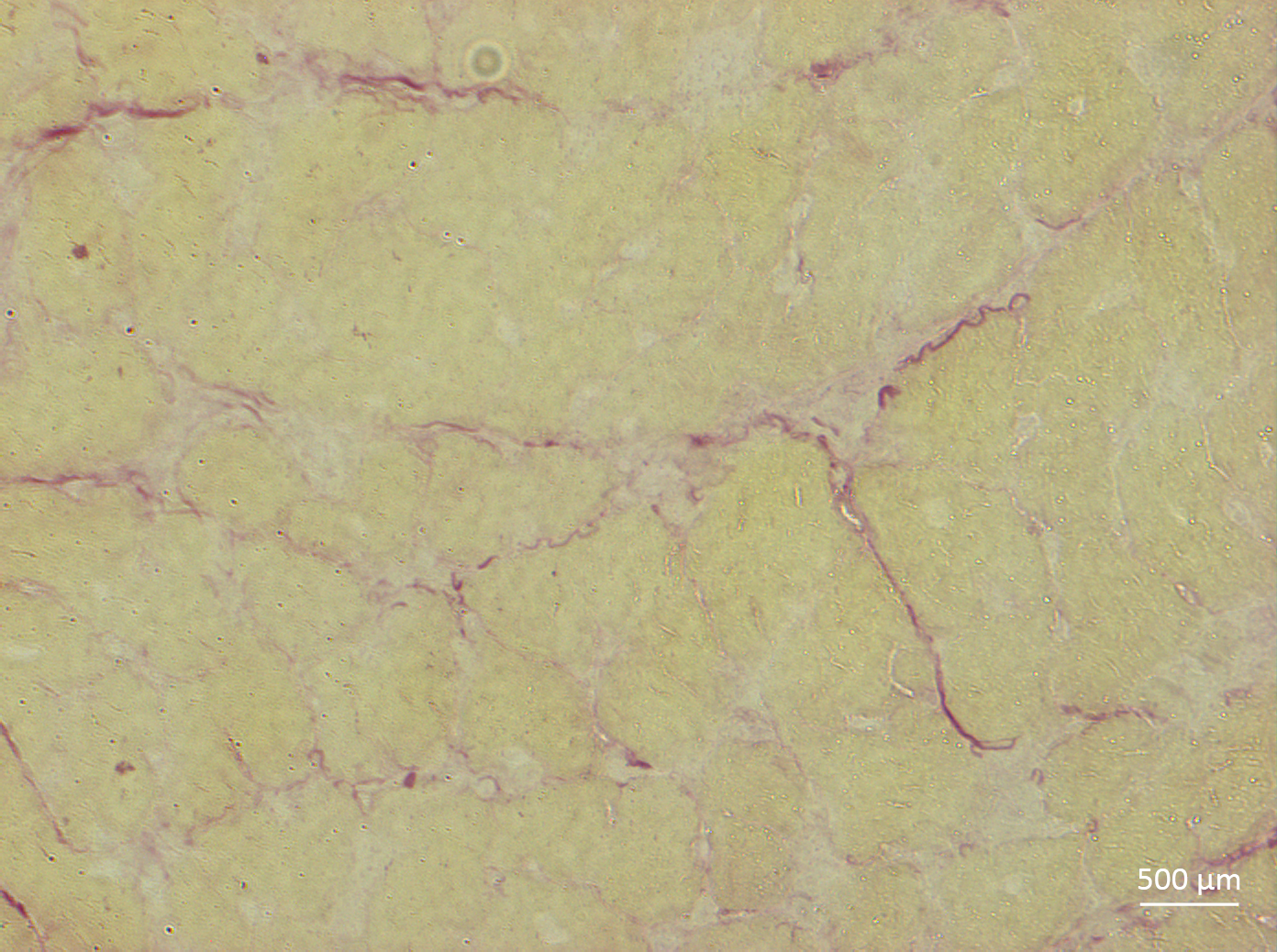

Supplement: Supplementary file 6 — Source data Fig. 5 [file 44319_2024_249_MOESM6_ESM.zip › FIG5/FIG.5A/SR HEART WT 12M.tif]

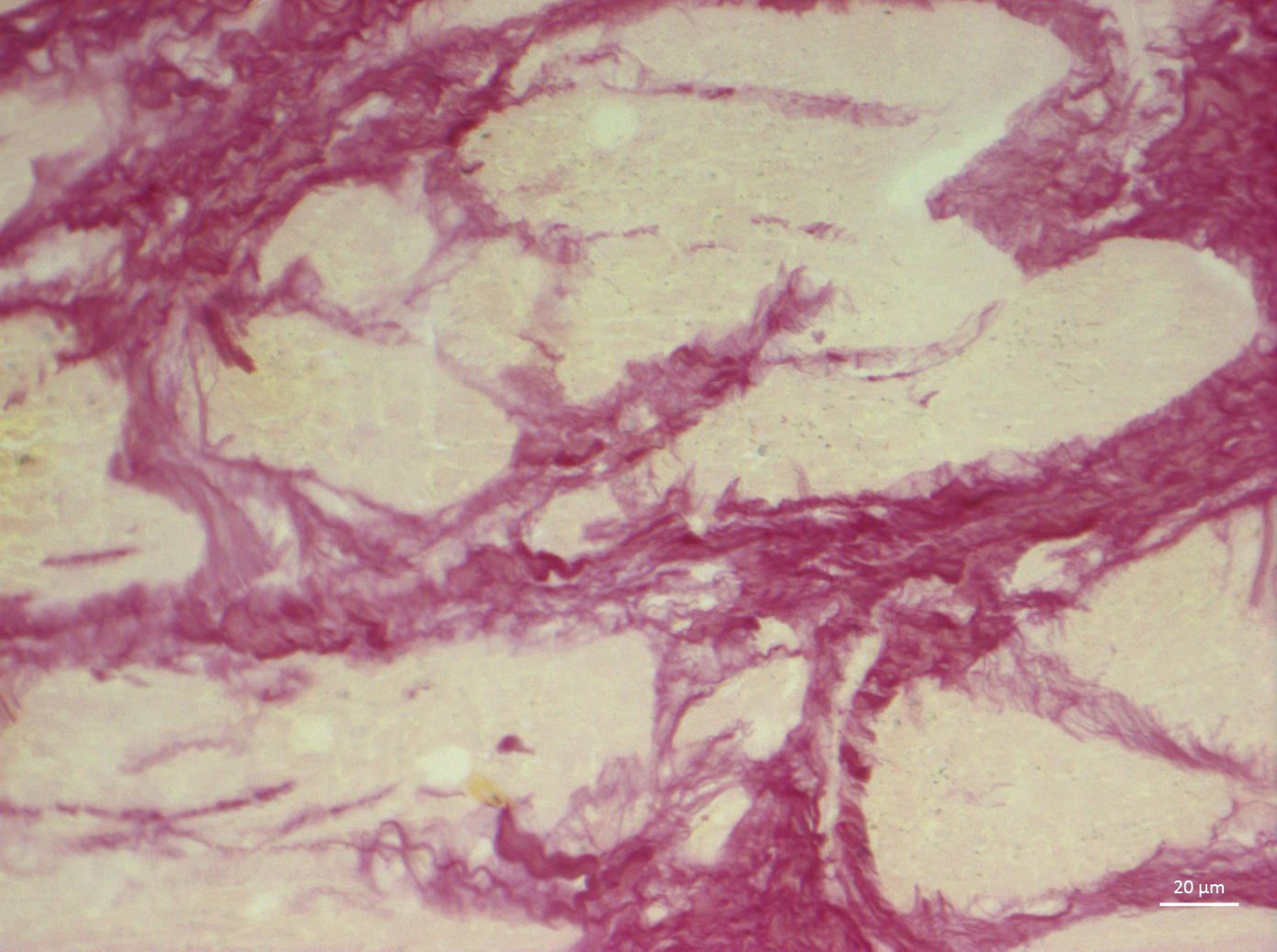

Supplement: Supplementary file 6 — Source data Fig. 5 [file 44319_2024_249_MOESM6_ESM.zip › FIG5/FIG.5A/SR HEART DMD 12M.tif]

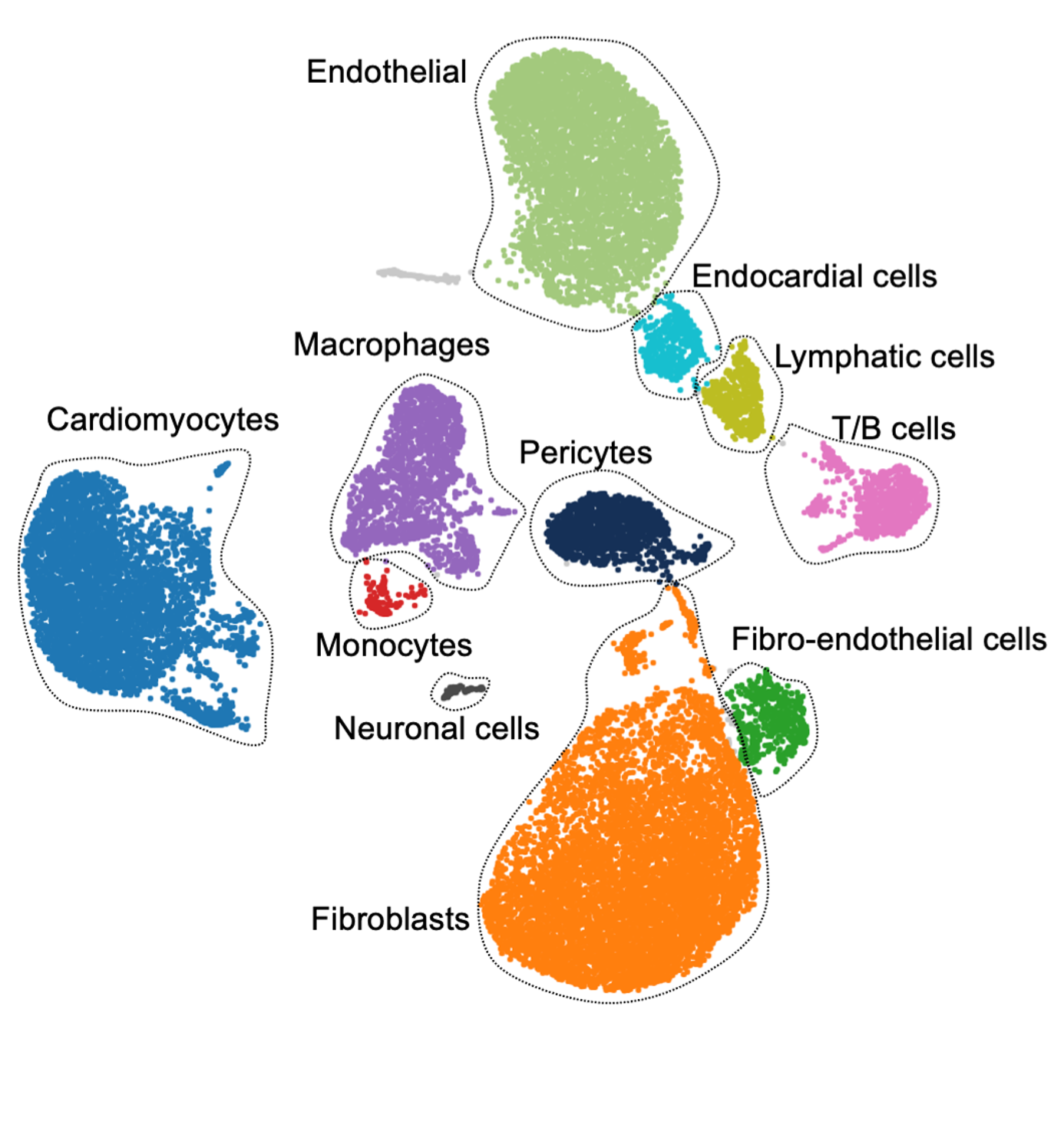

Supplement: Supplementary file 7 — Source data Fig. 6 [file 44319_2024_249_MOESM7_ESM.zip › FIG6/FIG.6A.tiff]

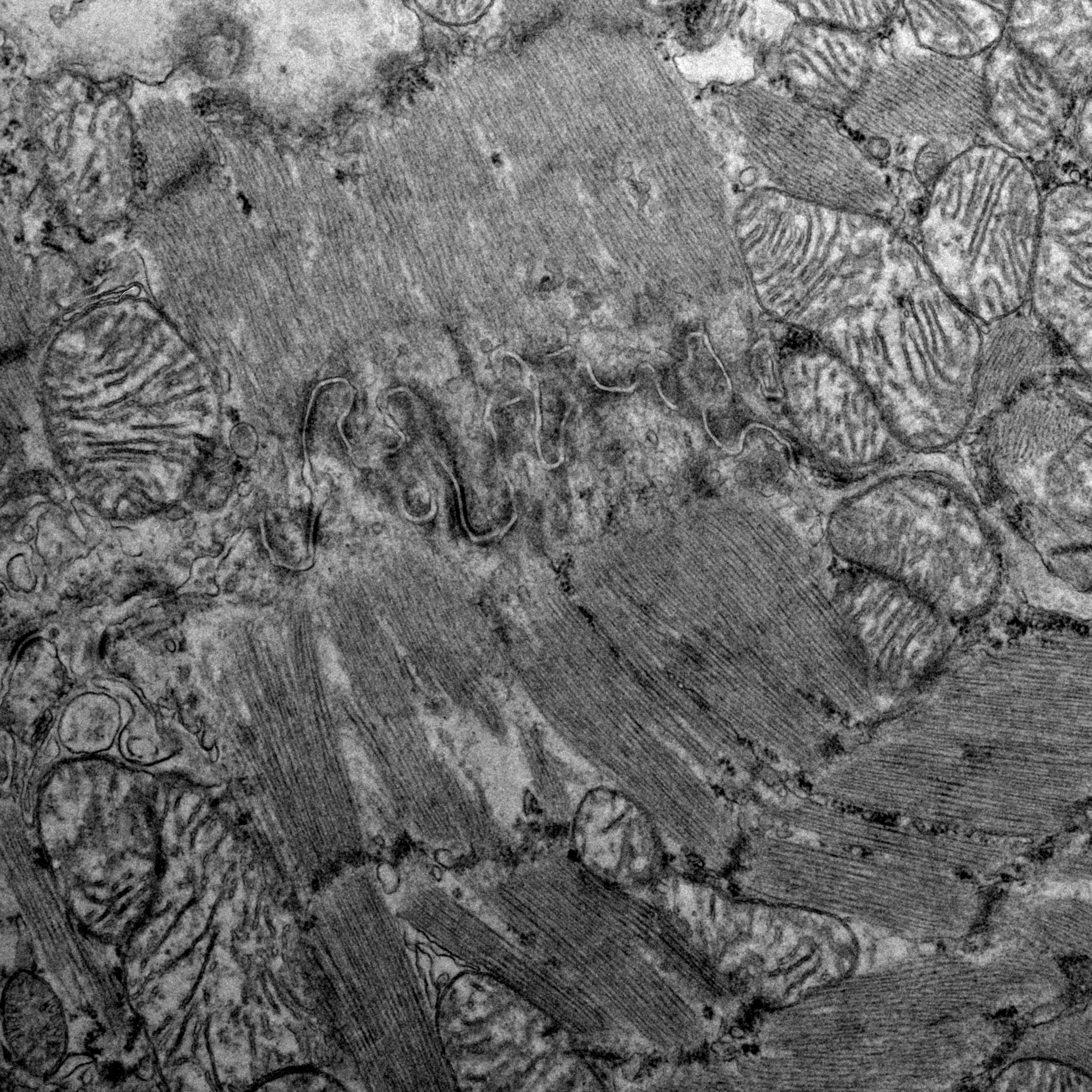

Supplement: Supplementary file 7 — Source data Fig. 6 [file 44319_2024_249_MOESM7_ESM.zip › FIG6/FIG.6J/1DMD-3--4800X-0013.tiff]

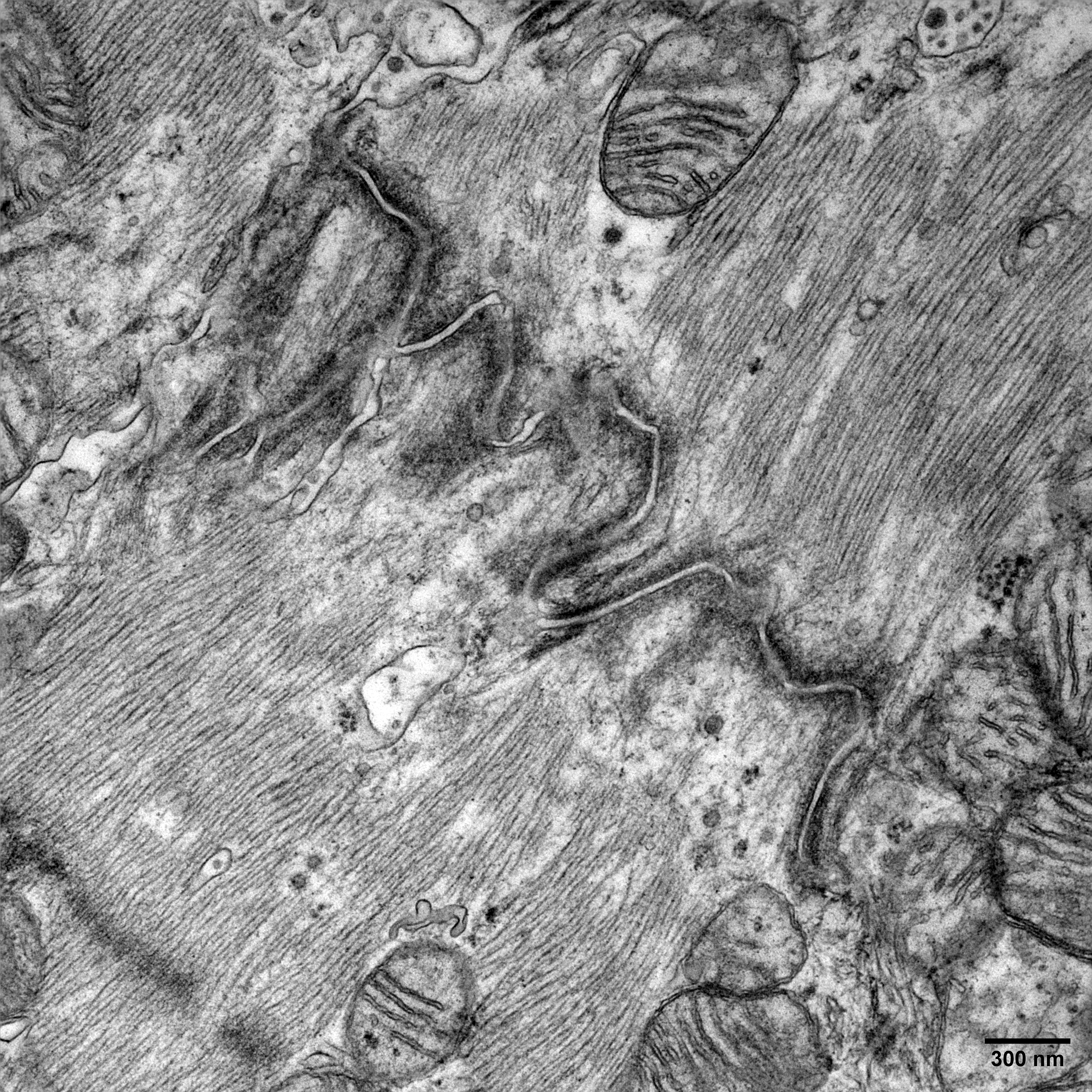

Supplement: Supplementary file 7 — Source data Fig. 6 [file 44319_2024_249_MOESM7_ESM.zip › FIG6/FIG.6J/1DMD-11kX-0035.tif]
