# Supplementary figures and images for: Progressive cardiomyopathy with intercalated disc disorganization in a rat model of Becker dystrophy (part 2 of 2)
Source: EMBO Rep. 2024 Oct 2;25(11):4898–920. doi: 10.1038/s44319-024-00249-9 (PMC11549483; doi:10.1038/s44319-024-00249-9)

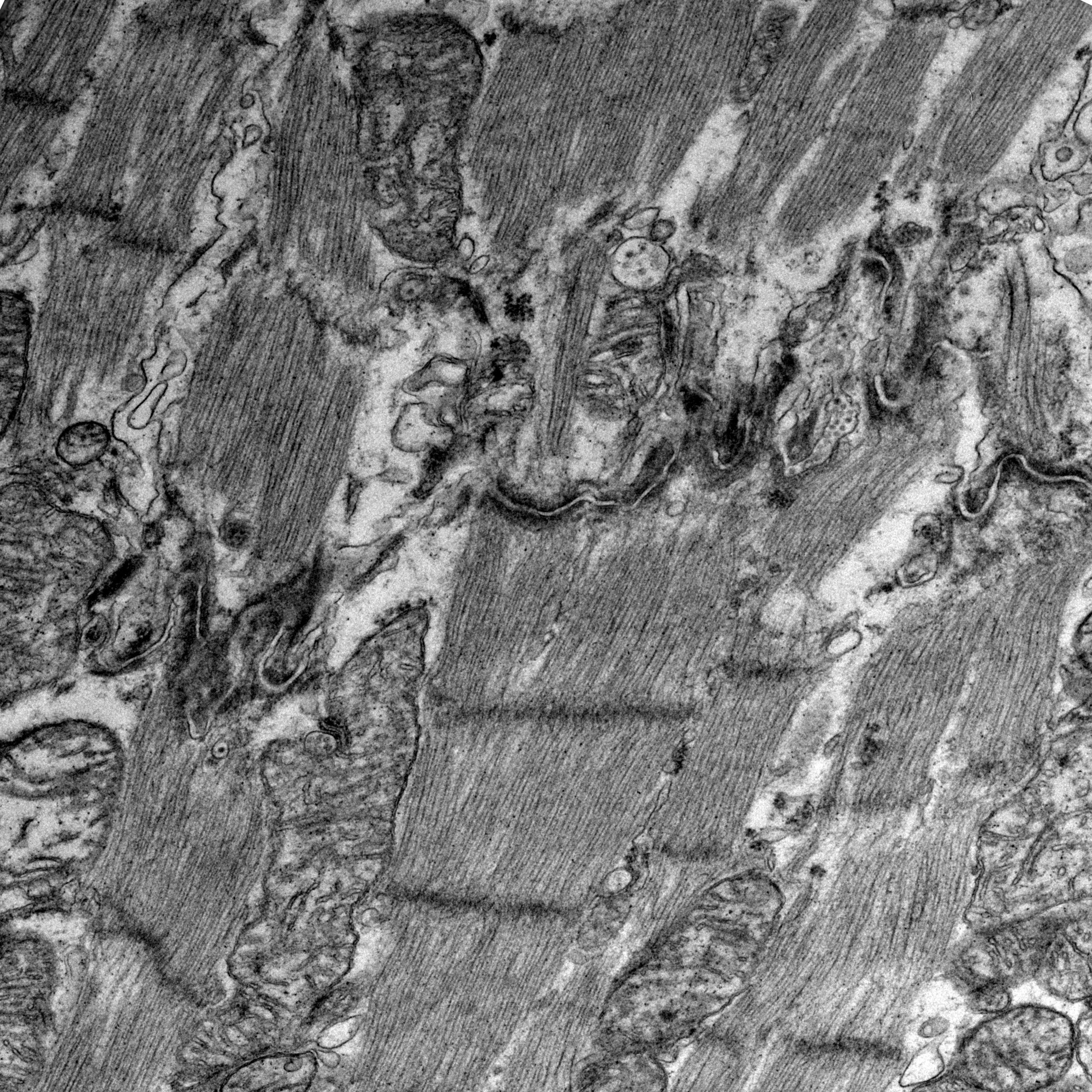

Supplement: Supplementary file 7 — Source data Fig. 6 [file 44319_2024_249_MOESM7_ESM.zip › FIG6/FIG.6J/2BMD-3--4800X-0013.tiff]

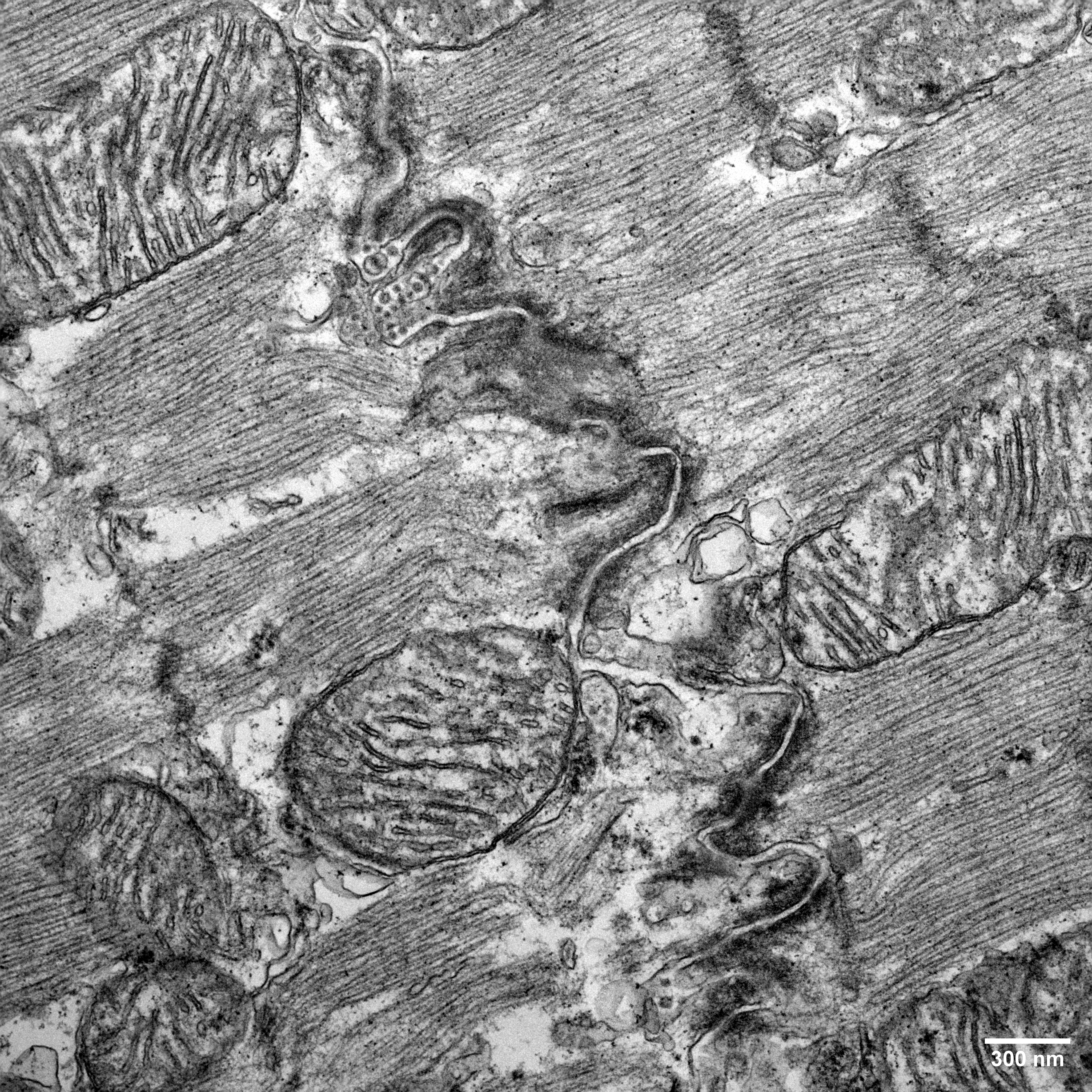

Supplement: Supplementary file 7 — Source data Fig. 6 [file 44319_2024_249_MOESM7_ESM.zip › FIG6/FIG.6J/2BMD-11kX-0005.tif]

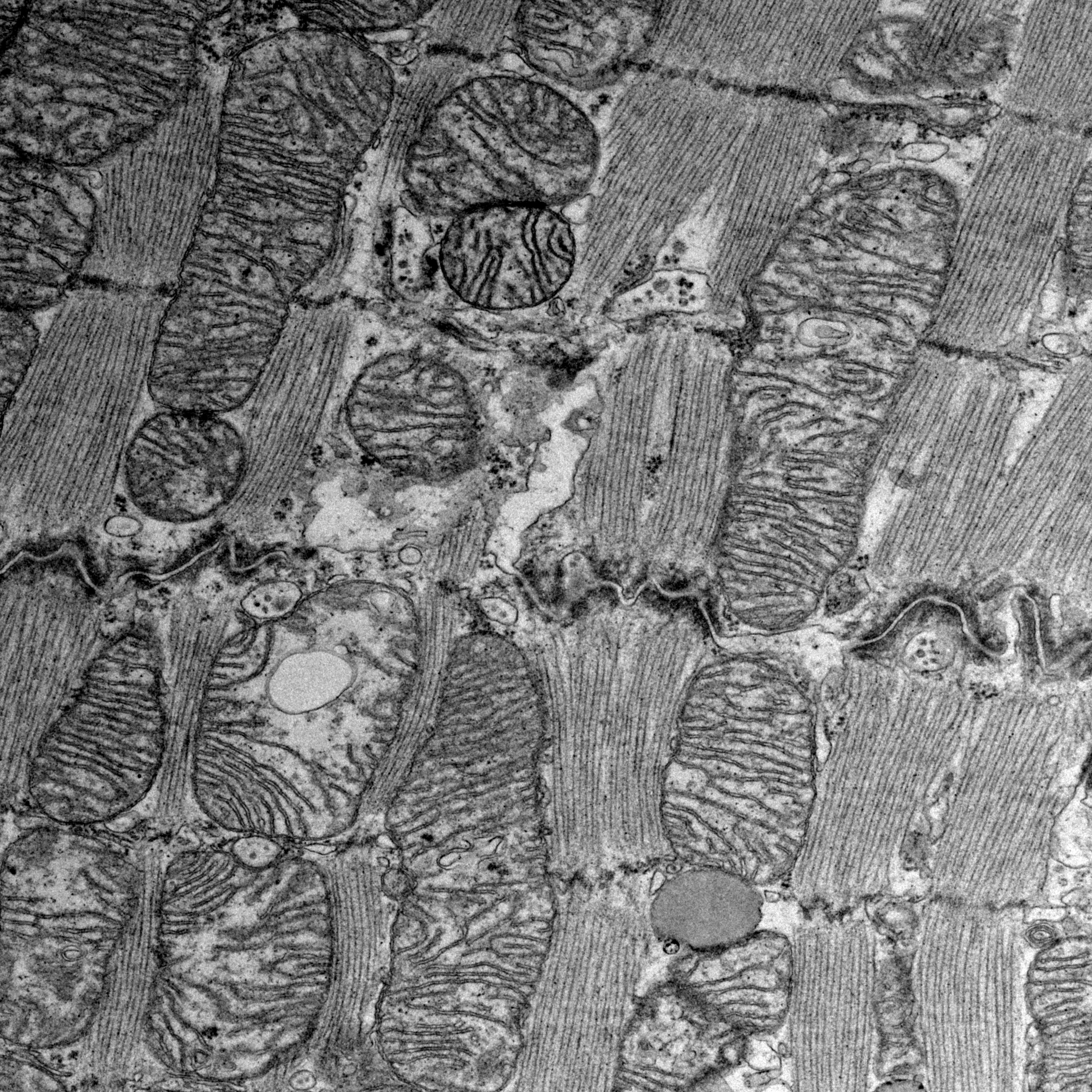

Supplement: Supplementary file 7 — Source data Fig. 6 [file 44319_2024_249_MOESM7_ESM.zip › FIG6/FIG.6J/2WT-3--4800X-0003.tiff]

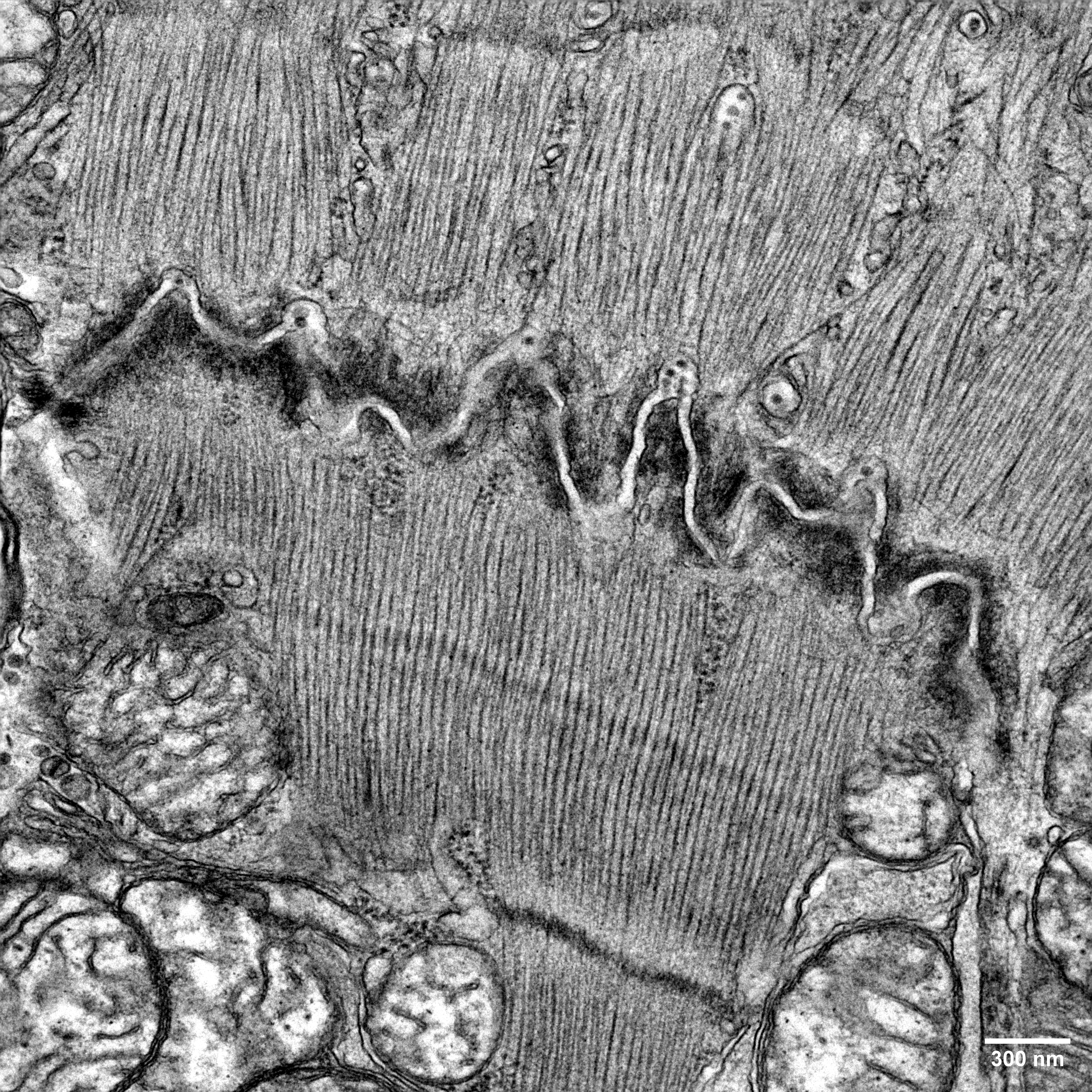

Supplement: Supplementary file 7 — Source data Fig. 6 [file 44319_2024_249_MOESM7_ESM.zip › FIG6/FIG.6J/2WT-11kX-0027.tif]

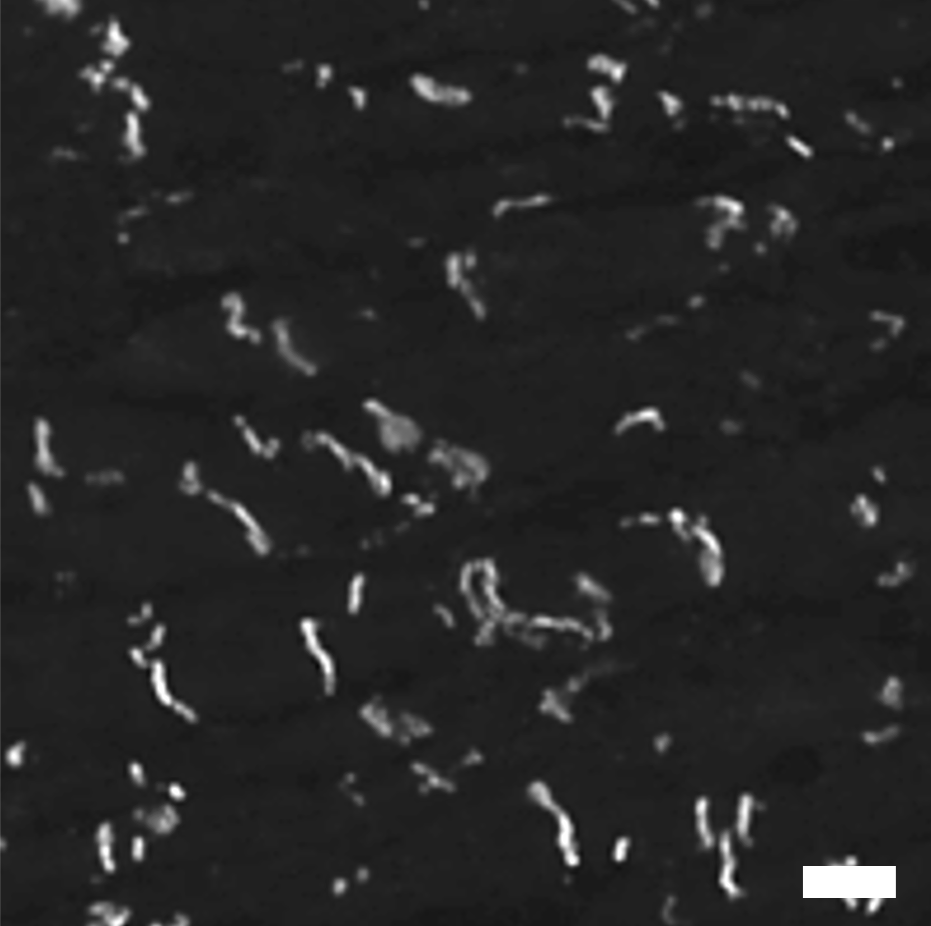

Supplement: Supplementary file 7 — Source data Fig. 6 [file 44319_2024_249_MOESM7_ESM.zip › FIG6/FIG.6D/BMD.tiff]

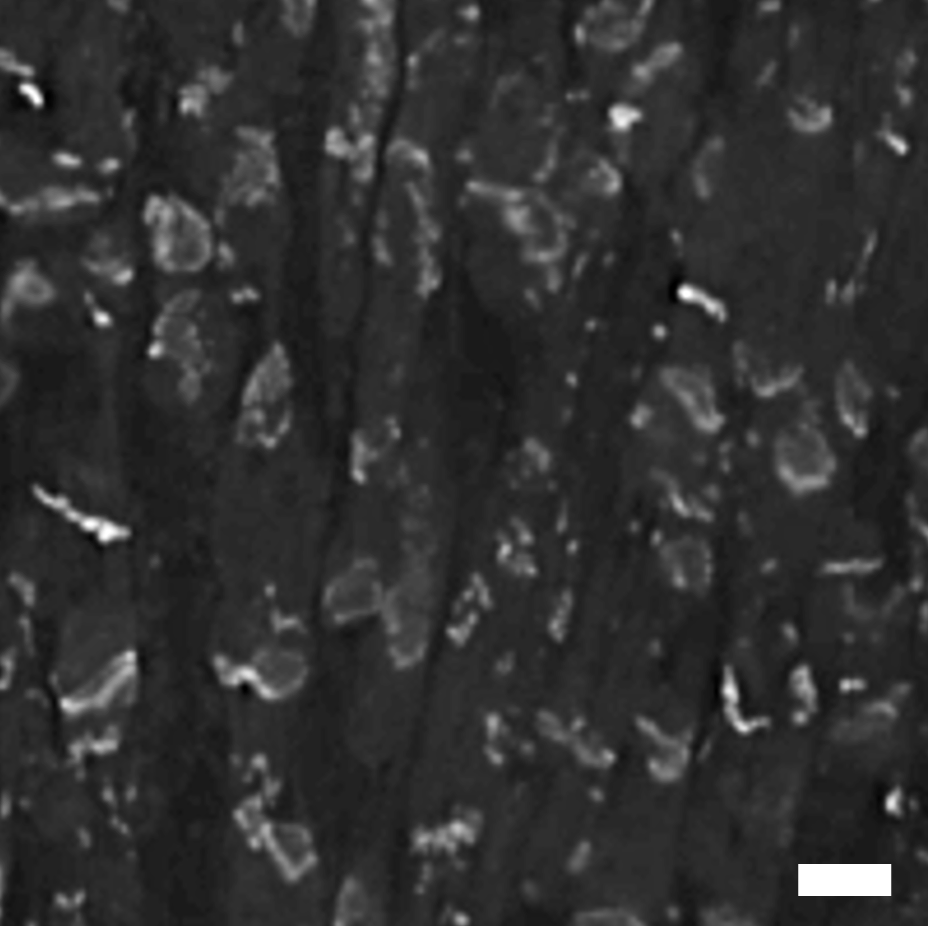

Supplement: Supplementary file 7 — Source data Fig. 6 [file 44319_2024_249_MOESM7_ESM.zip › FIG6/FIG.6D/DMD.tiff]

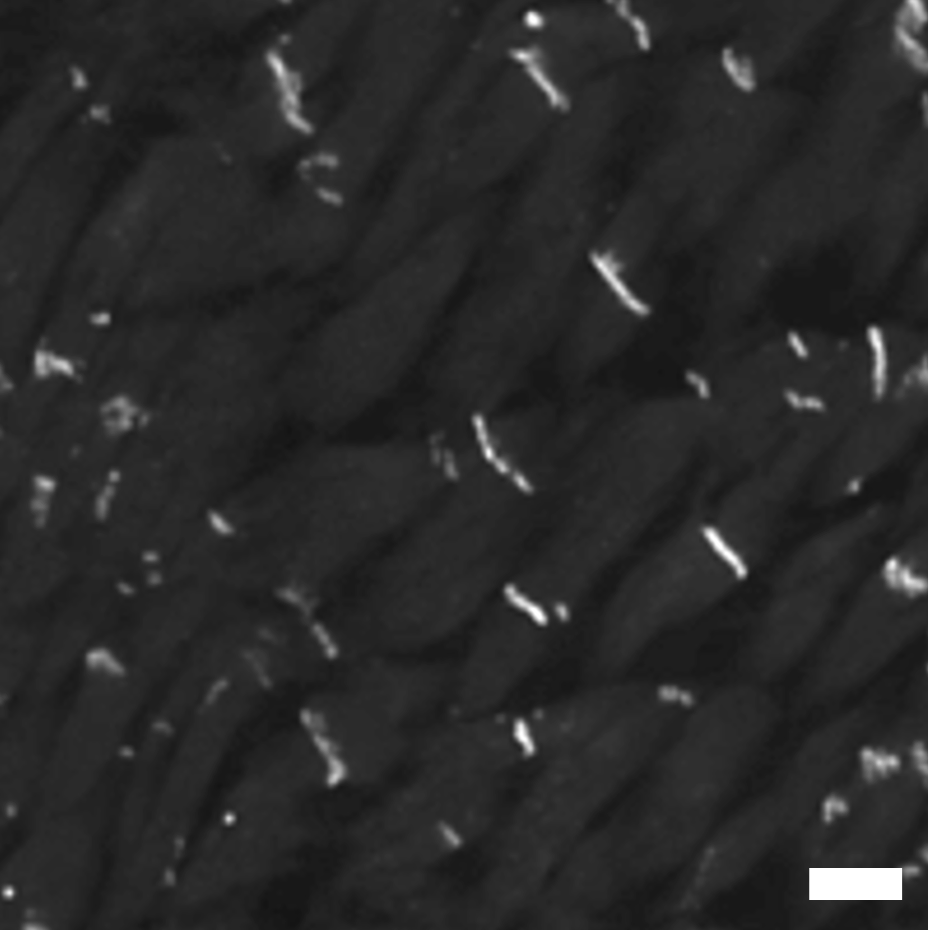

Supplement: Supplementary file 7 — Source data Fig. 6 [file 44319_2024_249_MOESM7_ESM.zip › FIG6/FIG.6D/WT.tiff]

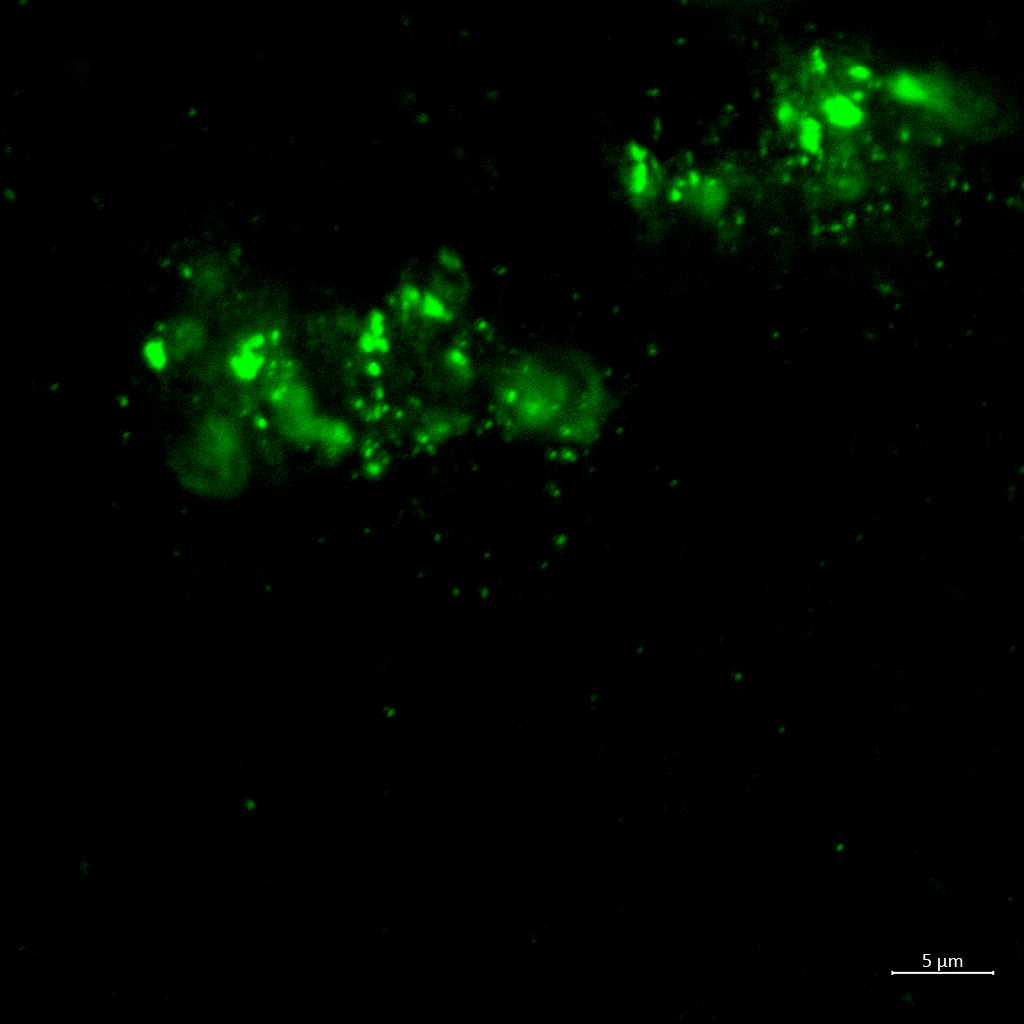

Supplement: Supplementary file 7 — Source data Fig. 6 [file 44319_2024_249_MOESM7_ESM.zip › FIG6/FIG.6H/BMD 63X_EGFP-T3.tif]

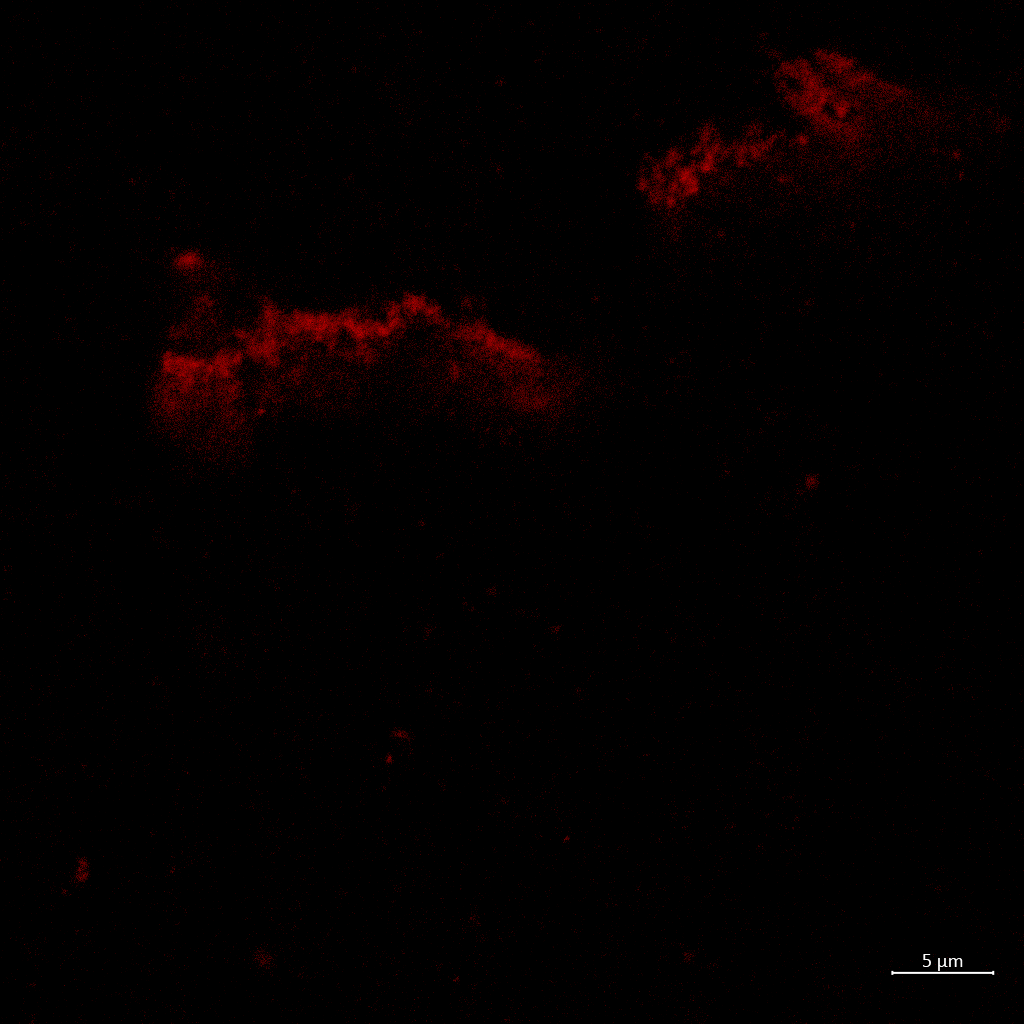

Supplement: Supplementary file 7 — Source data Fig. 6 [file 44319_2024_249_MOESM7_ESM.zip › FIG6/FIG.6H/BMD 63X_DsRed-T2.tif]

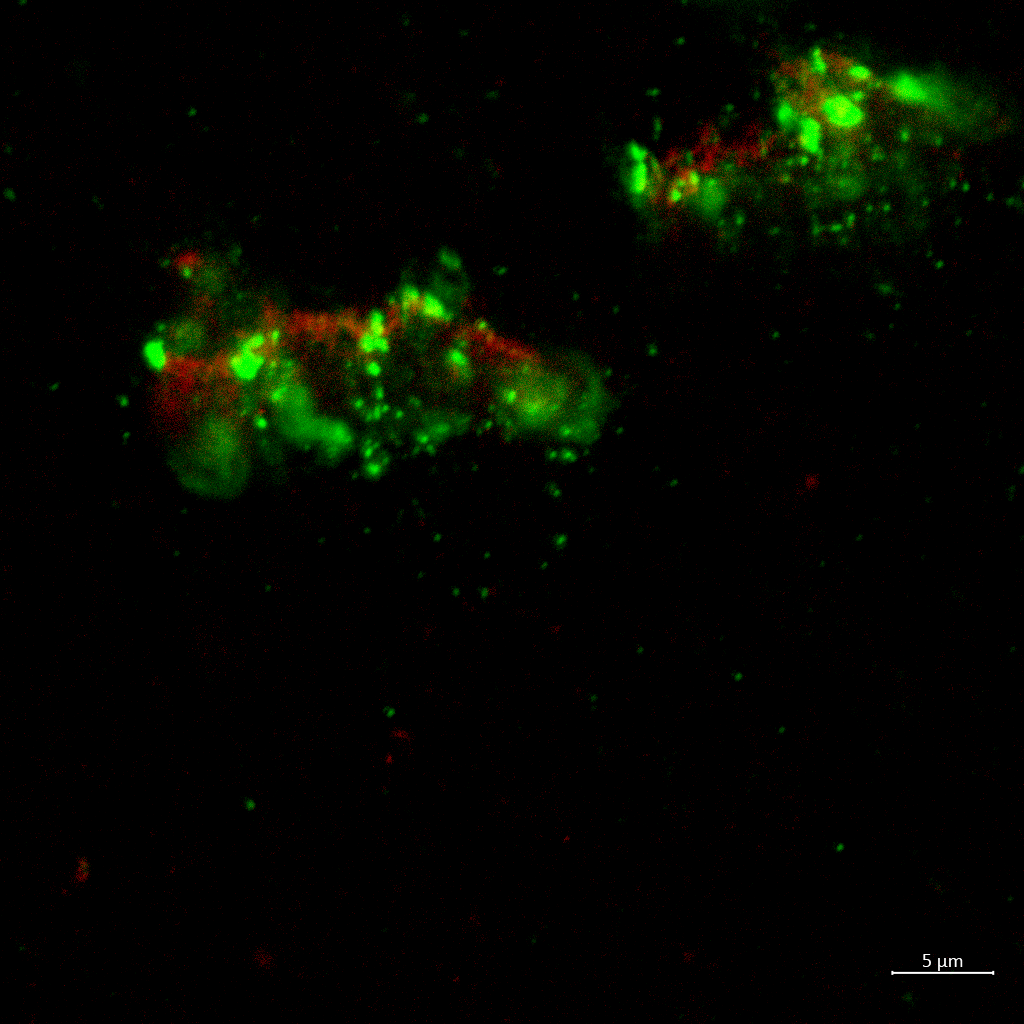

Supplement: Supplementary file 7 — Source data Fig. 6 [file 44319_2024_249_MOESM7_ESM.zip › FIG6/FIG.6H/BMD 63X.tif]

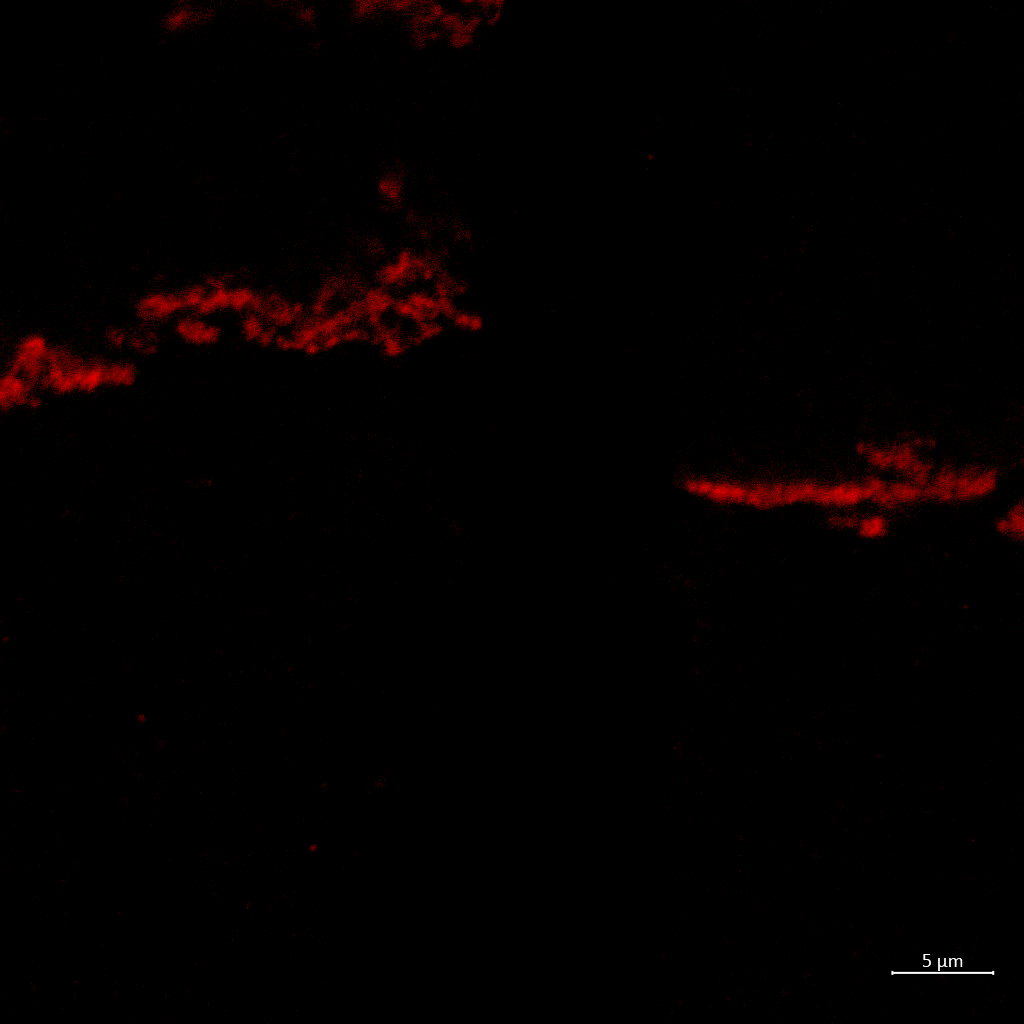

Supplement: Supplementary file 7 — Source data Fig. 6 [file 44319_2024_249_MOESM7_ESM.zip › FIG6/FIG.6I/DMD 63X _DsRed-T2.tif]

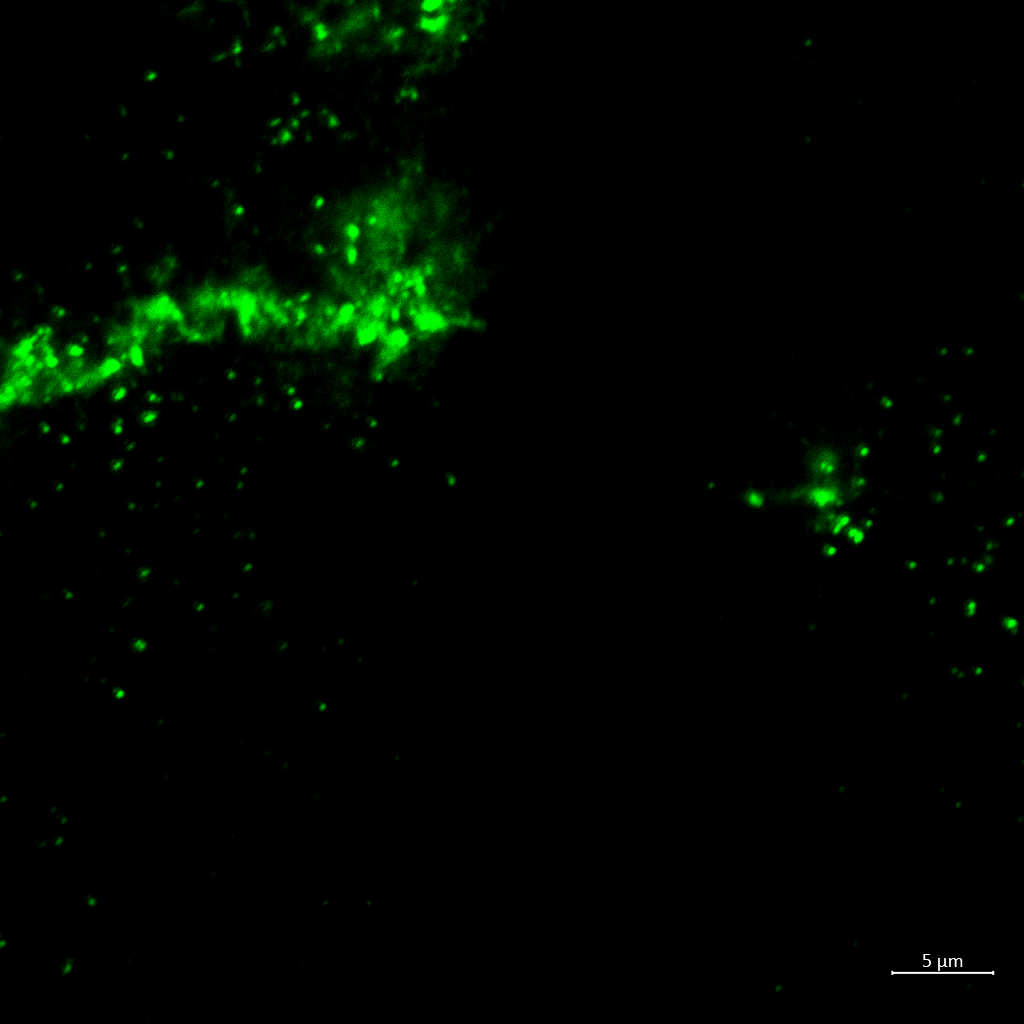

Supplement: Supplementary file 7 — Source data Fig. 6 [file 44319_2024_249_MOESM7_ESM.zip › FIG6/FIG.6I/DMD 63X _EGFP-T3.tif]

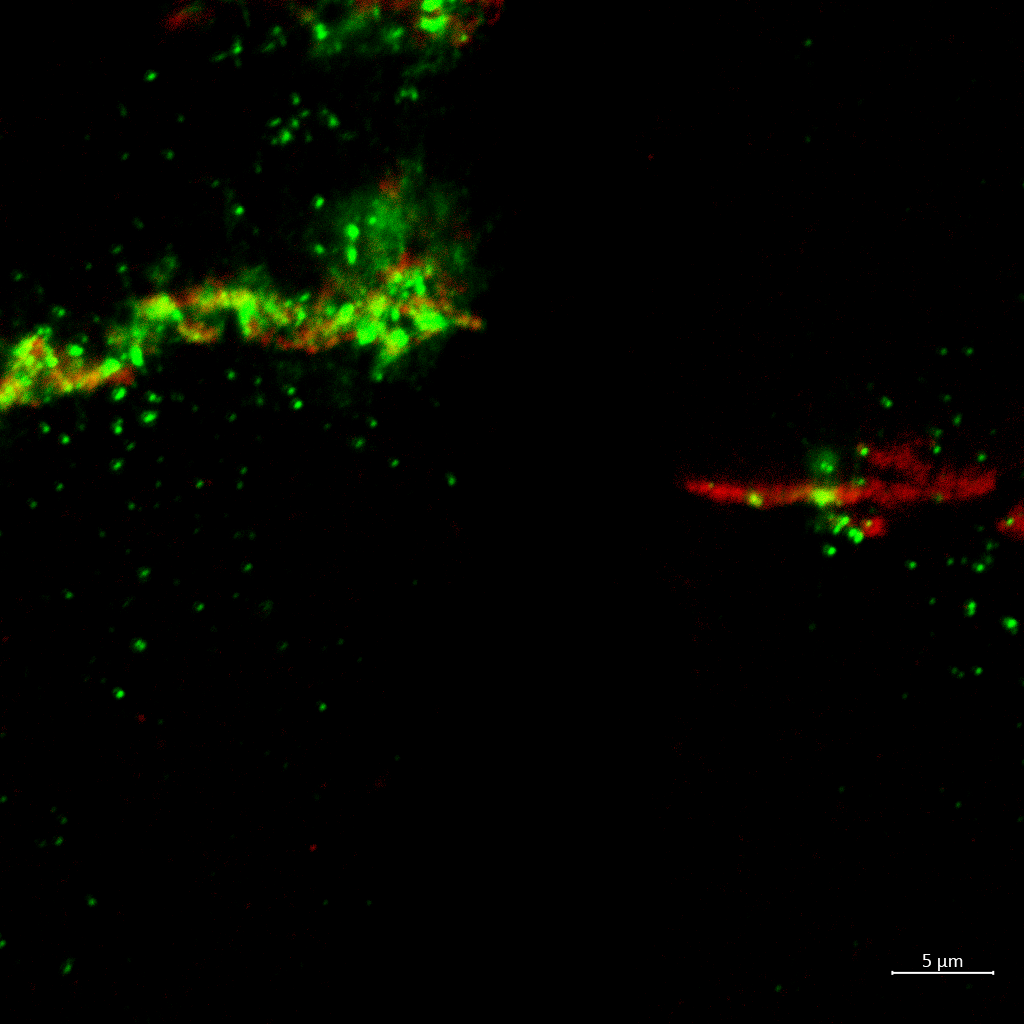

Supplement: Supplementary file 7 — Source data Fig. 6 [file 44319_2024_249_MOESM7_ESM.zip › FIG6/FIG.6I/DMD 63X .tif]

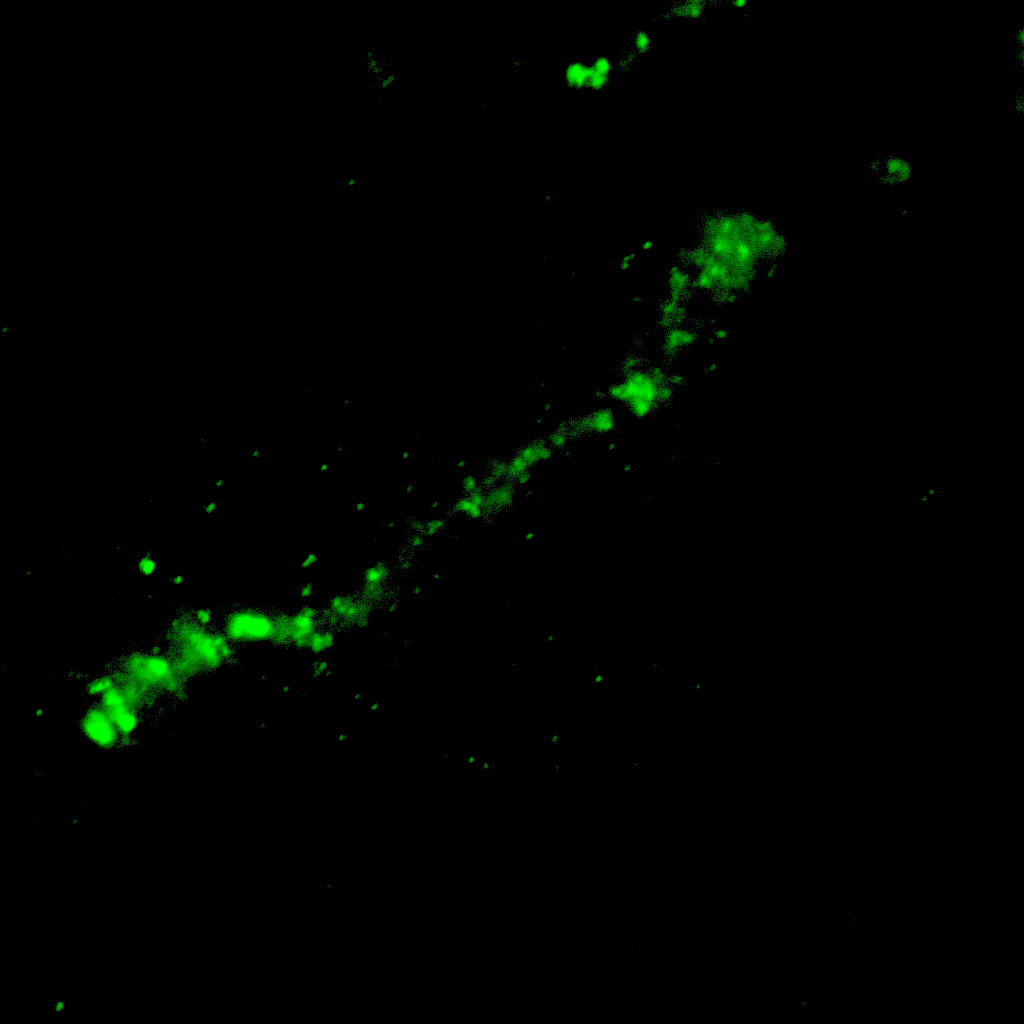

Supplement: Supplementary file 7 — Source data Fig. 6 [file 44319_2024_249_MOESM7_ESM.zip › FIG6/FIG.6G/WT 63X 10_EGFP-T3.tif]

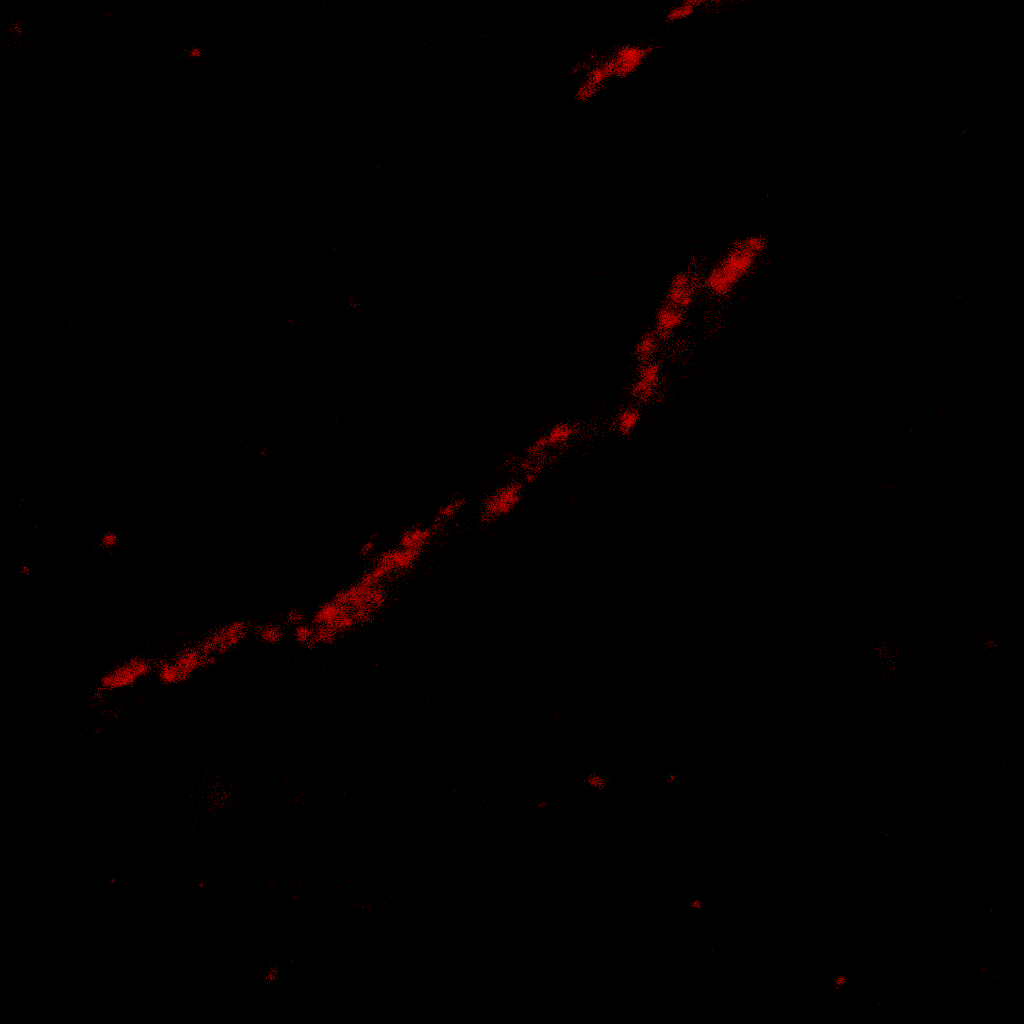

Supplement: Supplementary file 7 — Source data Fig. 6 [file 44319_2024_249_MOESM7_ESM.zip › FIG6/FIG.6G/WT 63X 10_DsRed-T2.tif]

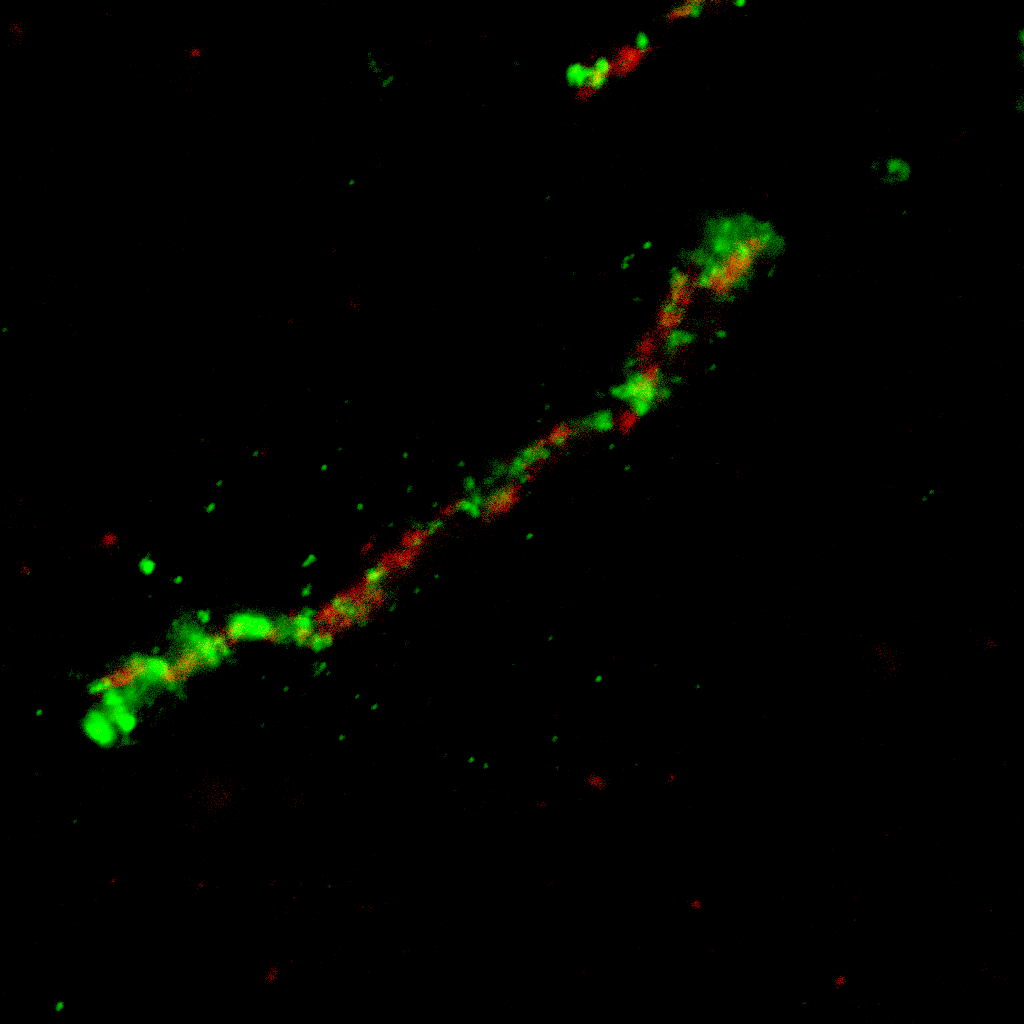

Supplement: Supplementary file 7 — Source data Fig. 6 [file 44319_2024_249_MOESM7_ESM.zip › FIG6/FIG.6G/WT 63X 10.tif]

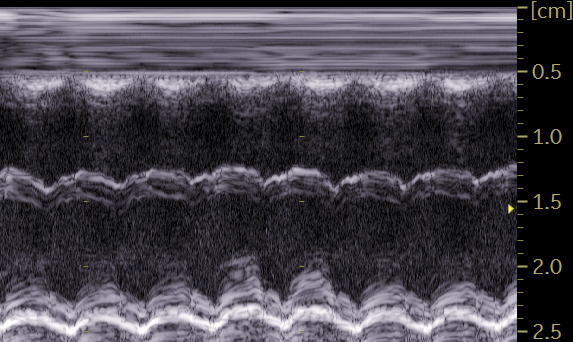

Supplement: Supplementary file 8 — Source data Fig. 7 [file 44319_2024_249_MOESM8_ESM.zip › FIG7/FIG.7A/FIG.7A_BMDM3.tiff]

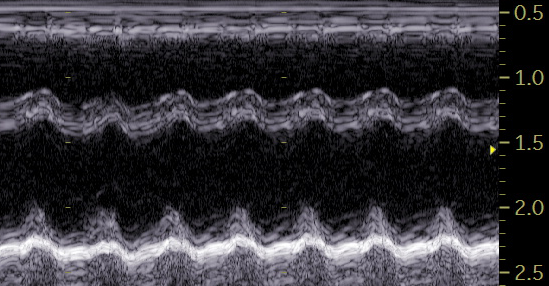

Supplement: Supplementary file 8 — Source data Fig. 7 [file 44319_2024_249_MOESM8_ESM.zip › FIG7/FIG.7A/FIG.7A_DMDM3.tiff]

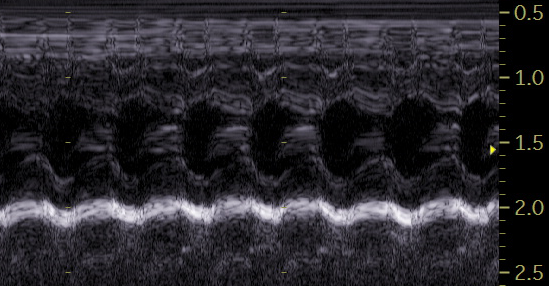

Supplement: Supplementary file 8 — Source data Fig. 7 [file 44319_2024_249_MOESM8_ESM.zip › FIG7/FIG.7A/FIG.7A_WTM2.tiff]
