# Supplementary material for: Progressive cardiomyopathy with intercalated disc disorganization in a rat model of Becker dystrophy
Source: EMBO Rep. 2024 Oct 2;25(11):4898–920. doi: 10.1038/s44319-024-00249-9 (PMC11549483; doi:10.1038/s44319-024-00249-9)
Supplement: Supplementary file 9 — Expanded View Figures [file 44319_2024_249_MOESM9_ESM.pdf]

Expanded View Figures

Figure EV1. Dystrophin immunofluorescence staining.

(**A**) Localization of dystrophin epitopes recognized by the antibodies used in the study (NCL-DYS1 and NCL-DYS2). The BMD deletion of exons 45–47 at the level of the nNOS binding site is highlighted. ABD, Actin-binding domain; CR, Cysteine rich domain; CT, C-terminal domain; R, rod spectrin-like repeats. (**B**, **C**) Representative images of DYS1 (red), DYS2 (purple), and Laminin (cyan) immunostaining on TA (**B**) and heart (**C**) of WT, BMD, and DMD rats at 11 months. Scale bar 20 µm. (**D**, **E**) Quantification of dystrophin intensity detected by DYS1 (**D**) and DYS2 (**E**) antibodies in both TA and heart of WT, BMD, and DMD rats at 11 months. One-way ANOVA, $n = 3$, corresponding to the number of independent rats. Data are represented as mean ± SD.

© The Author(s)

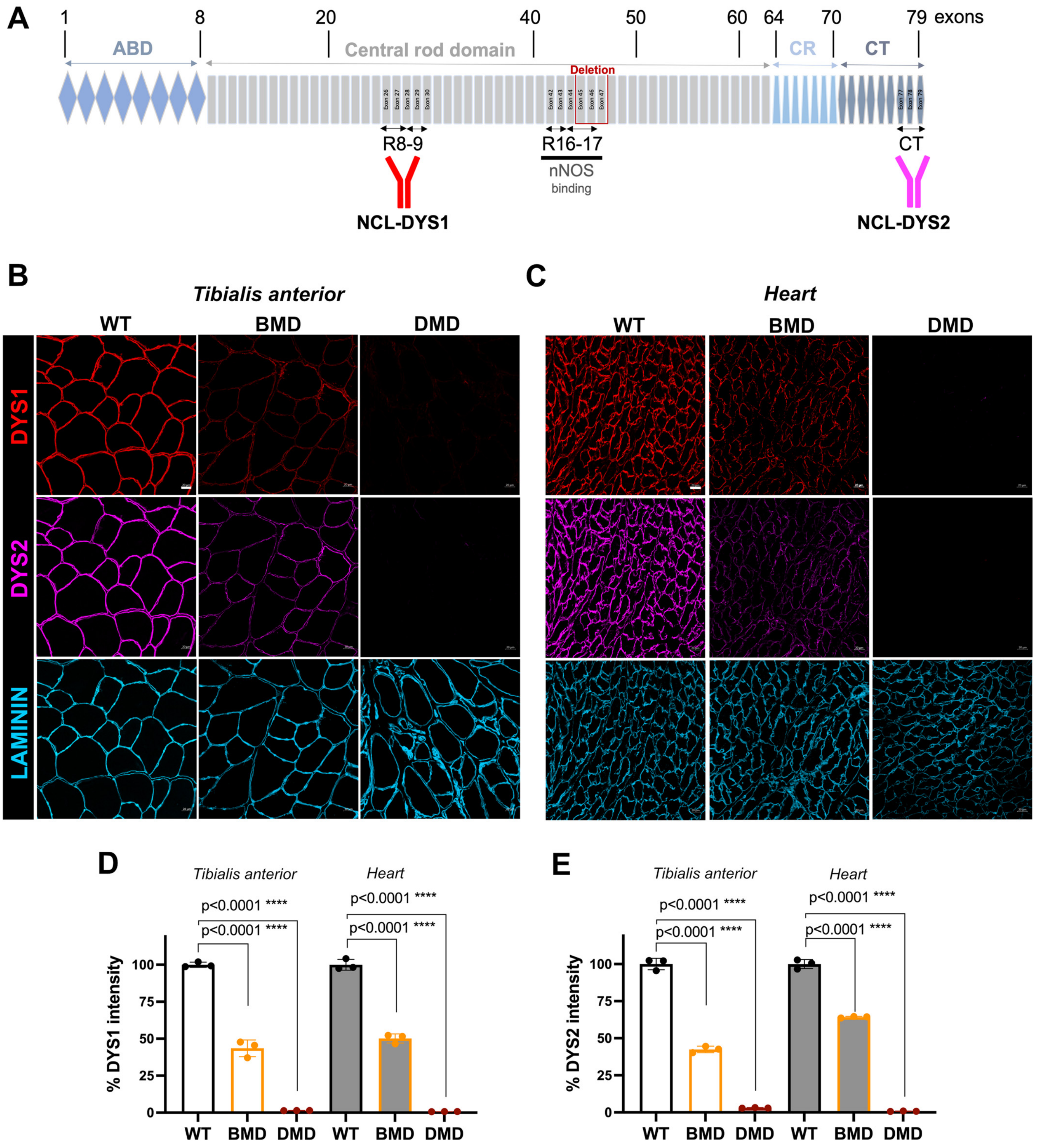
A
1
8
20
40
50
60
64
70
79 exons
ABD
Central rod domain
CR
CT
Deletion
R8-9
R16-17
nNOS
binding
CT
NCL-DYS1
NCL-DYS2
B
Tibialis anterior
WT
BMD
DMD
DYS1
DYS2
LAMININ
C
Heart
WT
BMD
DMD
D
Tibialis anterior
Heart
p<0.0001 ****
p<0.0001 ****
p<0.0001 ****
p<0.0001 ****
% DYS1 intensity
100
75
50
25
0
WT BMD DMD WT BMD DMD
E
Tibialis anterior
Heart
p<0.0001 ****
p<0.0001 ****
p<0.0001 ****
p<0.0001 ****
% DYS2 intensity
100
75
50
25
0
WT BMD DMD WT BMD DMD

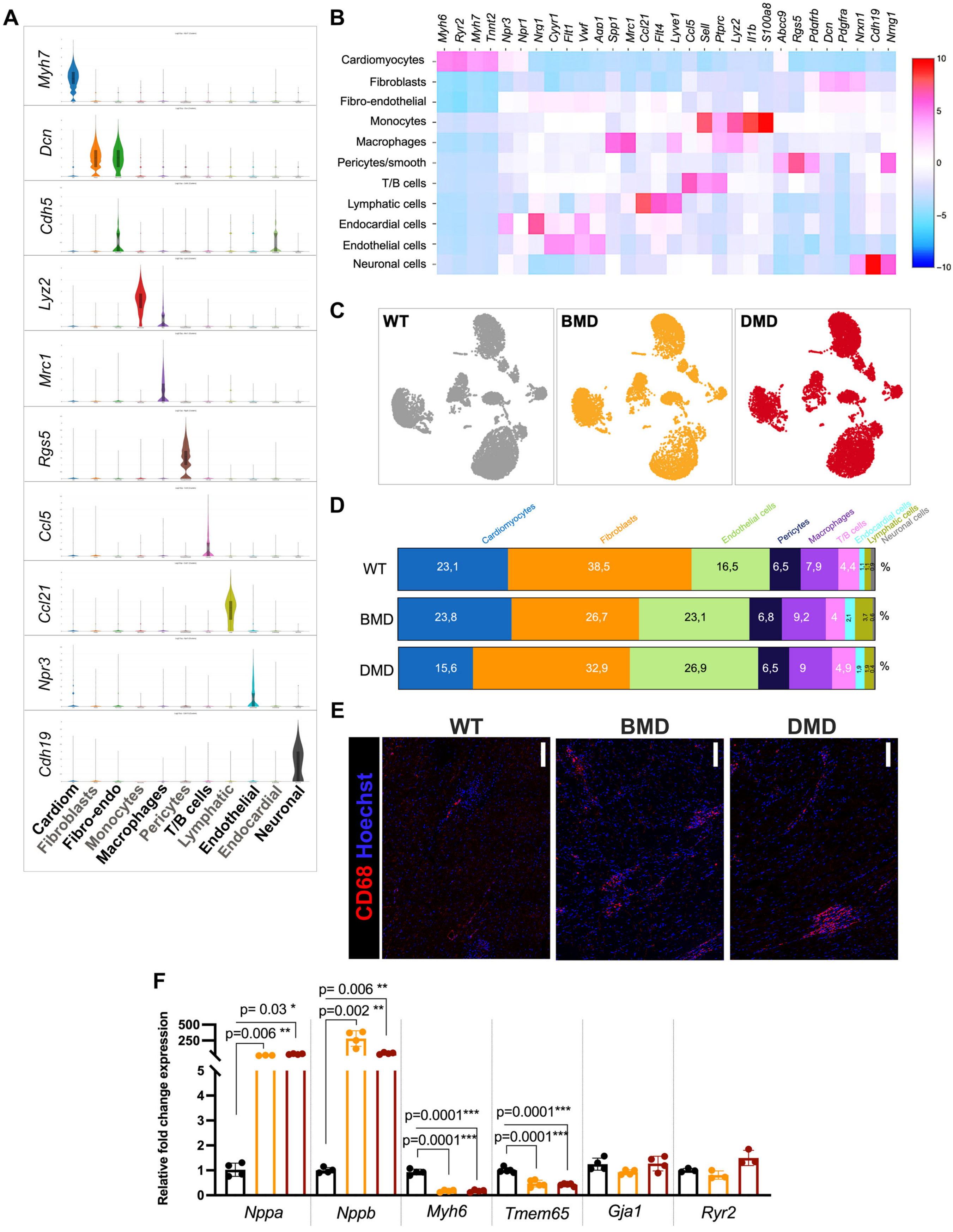
A
Myh7
Dcn
Cdh5
Lyz2
Mrc1
Rgs5
Ccl5
Ccl21
Npr3
Cdh19
Cardiom
Fibroblasts
Fibro-endo
Monocytes
Macrophages
Pericytes
T/B cells
Lymphatic
Endothelial
Endocardial
Neuronal
B
Myh6
Ryr2
Myh7
Tnnt2
Npr3
Npr1
Nrg1
Cyyr1
Flt1
Vwf
Aqp1
Spp1
Mrc1
Ccl21
Flt4
Lyve1
Ccl5
Sell
Ptprc
Lyz2
Il1b
S100a8
Abcc9
Rgs5
Pdgfrb
Dcn
Pdgfra
Nrxn1
Cdh19
Nrng1
Cardiomyocytes
Fibroblasts
Fibro-endothelial
Monocytes
Macrophages
Pericytes/smooth
T/B cells
Lymphatic cells
Endocardial cells
Endothelial cells
Neuronal cells
10
5
0
−5
−10
C
WT
BMD
DMD
D
Cardiomyocytes
Fibroblasts
Endothelial cells
Pericytes
Macrophages
T/B cells
Endocardial cells
Lymphatic cells
Neuronal cells
WT 23,1 38,5 16,5 6,5 7,9 4,4 %
BMD 23,8 26,7 23,1 6,8 9,2 4 2,1 3,7 %
DMD 15,6 32,9 26,9 6,5 9 4,9 1,9 %
E
WT
BMD
DMD
CD68 Hoechst
F
Relative fold change expression
500
250
5
4
3
2
1
0
p= 0.03 *
p=0.006 **
p= 0.006 **
p=0.002 **
p=0.0001***
p=0.0001***
p=0.0001***
p=0.0001***
Nppa
Nppb
Myh6
Tmem65
Gja1
Ryr2

 © The Author(s)

◀ **Figure EV2. snRNAseq analysis and validations.**

(**A**) Violin plots showing the expression of Myh7 as marker of cardiomyocytes, Dcn for fibroblasts and fibro-endothelial cells, the latest marked also by the expression of Cdh5, Lyz2 for monocytes, Mrc1 for macrophages, Rgs5 for pericytes, Ccl5 for T and B cells, Ccl21 for lymphatic cells, Npr3 for endothelial cells and Cdh19 for neuronal cells. (**B**) Heat map of expression of differentially expressed genes identified from snRNA-seq. (**C**) UMAP representation of WT, BMD, and DMD snRNAseq datasets. (**D**) Proportions of cells (%) identified per sample. (**E**) CD68 immunostaining on left ventricles from WT, BMD, and DMD rats at 11 months of age. Scale bar 100 μm. (**F**) RT-qPCR analysis of Nppa, Nppb, Myh6, Tmem65, Gja1, and Ryr2 on ventricle tissue RNA extraction from 12-month-old WT, BMD, and DMD rats. One-way ANOVA, $n = 3$–4, corresponding to the number of independent rats. Data are represented as mean ± SD.

© The Author(s)
